# Supplementary material for: Extreme potential photocatalysis enabled by spin-exchange Auger processes in magnetic-doped quantum dots
Source: Nat Commun. 2025 Jun 6;16:5280. doi: 10.1038/s41467-025-60659-8 (PMC12144220; doi:10.1038/s41467-025-60659-8)
Supplement: Supplementary file 1 — Supplementary Information [file 41467_2025_60659_MOESM1_ESM.pdf]

Supplementary Information for

Extreme Potential Photocatalysis Enabled by Spin-Exchange Auger Processes in Magnetic-Doped Quantum Dots

Qinxuan Cao,[1] Jianning Feng,[1] Kezhou Fan,[2] Shuting Zhang,[1] Jinzhong Zhang[3], Baixu Ma,[4] Jie Xue,[1] Xin Li,[1] Kang Wang,[3] Lizhi Tao,[4] Aleksandr Sergeev,[2] Ye Yang,[3,5] Kam Sing Wong,[2] Yong Huang,[1] and Haipeng Lu[*1,6,7]

[1] Department of Chemistry, The Hong Kong University of Science and Technology, Clear Water Bay, Kowloon, 999077, Hong Kong, China (SAR)

[2] Department of Physics, The Hong Kong University of Science and Technology, Clear Water Bay, Kowloon, 999077, Hong Kong, China (SAR)

[3] State Key Laboratory of Physical Chemistry of Solid Surfaces, College of Chemistry and Chemical Engineering, Xiamen University, Xiamen, 361005, China

[4] Department of Chemistry, Department of Chemistry, Southern University of Science and Technology, Shenzhen, Guangdong, 518055 China

[5] Innovation Laboratory for Sciences and Technologies of Energy Materials of Fujian Province (IKKEM), Xiamen, 361005, China

[6] Energy Institute, The Hong Kong University of Science and Technology, Clear Water Bay, Kowloon, 999077, Hong Kong, China (SAR)

[7] Hong Kong Branch of Chinese National Engineering Research Center for Tissue Restoration and Reconstruction, The Hong Kong University of Science and Technology, Clear Water Bay, Kowloon, 999077, Hong Kong, China (SAR)

Corresponding Author

Haipeng Lu[*]: haipenglu@ust.hk

Table of contents:

I. Note S1: Calculation of Number of Mn^{2+} dopants Per QDs
II. Note S2: Calculation of Exciton Number Per QDs.
III. General procedures for preparation of substrates **1b, 3a-3c, 5, 9a-9e, 11**.
IV. General Reaction Procedures
V. Isolation and Purification Procedures
VI. General procedures for yield determination by GC or NMR.
VII. Preparation and Characterization data of products.
VIII. Sequential Substitution Reaction Procedures
IX. Characterization of QDs: PXRD, UV-vis absorption, TEM, EDS, TRPL, and ICP data
X. ^{1}H NMR data
XI. References

I. Note 1: Calculation of the Number of Mn^{2+} dopants Per QDs

The volume of quantum dots is calculated according to following equation:

$$V_{CdS} = \frac{4}{3}\pi r_{CdS}^3 \quad (1)$$

$$V_{CdS/ZnS} = \frac{4}{3}\pi r_{CdS/ZnS}^3 \quad (2)$$

The r_{CdS} could be obtained from the exciton peak of CdS/ZnS QDs according to previously reported experimental equation[1], which is ~2.8 nm when exciton peak located at 461 nm. The $r_{CdS/ZnS}$ is measured from TEM images which is 3.35 nm.

The volume of ZnS shell is

$$V_{ZnS} = V_{CdS/ZnS} - V_{CdS} = 157.5 - 91.95 = 65.55\, nm^3 \quad (3)$$

The volume of CdS unit cell

$$V_{CdS,0} = a_{CdS}^3 = 0.195\, nm^3 \quad (4)$$

The volume of ZnS unit cell

$$V_{ZnS,0} = a_{ZnS}^3 = 0.157\, nm^3 \quad (5)$$

Here, a_{CdS} is the lattice parameter of zinc blende CdS (5.8 Å) and a_{ZnS} is the lattice parameter of ZnS (5.4 Å).

One CdS QD contains $N_{CdS} = \frac{V_{CdS}}{V_{CdS,0}} = 471.5$ CdS cells, and the number of CdS atoms is $N_{Cd} = N_{CdS} * 4 = 1886$, as there are 4 Cd atoms per CdS cell.

One CdS/ZnS QD contains $\frac{V_{ZnS}}{V_{ZnS,0}} = 417.5$ ZnS cells, the number of Zn atoms is $N_{Zn} = N_{ZnS} * 4 = 1670$, as there are 4 Zn atoms per ZnS cell.

According to ICP result the concentration of Mn^{2+}:

$$C_{Mn,doped} = \frac{C_{Mn,ICP}}{C_{Cd,ICP}} = \frac{0.275}{191.4} = 0.56\%$$

Therefore, per CdS QDs contains $N_{Mn} = N_{CdS} * C_{Mn,doped} \approx 10.5$.

Theoretically, the ratio of $N_{Cd}\ to\ N_{Zn} = \frac{N_{Cd}}{N_{Zn}} = 1.13$, which is close to the experimentally determined $N_{Cd}\ to\ N_{Zn}$ ratio by TEM-EDS and ICP measurement.

II. Note 2: calculation of exciton number per QDs <N>

<N> under femtosecond laser per pulse:

According to the Poisson distribution of initial photon occupancies in NCs, the probability of a NC containing n e-h pairs is determined by $P_n = \frac{\langle N_0 \rangle^n}{n!} e^{-\langle N_0 \rangle}$, where $\langle N_0 \rangle = J\sigma$ is the initially generated number of e-h pairs per NC on average[2]. Here, J refers to the pump fluence (i.e., number of photons per unit area) given by $\frac{P}{fh\upsilon\pi r^2}$, with P, f, $h\upsilon$ and r being the excitation power, repetition frequency, incident photon energy and beam spot radii, respectively. σ refers to the absorption cross section. When the delay time after excitation is sufficiently long (> ns) such that the multicarrier/multiexciton processes (e.g., Auger recombination/exciton-exciton annihilation) complete, the excited NCs will undergo single exciton recombination. Therefore, the late-time TA bleaching amplitude is proportional to the occupation probabilities of photoexcited NCs such that $\frac{\Delta T}{T} \propto \left(1 - e^{-\langle N_0 \rangle}\right)$ [3]. Here, we fit the pump-fluence-dependent TA bleaching amplitude excited at 370 nm at the delay time of 1.5 ns using the abovementioned model to obtain $\sigma_{370\ nm}$. The σ at other wavelength (σ_λ) is estimated by comparing the absorbance (OD) ratio, such that $\frac{\sigma_\lambda}{\sigma_{370\ nm}} = \frac{Abs_\lambda}{Abs_{370\ nm}}$.

<N> under cw-LED per second:

We calculated the average excitation rate (<*N*> = σ × q_p, where q_p is the photon flux, and σ is the absorption cross section) under different photon flux conducted for photocatalytic experiments. We also calculated the average excitation rate for each QDs to generate Mn^{2+} excited state <N_{Mn}> within the radiative lifetime of Mn^{2+} (^{4}T) by <N_{Mn*}> = <*N*> × τ_{Mn}, where τ_{Mn} is found as 2.6 ms.

Table S1 Calculated average excitation rate in doped QDs under experimental conditions

I (mW/cm^2)	q_p	<*N*> (s^{-1})	<N_{Mn*}> (s^{-1})	$\sum_{i=2}^{\infty} P(i)$
0	*0*	0	0	0
5	1.14×10^{16}	43.57	0.11	0.006
37.5	8.60×10^{16}	326.83	0.85	0.209
75	1.72×10^{17}	653.67	1.70	0.507
112	2.56×10^{17}	976.15	2.54	0.720
150	3.44×10^{17}	1307.34	3.40	0.853
300	6.88×10^{17}	2614.68	6.80	0.99

III. General procedures for preparation and characterization data of substrates 1b, 3a-3c, 5, 9a-9e, 11.

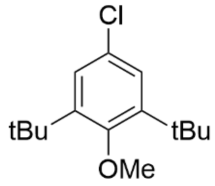

5-Chloro-1,3-bis(1,1-dimethylethyl)-2-methoxybenzene (2b)[CAS: 14804-28-5]was prepared based on Ref [4]

^{1}H NMR (400 MHz, $CDCl_3$) δ 7.19 (s, 2H), 3.67 (s, 3H), 1.41 (s, 18H).

^{13}C NMR (101 MHz, $CDCl_3$) δ 158.2, 145.6, 128.1, 126.5, 64.4, 36.0, 31.9, 30.7.

HRMS: calculated m/z for $C_{15}H_{24}ClO^+$ [$(M+H)^+$] 255.1516; found 255.1563.

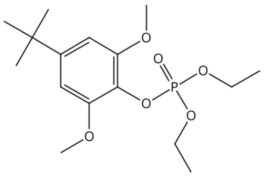

4-(tert-butyl)-2,6-dimethoxyphenyl diethyl phosphate (3a)[CAS: 80754-02-5] was prepared based on Ref [5].

^{1}H NMR (400 MHz, $CDCl_3$) δ 6.59 (d, J = 0.7 Hz, 2H), 4.43-4.08 (m, 4H), 3.86 (s, 6H), 1.39 (td, J = 7.1, 1.2 Hz, 6H), 1.29 (s, 9H).

^{13}C NMR (101 MHz, $CDCl_3$) δ 151.36 (d, J = 3.6 Hz), 148.6, 127.6, 102.8, 64.3 (d, J = 5.9 Hz), 56.2, 35.1, 31.5, 16.2 (d, J = 7.4 Hz).

HRMS: calculated m/z for $C_{16}H_{27}O_6PNa^+$ [$(M+Na)^+$] 369.1448; found 369.1445.

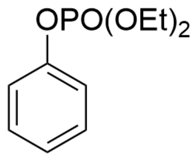

Diethyl phenyl phosphate(3b) [CAS: 2510-86-3] was prepared based on Ref [5].

^{1}H NMR (400 MHz, $CDCl_3$) δ 7.33 (t, J = 7.9 Hz, 2H), 7.21 (d, J = 8.0 Hz, 2H), 7.16 (t, J = 7.4 Hz, 1H), 4.42 – 4.10 (m, 4H), 1.35 (t, J = 7.1 Hz, 6H).

^{13}C NMR (101 MHz, $CDCl_3$) δ 150.8 (d, J = 6.9 Hz), 129.7, 125.0, 120.0 (d, J = 5.0 Hz), 64.6 (d, J = 6.0 Hz), 16.1 (d, J = 6.6 Hz).

HRMS: calculated m/z for $C_{10}H_{16}O_4P$ [M+Na]$^+$ 253.0606; found 253.0610.

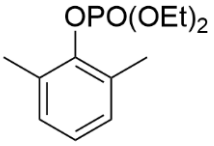

2,6-Dimethylphenyl diethyl phosphate (3c) [CAS: 39604-15-4] was prepared based on Ref [5].

^{1}H NMR (400 MHz, $CDCl_3$) δ 7.10 – 6.90 (m, 3H), 4.48 – 4.04 (m, 4H), 2.37 (s, 6H), 1.47 – 1.21 (m, 6H).

^{13}C NMR (101 MHz, $CDCl_3$) δ148.1 (d, J = 8.1 Hz), 130.3 (d, J = 3.3 Hz), 129.0 (d, J = 1.8 Hz), 125.1 (d, J = 2.1 Hz), 64.4 (d, J = 6.0 Hz), 17.2, 16.1 (d, J = 6.9 Hz).

HRMS: calculated m/z for $C_{12}H_{20}O_4P$ [M+H]$^+$ 259.1099; found 259.1109.

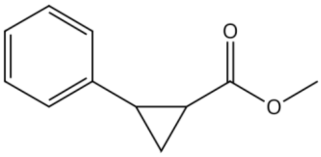

trans-(+/-)-methyl-2-phenyl-1-cyclopropanecarboxylate (5) [CAS: 20030-70-0]was prepared based on Ref [6].

^{1}H NMR (400 MHz, $CDCl_3$) δ 7.29 (d, J = 7.3 Hz, 2H), 7.24 – 7.17 (m, 1H), 7.13 – 7.06 (m, 2H), 3.72 (s, 3H), 2.57 – 2.48 (m, 1H), 1.94 – 1.87 (m, 1H), 1.65 – 1.60 (m, 1H), 1.33 (ddd, J = 8.4, 6.5, 4.5 Hz, 1H).

^{13}C NMR (101 MHz, $CDCl_3$) δ 174.0, 140.1, 126.6, 126.3, 52.0, 26.4, 24.0, 17.1.

HRMS: calculated m/z for $C_{11}H_{12}O_2$ 177.0921; found 177.0909.

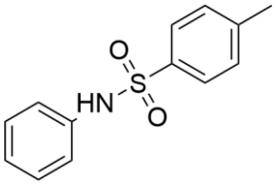

***N*-Phenyl-*p*-toluenesulfonamide (9a)[CAS: 68-34-8]** was prepared based on Ref[7].

^{1}H NMR (400 MHz, $CDCl_3$) δ 7.65 (d, J = 8.4 Hz, 2H), 7.22 (dd, J = 8.2, 2.9 Hz, 4H), 7.15 – 7.08 (m, 1H), 7.08 – 7.03 (m, 2H), 6.61 (s, 1H), 2.38 (s, 3H).

^{13}C NMR (101 MHz, $CDCl_3$) δ 143.9, 136.5, 136.1, 129.7, 129.3, 127.3, 125.4, 121.7, 21.6.

HRMS: calculated m/z for $C_{13}H_{13}NO_2SNa^+$ [M+Na]$^+$ 270.0565; found 270.0554.

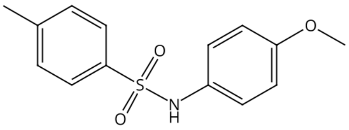

N-(4-methoxyphenyl)-p-toluenesulfonamide (9b)[CAS:1150-26-1] was prepared based on Ref [6].

^{1}H NMR (400 MHz, $CDCl_3$) δ 7.57 (d, J = 8.4 Hz, 2H), 7.21 (d, J = 7.9 Hz, 2H), 6.98 – 6.92 (m, 2H), 6.79 – 6.74 (m, 2H), 6.18 (br s, 1H), 3.76 (s, 3H), 2.39 (s, 3H).

^{13}C NMR (101 MHz, $CDCl_3$) 158.0, 143.8, 136.1, 129.0, 128.5 (d, J = 335.1 Hz), 125.5, 114.5, 55.5, 21.6.

HRMS: calculated m/z for $C_{14}H_{15}NO_3SNa^+$ [$(M+Na)^+$] 300.0676; found 300.0674.

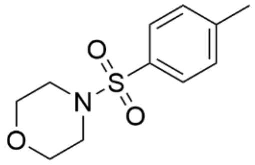

4-[(4-Methylphenyl)sulfonyl]morpholine (9c) [CAS: 6339-26-0]

^{1}H NMR (400 MHz, $CDCl_3$) δ 7.64 (d, J = 8.3 Hz, 2H), 7.38 – 7.31 (m, 2H), 3.82 – 3.67 (m, 4H), 3.02 – 2.92 (m, 4H), 2.45 (s, 3H).

^{13}C NMR (101 MHz, $CDCl_3$) δ 144.0, 132.1, 129.8, 127.9, 66.1, 46.0, 21.6.

HRMS: calculated m/z for $C_{11}H_{16}NO_3S^+$ $[M+H]^+$ 242.0851; found 242.0862.

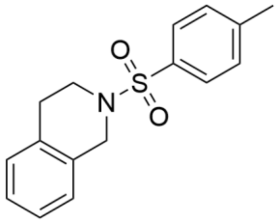

2-Tosyl-1,2,3,4-tetrahydroisoquinoline (9d) [CAS: 20335-69-7]

^{1}H NMR (400 MHz, $CDCl_3$) δ 7.73 (d, J = 8.4 Hz, 2H), 7.42 – 7.28 (m, 2H), 7.22 – 7.12 (m, 2H), 7.10 – 6.95 (m, 2H), 4.25 (s, 2H), 3.35 (t, J = 5.9 Hz, 2H), 2.94 (d, J = 5.9 Hz, 3H), 2.42 (s, 3H).

^{13}C NMR (101 MHz, $CDCl_3$) δ 143.7, 133.3, 133.1, 131.7, 129.7, 128.8, 127.8, 126.7, 126.4 (d, J = 3.4 Hz), 47.5, 43.7.

HRMS: calculated m/z for $C_{16}H_{18}NO_2S^+$ $[M+H]^+$ 288.1058; found 288.1042.

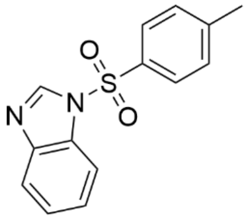

1-[(4-Methylphenyl)sulfonyl]-1H-benzimidazole (9e) [CAS: 15728-44-6]

^{1}H NMR (400 MHz, $CDCl_3$) δ 8.42 (s, 1H), 7.90 (t, J = 8.1 Hz, 3H), 7.80 (dd, J = 7.8, 1.5 Hz, 1H), 7.40 (td, J = 7.8, 1.5 Hz, 2H), 7.33 (d, J = 8.1 Hz, 2H), 2.41 (s, 3H).

^{13}C NMR (151 MHz, $CDCl_3$) δ146.3, 143.7, 141.2, 134.5, 130.4, 127.3, 125.7, 124.9, 121.0, 112.5, 21.7.

HRMS: calculated m/z for $C_{14}H_{13}N_2O_2S^+$ $[M+H]^+$ 273.0698; found 273.0696.

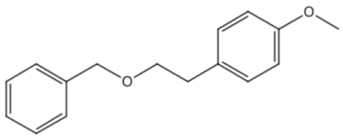

1-Methoxy-4-[2 (phenylmethoxy)ethyl]benzene (11) [CAS: 1258760-02-9]

^{1}H NMR (400 MHz, $CDCl_3$) δ 7.42 – 7.29 (m, 5H), 7.17 (d, J = 8.6 Hz, 2H), 6.86 (d, J = 8.6 Hz, 2H), 4.55 (s, 2H), 3.81 (s, 3H), 3.68 (t, J = 7.2 Hz, 2H), 2.90 (t, J = 7.2 Hz, 1H), 1.58 (s, 2H).

^{1}H NMR (400 MHz, $CDCl_3$) δ 150.8, 129.7, 125.0, 120.0, 64.6 (d, J = 6.1 Hz), 16.10 (d, J = 6.7 Hz).

HRMS: calculated m/z for $C_{16}H_{19}O_2$ $[M+H]^+$ 243.1385; found 243.1390.

IV. General Reaction Procedures

General Procedure for hydrodefunctionalization of Aryl Electrophiles

A tube equipped with a PTFE coated magnetic stirring bar is charged with aryl chloride(**1a-1o**) or aryl phosphate ester(**3a-3c**) (0.25 mmol, 1 equiv.), and then introduced into a glove box filled with nitrogen. There, 1 mL DMPU solvent and 56 μL TAEA (0.375 mmol, 1.5 equiv.) are sequentially added into the tube. The mixture is stirred to dissolve reactants. Then 2.5 nmol QDs dispersed in hexane is added into the same tube. After stirring for several seconds, the mixture becomes homogeneous and clear. The tube is sealed to keep the N_2 atmosphere before being removed from the glove box. The tube is put upon the stir plate equipped with blue LEDs and fans for cooling. Then the reaction is irradiated under stirring (700 rpm) for 24 hours (150 mW/cm^2) or 55 hours (5 mW/cm^2).

General Procedure for Birch Reduction Procedure

A tube equipped with a PTFE coated magnetic stirring bar was introduced into a glove box filled with nitrogen. There, 0.1 mmol benzene (**7**), t-amyl alcohol (90 μL, 8.0 eq.), and $N(Me)_4OH$ (0.4 mL, 10 eq. (25% solution in MeOH)) were added. Then 2.5 nmol QDs dispersed in hexane was added into the same tube. The tube was sealed to keep the N_2 atmosphere before being removed from the glove box. The tube was put upon the stir plate equipped with blue LEDs and fans for cooling. Then the reaction was irradiated stirring (700 rpm) for 24h (under LED I with 150 mW/cm^2).

V. Isolation and Purification Procedures

Purification A. The resulting reaction mixture was diluted with 20 mL dichloromethane then filtered through a 1 cm thick celite, flash the celite 3 times with DCM. Next, diluted crude products were mixed with silica gel. Volatile solvents are removed under reduced pressure on a rotary evaporator. The resulting dry-loaded product was purified by column chromatography on silica or preparative thin-layer chromatography to provide the desired products.

Purification B. The resulting reaction mixture was poured into 50 mL water and extracted with 20 mL DCM 3 times. The organic layer was washed with 20 mL water and dried over anhydrous Na_2SO_4. Add silica gel into the filtered mixture, and volatile solvent was removed under reduced pressure on a rotary evaporator. The resulting dry-loaded product was purified by column chromatography on silica or preparative thin-layer chromatography to provide the desired products.

VI. General procedures for yield determination by GC or NMR.

^{1}H NMR analysis. After the reaction was stopped by exposed to air, 0.1 mmol (7 μl) CH_2Br_2 was added as internal standard. A 100 μL aliquot of the crude mixture was injected into 1 mL water, then extracted with $CDCl_3$ for NMR analysis. The yield could be calculated from the integration ratio of signals from products and CH_2Br_2 (~4.8 ppm).

GC-FID analysis. After the reaction was stopped by exposed to air, 0.5 mmol diphenyl ether was added as internal standard. A 100 µL aliquot of the crude mixture was mixed with DCM then filtered through PTFE membrane filter for GC-FID analysis.

VII. Preparation and Characterization data of Products.

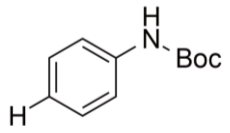

tert-butyl phenylcarbamate (2a) [CAS: 3422-01-3]

General procedure was followed using tert-butyl (4-chlorophenyl) carbamate (56.9 mg, 0.25 mmol, 1 equiv.). After 18h (under LED I with 150 mW/cm^2) or 55 h (under LED II with 5mW/cm^2), the reaction was quenched and purified according to Purification A (5% EtOAc/Hexanes), affording the product as a white solid (45.1 mg, 93% yield, LED I; 42.7 mg, 88% yield, LED II). Characterization data of **2a** is consistent with literature reports.[6]

^{1}H NMR (400 MHz, $CDCl_3$) δ 7.36 (d, J = 8.7 Hz, 2H), 7.32 – 7.23 (m, 2H), 7.03 (tt, J = 7.3, 1.2 Hz, 1H), 6.49 (s, 1H), 1.52 (s, 9H).

^{13}C NMR (101 MHz, $CDCl_3$) δ 152.8, 138.4, 129.1, 123.1, 118.6, 80.6, 28.4.

HRMS: calculated m/z for $C_{11}H_{15}NO_2$ 194.1186; found 194.1167.

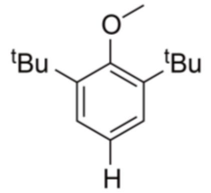

1,3-di-tert-butyl-5-chloro-2-methoxybenzene (2b) [CAS: 1516-95-6]

General procedure was followed using 1,3-di-tert-butyl-5-chloro-2-methoxybenzene (63.7 mg, 0.25 mmol, 1 equiv.). After 24h (under LED I with 150 mW/cm^2) or 55 h (under LED II with 5 mW/cm^2), the reaction was poured into water (50 mL) and extracted with Et_2O (3 × 25 mL). The combined organic layers were washed with 20 mL water twice and 25 mL saturated brine, dried over anhydrous Na_2SO_4. The solids are removed through filtration. After that, volatile solvent was removed under reduced pressure on a rotary evaporator affording an inseparable mixture of the product (57mg, 24h, LED I; 55 mg, 55 h, LED II). The yield of **2b** is calculated to be 79% (LED I) and 92.5% (50.9 mg, LED II) from the integration of NMR. Characterization data of **2b** is consistent with literature reports.[6]

^{1}H NMR (400 MHz, $CDCl_3$) δ 7.28 (d, J = 7.8 Hz, 2H), 7.04 (t, J = 7.8 Hz, 1H), 3.76 (s, 3H), 1.50 (s, 18H).

^{13}C NMR (101 MHz, $CDCl_3$) δ159.6, 143.7, 126.6, 123.0, 64.3, 35.8, 32.2.

HRMS: calculated m/z for $C_{15}H_{23}O$ [M^-] 219.1754; found 219.1752.

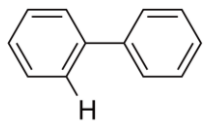

1,1'-biphenyl (2c) [CAS: 92-52-4]

General procedure was followed using 2-chloro-1,1'-biphenyl (47.2 mg, 0.25 mmol, 1 equiv.). After 24 h, the reaction was quenched and purified according to Purification A (5% EtOAc/Hexanes) affording the product as a white solid (35.1 mg, 90.6%, LED I; 36.0 mg, 93.3% yield, LED II). Characterization data of **2c** is consistent with literature reports.[6]

^{1}H NMR (400 MHz, $CDCl_3$) δ 7.64 – 7.58 (m, 4H), 7.49 – 7.42 (m, 4H), 7.40 – 7.33 (m, 2H).
^{13}C NMR (101 MHz, $CDCl_3$) δ 128.8, 127.2.

HRMS: calculated m/z for $C_{12}H_{11}$ $[M+H]^+$ 155.0866, found 155.0834.

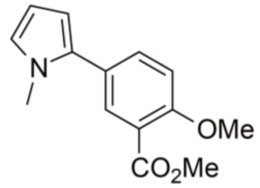

Methyl 2-methoxy-5-(1-methyl-1H-pyrrol-2-yl) benzoate (2p)[CAS: 2375910-20-4]

A tube equipped with a PTFE coated magnetic stirring bar was charged with methyl 5-chloro-2-methoxybenzoate (50.2 mg, 0.25 mmol, 1 equiv.), and then introduced into a glove box filled with nitrogen. There, 1.67 mL DMSO, TAEA (56 µL, 0.375 mmol, 1.5 equiv.) and heteroarene trapping agent (12.5 mmol, 50 equiv.) were sequentially added into the tube. Then 2.5 nmol QDs dispersed in hexane was added into the same tube. After stirring for several seconds, the mixture became homogeneous and clear. The tube was sealed to keep the N_2 atmosphere before removed from the glove box. The tube was put upon the stir plate equipped with blue LEDs and fans for cooling. Then the reaction was irradiated under LED II with 5 mW/cm^2 stirring (700 rpm) for 24 hours. The crude reaction mixture was subjected to Purification B and the resulting dry-loaded product was purified by column chromatography on silica (15% EtOAc/Hexanes) affording the product as a colorless oil (36.7 mg, 60% yield).
^{1}H NMR (400 MHz, $CDCl_3$) δ 7.83(d, J=2.4Hz,1H), 7.50 (dd, J=8.6,2.4 Hz, 1H), 7.02(d, J=8.6 Hz, 1H), 6.70(d, J=2.3 Hz,1H), 6.19(d, J=2.3 Hz, 2H), 3.94 (s,3H), 3.90 (s, 3H), 3.63(s, 3H).

^{13}C NMR (101 MHz, $CDCl_3$) δ166.6, 158.1, 133.7, 133.2, 132.0, 125.6, 123.4, 119.93, 112.1, 108.5, 107.7, 56.2, 52.1, 34.9.

HRMS: calculated m/z for $C_{14}H_{16}NO_3$ $[M+H]^+$ 246.1136; found 246.1131.

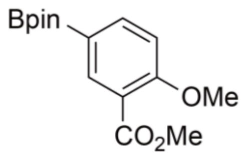

methyl 2-methoxy-5-(4,4,5,5-tetramethyl-1,3,2-dioxaborolan-2-yl) benzoate (2q) [CAS:478375-37-0]

A tube equipped with a PTFE coated magnetic stirring bar was charged with methyl 5-chloro-2-methoxybenzoate (50.2 mg, 0.25 mmol, 1 equiv.), and then introduced into a glove box filled with nitrogen. There, 1.25 mL DMSO, $NaCHO_2$ (51.0 mg, 0.75 mmol, 3 equiv.), bis(pinacolato)diboron (190.5 mg, 0.75 mmol, 3 equiv.), Cs_2CO_3 (244.4 mg, 0.75 mmol, 3 equiv.) were sequentially added into the tube. The mixture was stirred to dissolve reactants. Then 2.5 nmol QDs dispersed in hexane was added into the same tube. After stirring for several seconds, the mixture became homogeneous and clear. The tube was sealed to keep the N_2 atmosphere before removed from the glove box. The tube was put upon the stir plate equipped with blue LEDs and fans for cooling. Then the reaction was irradiated under LED II with 5 mW/cm^2 stirring (700 rpm) for 24 hours. The crude reaction mixture was subjected to Purification B and the resulting dry-loaded product was partially purified by column chromatography on silica (15% EtOAc/Hexanes). The crude reaction mixture was subjected to Purification B and the resulting dry-loaded product was purified by column chromatography on silica (15% EtOAc/Hexanes) affording the product as a colorless oil (36.5 mg, 50% yield). Characterization data of **2q** is consistent with literature reports.[6]

^{1}H NMR (400 MHz, $CDCl_3$) δ 8.21 (d, J = 1.7 Hz, 1H), 7.89 (dd, J = 8.3, 1.7 Hz, 1H), 6.95 (d, J= 8.4 Hz, 1H), 3.91 (s, 3H), 3.87 (s, 3H), 1.32 (s, 12H).

^{13}C NMR (101 MHz, $CDCl_3$) δ166.7, 161.5, 140.3, 138.4, 119.8, 111.3, 84.0, 56.0, 52.0, 24.9.

HRMS: calculated m/z for $C_{15}H_{22}BO_5$ $[M+H]^+$ 293.1560; found 293.1527.

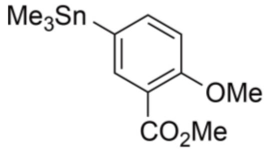

methyl 2-methoxy-5-(trimethylstannyl) benzoate (2r) [CAS: 151826-13-0]

A tube equipped with a PTFE coated magnetic stirring bar was charged with methyl 5-chloro-2-methoxybenzoate (50.2 mg, 0.25 mmol, 1 equiv.) then introduced into a glove box filled with nitrogen. There, 1 mL DMSO solvent $NaCHO_2$ (102.0 mg, 1.5 mmol, 6 equiv) and hexamethylditin (245.7 mg, 0.75 mmol, 3 equiv.) were

sequentially added into the tube. The mixture was stirred to dissolve reactants. Then 2.5 nmol QDs dispersed in hexane was added into the same tube. After stirring for several seconds, the mixture becomes homogeneous and clear. The tube was sealed to keep the N_2 atmosphere before removed from the glove box. The tube was put upon the stir plate equipped with blue LEDs and fans for cooling. Then the reaction is irradiated (under LED II with 5 mW/cm^2) with stirring (700 rpm) for 24 hours. The reaction mixture was partially purified according to Purification B (85% hexanes to 15% EtOAc), affording the product (2f) as a clear oil (32.6 mg, 37%).

^{1}H NMR (400 MHz, $CDCl_3$) δ 7.85 (m, *J* = 1.6 Hz, 1H), 7.65 – 7.47 (m, 1H), 6.97 (d, *J* = 8.1 Hz, 1H), 3.90 (s, 3H), 3.89 (s, 3H), 0.29 (s, 9H).
^{13}C NMR (101 MHz, $CDCl_3$) δ167.3, 159.4, 141.0, 138.6, 132.4, 120.2, 112.0, 56.0, 52.1, 9.3.

HRMS: calculated m/z for $C_{12}H_{18}O_3Sn$ [M+Na]$^+$ 353.0176; found 353.0178.

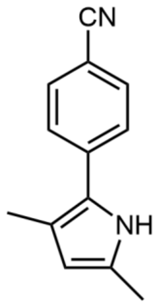

4-(3,5-Dimethyl-1*H*-pyrrol-2-yl)benzonitrile (2s) [CAS:2095284-38-9]

A tube equipped with a PTFE coated magnetic stirring bar was charged with 4-Chlorobenzonitrile (34.4 mg, 0.25 mmol, 1 equiv.), and then introduced into a glove box filled with nitrogen. There, 1.67 mL DMSO, TAEA (56 µL, 0.375 mmol, 1.5 equiv.) and heteroarene trapping agent 2,4-Dimethylpyrrole (12.5 mmol, 50 equiv.) were sequentially added into the tube. Then 2.5 nmol QDs dispersed in hexane was added into the same tube. After stirring for several seconds, the mixture became homogeneous and clear. The tube was sealed to keep the N_2 atmosphere before removed from the glove box. The tube was put upon the stir plate equipped with blue LEDs and fans for cooling. Then the reaction was irradiated under 5 mW/cm^2 blue LED stirring (700 rpm) for 24 hours. The crude reaction mixture was subjected to Purification B and the resulting dry-loaded product was purified by column chromatography on silica (15% EtOAc/Hexanes) affording the product as a brown oil (23.1 mg, 42% yield).

^{1}H NMR (400 MHz, $CDCl_3$) δ 8.05 (s, 1H), 7.62 (d, J = 8.4 Hz, 2H), 7.45 (d, J = 8.5 Hz, 2H), 5.87 (d, J = 2.8 Hz, 1H), 2.31 (s, 3H), 2.27 (s, 3H).

^{13}C NMR (101 MHz, $CDCl_3$) δ138.0, 132.5, 129.9, 125.0, 124.8, 119.9, 119.4, 111.6, 107.5, 13.0 (d, J = 11.4 Hz).

HRMS: calculated m/z for $C_{13}H_{13}N_2$ [M+H]$^+$ 197.1079; Found:197.1045.

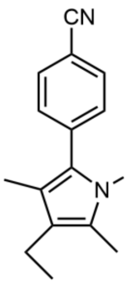

4-(3,5-Dimethyl-4-Ethyl-1*H*-pyrrol-2-yl)benzonitrile (2t)

A tube equipped with a PTFE coated magnetic stirring bar was charged with 4-Chlorobenzonitrile (34.4 mg, 0.25 mmol, 1 equiv.), and then introduced into a glove box filled with nitrogen. There, 1.67 mL DMSO, TAEA (56 µL, 0.375 mmol, 1.5 equiv.) and heteroarene trapping agent 3-Ethyl-2,4-dimethylpyrrole (7 mmol, 28 equiv.) were sequentially added into the tube. Then 2.5 nmol QDs dispersed in hexane was added into the same tube. After stirring for several seconds, the mixture became homogeneous and clear. The tube was sealed to keep the N_2 atmosphere before removed from the glove box. The tube was put upon the stir plate equipped with blue LEDs and fans for cooling. Then the reaction was irradiated under 5 mW/cm^2 blue LED stirring (700 rpm) for 24 hours. The crude reaction mixture was subjected to Purification B and the resulting dry-loaded product was purified by column chromatography on silica (15% EtOAc/Hexanes) affording the product as a brown oil (36.3 mg, 65% yield).

^{1}H NMR (400 MHz, $CDCl_3$) δ 7.93 – 7.76 (m, 1H), 7.64 (d, J = 8.4 Hz, 2H), 7.46 (d, J = 8.4 Hz, 2H), 2.48 (d, J = 7.6 Hz, 2H), 2.28 (s, 3H), 2.24 (s, 3H), 1.13 (t, J = 7.5 Hz, 3H).

^{13}C NMR (101 MHz, $CDCl_3$) δ138.1, 132.5, 126.2, 125.4, 124.1 (d, J = 35.1 Hz), 118.9 (d, J = 110.2 Hz), 107.6, 17.5, 15.5, 11.3, 10.8.

HRMS: calculated m/z for $C_{15}H_{17}N_2^+$ $[M+H]^+$ 225.1392; found 225.1360.

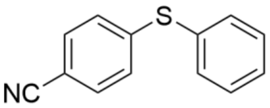

4-(Phenylthio)benzonitrile (2u) [CAS: 51238-46-1]

A tube equipped with a PTFE coated magnetic stirring bar was charged with 4-Chlorobenzonitrile (34.4 mg, 0.25 mmol, 1 equiv.), and then introduced into a glove box filled with nitrogen. There, 1.5 mL DMSO, TAEA (56 µL, 0.375 mmol, 1.5 equiv.) and heteroarene trapping agent diphenyl disulfide (0.75 mmol, 3 equiv.), K_2CO_3 (0.75 mmol, 3 equiv.) were sequentially added into the tube. Then 2.5 nmol QDs dispersed in hexane was added into the same tube. After stirring for several seconds, the mixture became homogeneous and clear. The tube was sealed to keep the N_2 atmosphere before removed from the glove box. The tube was put upon the stir plate equipped with blue LEDs and fans for cooling. Then the reaction was irradiated under 150 mW/cm^2 blue LED stirring (1000 rpm) for 20 hours. The crude reaction

mixture was subjected to Purification B and the resulting dry-loaded product was purified by column chromatography on silica (15% EtOAc/Hexanes) affording the product as a brown oil (45 mg, 85% yield).

^{1}H NMR (400 MHz, $CDCl_3$) δ 7.57 – 7.39 (m, 7H). 7.16 (d, J = 8.5 Hz, 2H).

^{13}C NMR (101 MHz, $CDCl_3$) δ 133.9, 131.8, 129.3, 128.8, 126.7.

HRMS: calculated m/z for $C_{13}H_{10}NS$ $[M+H]^+$ 212.0534; found 212.0532.

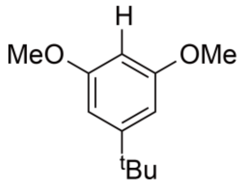

1-(tert-butyl)-3,5-dimethoxybenzene (4a) [CAS: 143029-45-2]

General procedure A was followed using 4-(tert-butyl)-2,6-dimethoxyphenyl diethyl phosphate (86.6 mg, 0.25 mmol, 1 equiv.). DIPEA (174 µL, 1 mmol, 4 equiv.) instead of TAEA was used as the sacrificial agent. After 18h (under LED I with 150 mW/cm^2) or 55 h (under LED II with 5mW/cm^2), the reaction was quenched and purified according to Purification A (85% hexanes to 15% EtOAc), affording the product (**4**) as a white solid (38 mg, 78.4%, LED I; 26.8 mg, 55 % yield, LED II). Characterization data of **4** is consistent with literature reports.[6]

^{1}H NMR (400 MHz, $CDCl_3$) δ 6.55 (d, J = 2.3 Hz, 2H), 6.31 (t, J = 2.3 Hz, 1H), 3.80 (s, 6H), 1.30 (s, 9H).
^{13}C NMR (101 MHz, $CDCl_3$) δ 160.6, 154.0, 104.2, 96.9, 55.3, 35.1, 31.4.

HRMS: calculated m/z for $C_{12}H_{19}O_2$ $[M+H]^+$ 195.1385; found 195.1320.

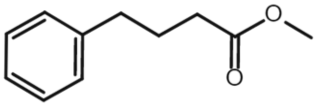

methyl 4-phenylbutanoate (6) [CAS: 2046-17-5]

A tube equipped with a PTFE coated magnetic stirring bar was charged with methyl 2-phenylcyclopropane-1-carboxylate (44.1 mg, 0.25 mmol, 1 equiv.), and then introduced into a glove box filled with nitrogen. There, 1 mL DMPU solvent and 56 µL TAEA (0.375 mmol, 1.5 equiv.) were sequentially added into the tube. The mixture was stirred for a while to dissolve reactants. Then 2.5 nmol QDs dispersed in hexane was added into the same tube. After stirring for several seconds, the mixture becomes homogeneous and clear. The tube was sealed to keep the N_2 atmosphere before removed from the glove box. The tube was put upon the stir plate equipped with blue LEDs and fans for cooling. Then the reaction was irradiated with stirring (700 rpm) for 24h (under LED I with 150 mW/cm^2) or 55 h (under LED II with

5mW/cm^2). After the reaction was complete, the reaction mixture was purified according to Purification B (15% EtOAc/hexanes), affording the product and as a clear oil (36.1 mg, 81% yield, LED I; 18.1 mg, 40% yield, LED II). Characterization data of **6** is consistent with literature reports.[6]

^{1}H NMR (400 MHz, $CDCl_3$) δ 7.32 – 7.26 (m, 2H), 7.23 – 7.15 (m, 3H), 3.67 (s, 3H), 2.68 – 2.63 (m, 2H), 2.34 (t, J = 7.5 Hz, 2H), 1.97 (p, J = 7.6 Hz, 2H).
^{13}C NMR (101 MHz, $CDCl_3$) δ174.1, 141.5, 128.5 (d, J = 14.8 Hz), 126.1, 51.6, 35.21, 33.5, 26.6.

HRMS: calculated m/z for $C_{11}H_{15}O_2$ [M+H]$^+$ 179.1072; found 179.1042.

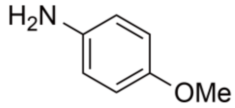

4-methoxyaniline (10b) [CAS: 104-94-9]

General procedure was followed using N-(4-methoxyphenyl)-4-methylbenzenesulfonamide (138.7 mg, 0.5 mmol, 1 equiv.) with 5 nmol QDs. After 18 h (under LED I with 150 mW/cm^2) or 24 h (under LED II with 5mW/cm^2), the reaction was quenched and purified according to Purification A (50% EtOAc/Hexanes) affording the product as a white solid (55.7 mg, 90.5% yield, LED I; 56.4 mg, 91.3% yield, LED II). Characterization data of **6** is consistent with literature reports.[6]

^{1}H NMR (400 MHz, $CDCl_3$) δ 6.77 – 6.72 (m, 2H), 6.69 – 6.62 (m, 2H), 3.75 (s, 3H), 3.41 (br, 2H).
^{13}C NMR (101 MHz, $CDCl_3$) δ152.9, 140.0, 116.5, 114.9, 55.8.

HRMS: calculated m/z for $C_7H_{10}NO$ [M+H]$^+$ 124.0762; found 124.0752.

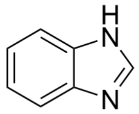

Benzimidazole (10e) [CAS: 51-17-2]

General procedure was followed using 1-[(4-Methylphenyl)sulfonyl]-1H-benzimidazole (71.8mg, 0.25 mmol, 1 equiv.). After 20 h (under LED I with 150 mW/cm^2), the reaction was quenched and purified according to Purification A (75% EtOAc/Hexanes) affording the product as a white solid (28.3 mg, 96% yield). Characterization data of 6e is consistent with literature reports.

^{1}H NMR (400 MHz, $CDCl_3$) δ 8.14 (s, 1H), 7.70 (dd, J = 6.0, 3.2 Hz, 2H), 7.33 (dd, J = 6.1, 3.1 Hz, 2H), 4.45 (s, 1H).

^{13}C NMR (101 MHz, $CDCl_3$) δ 140.4, 137.3, 123.1, 115.5.

HRMS: calculated m/z for $C_7H_6N_2^+$ [M+H]$^+$ 119.0609; found 119.0626.

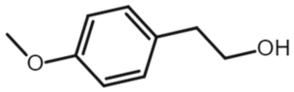

2-(4-methoxyphenyl)ethan-1-ol (12) [CAS: 702-23-8]

A tube equipped with a PTFE coated magnetic stirring bar was charged with 1-(2-(benzyloxy)ethyl)-4-methoxybenzene (60.6 mg, 0.25 mmol, 1 equiv.), and then introduced into a glove box filled with nitrogen. There, 1 mL DMPU solvent and 56 μL TAEA (0.375 mmol, 1.5 equiv.) were sequentially added into the tube. The mixture was stirred to dissolve reactants. Then 2.5 nmol QDs dispersed in hexane was added into the same tube. After stirring for several seconds, the mixture becomes homogeneous and clear. The tube was sealed to keep the N_2 atmosphere before being removed from the glove box. The tube was put upon the stir plate equipped with blue LEDs and fans for cooling. Then the reaction was irradiated stirring (700 rpm) for 24h (under LED I with 150 mW/cm^2) or 55 h (under LED II with 5mW/cm^2). The reaction mixture was quenched and purified according to Purification A (50% EtOAc/hexanes), affording the product as a clear oil (32.8 mg, 86.2% yield, LED I; 32.0 mg, 81.4% yield, LED II). Characterization data of **10** is consistent with literature reports.[6]

^{1}H NMR (400 MHz, $CDCl_3$) δ 7.18 – 7.12 (m, 2H), 6.90 – 6.83 (m, 2H), 3.83 (t, J = 6.6 Hz, 2H), 3.80 (s, 3H), 2.82 (t, J = 6.5 Hz, 2H), 1.36 (br, 1H).

^{13}C NMR (101 MHz, $CDCl_3$) δ158.4, 130.5, 130.1, 114.1, 63.9, 55.4, 38.4.

HRMS: calculated m/z for $C_9H_{13}O_2$ $[M+H]^+$ 153.0916; found 153.0923.

VIII. Sequential Substitution Reaction Procedures

Step 1 Procedures

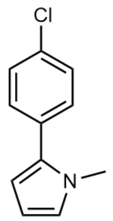

1H-Pyrrole, 2-(4-chlorophenyl)-1-methyl- (16) [CAS: 136146-68-4]

A tube equipped with a PTFE coated magnetic stirring bar was charged with 1-bromo-4-chlorobenzene (47.8 mg, 0.25 mmol, 1 equiv.), and then introduced into a glove box filled with nitrogen. There, 1.67 mL DMSO, TAEA (56 µL, 0.375 mmol, 1.5 equiv.) and heteroarene trapping agent N-methylpyrrole (4.5 mmol, 18.0 equiv.) were sequentially added into the tube. Then 2.5 nmol QDs dispersed in hexane was added into the same tube. After stirring for several seconds, the mixture becomes homogeneous and clear. The tube was sealed to keep the N_2 atmosphere before removed from the glove box. The tube was put upon the stir plate equipped with blue LEDs and fans for cooling. Then the reaction was irradiated with stirring (700 rpm) for 48 hours. The crude reaction mixture was subjected to Purification B and the

resulting dry-loaded product was partially purified by Preparative Thin-Layer Chromatography (10% EtOAc/Hexanes), affording the product as a light-yellow oil (26 mg, 54% yield). Characterization data of **12** is consistent with literature reports[8].

^{1}H NMR (400 MHz, $CDCl_3$) δ 7.34 (d, J = 8.2 Hz, 4H), 6.72 (s, 1H), 6.27 – 6.15 (m, 2H), 3.65 (s, 3H).

^{13}C NMR (101 MHz, $CDCl_3$) δ133.4, 132.7, 131.8, 129.8, 128.6, 124.1, 109.0, 107.96, 35.1.

HRMS: calculated m/z for $C_{11}H_{11}ClN^{+}$ $[M+H]^{+}$ 192.0580; found 192.0583.

Step 2 Procedures

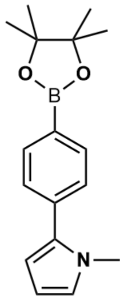

1-Methyl-2-[4-(4,4,5,5-tetramethyl-1,3,2-dioxaborolan-2-yl)phenyl]-1H-pyrrole (17) [CAS: 2099672-44-1]

A tube equipped with a PTFE coated magnetic stirring bar was charged with compound 12 (47.8 mg, 0.25 mmol, 1 equiv.), and then introduced into a glove box filled with nitrogen. There, 1.25 mL DMSO, $NaCHO_2$ (51.0 mg, 0.75 mmol, 3 equiv.), bis(pinacolato)diboron (190.5 mg, 0.75 mmol, 3 equiv.), K_2CO_3 (103.5 mg, 0.75 mmol, 3 equiv.), 4-cyanopyridine (4-CN-Py, 2.6 mg, 0.1 equiv.) were sequentially added into the tube. Then 2.5 nmol QDs dispersed in hexane was added into the same tube, stirring for 5 min. The tube was sealed to keep the N_2 atmosphere before removed from the glove box. The tube was put upon the stir plate equipped with blue LEDs and fans for cooling. Then the reaction was irradiated with stirring (700 rpm) for 24h (under LED I with 150 mW/cm^2). The crude reaction mixture was subjected to Purification B and the resulting dry-loaded product was partially purified by preparative thin-layer chromatography (20% EtOAc/Hexanes), affording the product as a white solid (32.6 mg, 46% yield).

^{1}H NMR (400 MHz, DMSO-d6) δ 7.67(s, 1H), 7.60-7.56 (t, J=8.3 Hz, 2H), 7.46-7.42(t, J=7.6 Hz, 1H), 6.84(m, 1H), 6.15(br, 1H), 6.07(br, 1H), 3.62 (s, 3H).

^{13}C NMR (151 MHz, DMSO-d_6) δ 134.4, 133.7, 133.0, 132.9, 131.4, 128.5, 124.8, 108.9, 107.9, 84.2, 55.4, 35.3, 31.2, 25.1.

HRMS: calculated m/z for $C_{17}H_{22}BNO_2$ 283.1744 $[M+H]^{+}$; found 283.1762.

IX. Characterization of QDs: PXRD, UV-vis absorption, TEM, EDX, TRPL, and ICP data

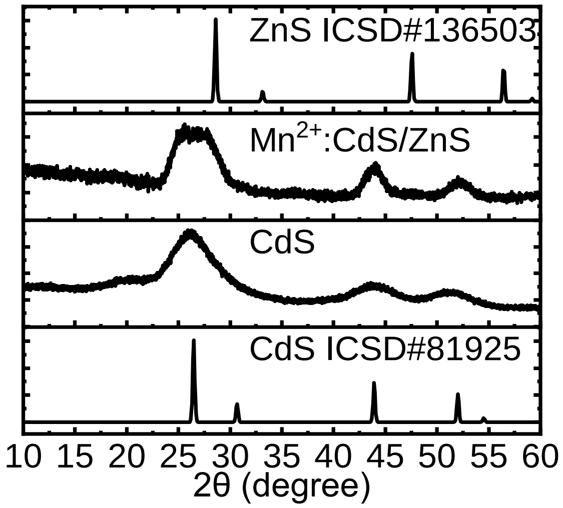

Figure S1. PXRD patterns of as synthesized CdS core and $Mn^{2+}{}_{C}$:CdS/ZnS QDs.

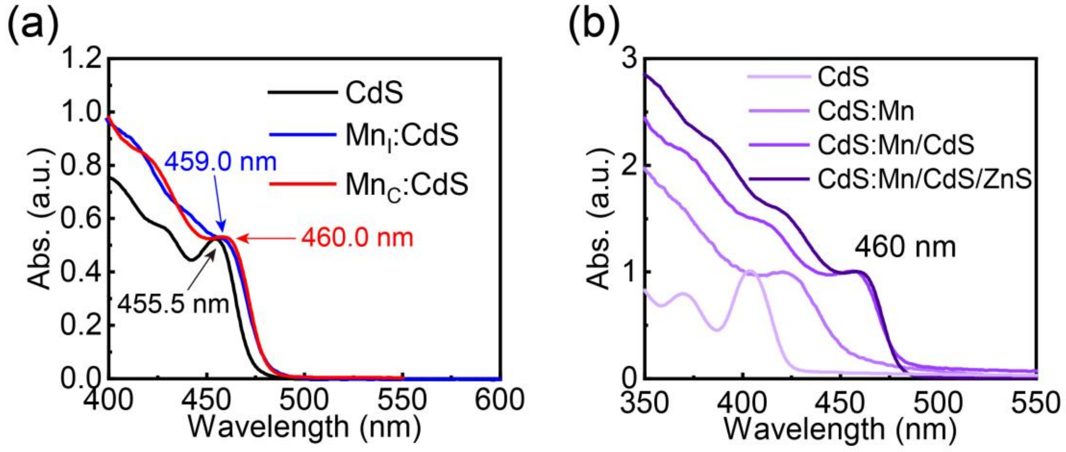

Figure S2. (a) Absorption spectra evolution of QDs in different fabrication stages; (b) Absorption spectra of CdS/ZnS, Mn_I:CdS/ZnS and Mn_C:CdS quantum dots.

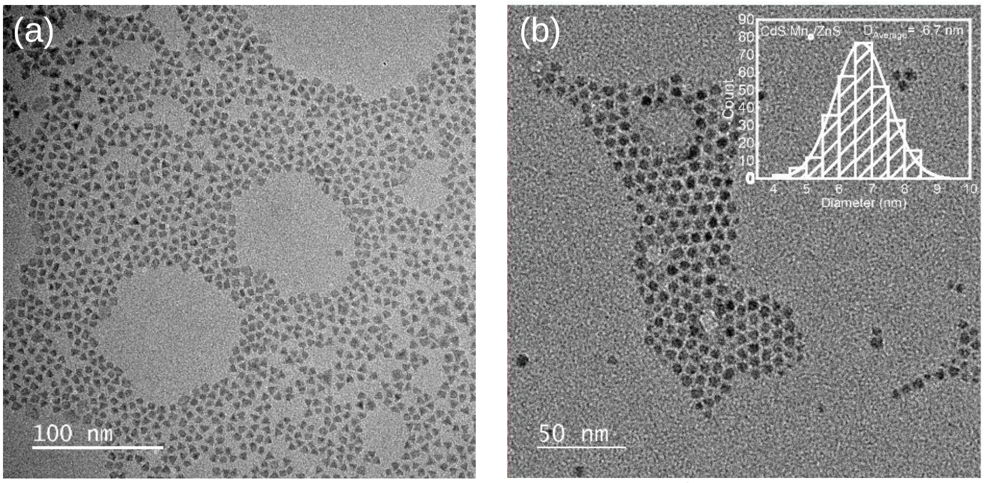

Figure S3. TEM images of (a) CdS core; (b) $Mn^{2+}{}_{C}$: CdS/ZnS QDs

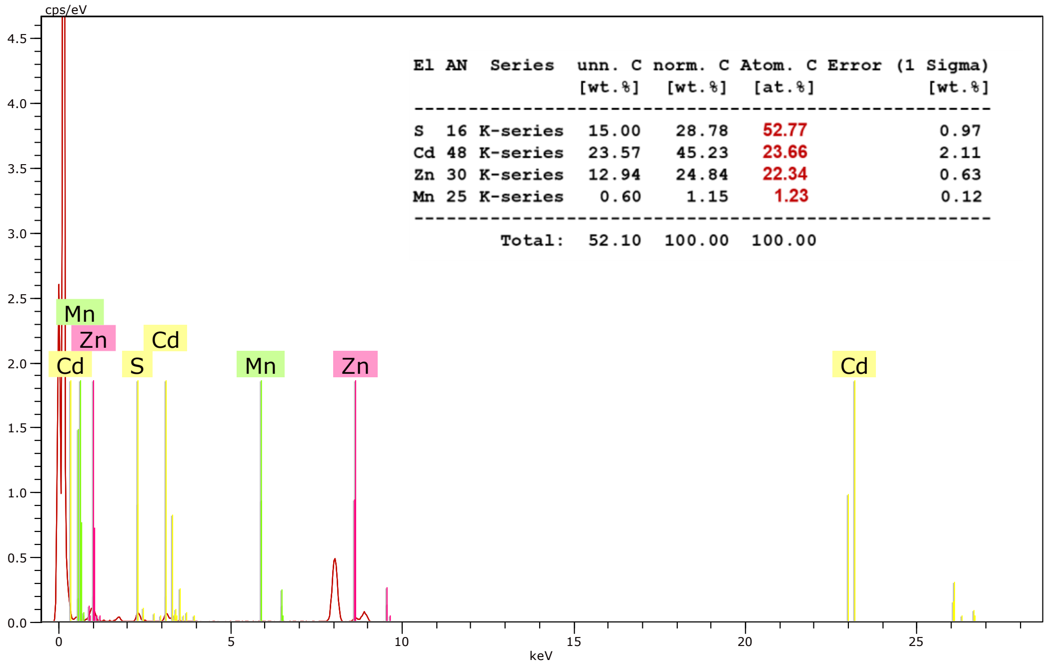

Figure S4. EDS result of as synthesized $Mn^{2}{}_{C}{}^{+}$: CdS/ZnS QDs and concentration of each element.

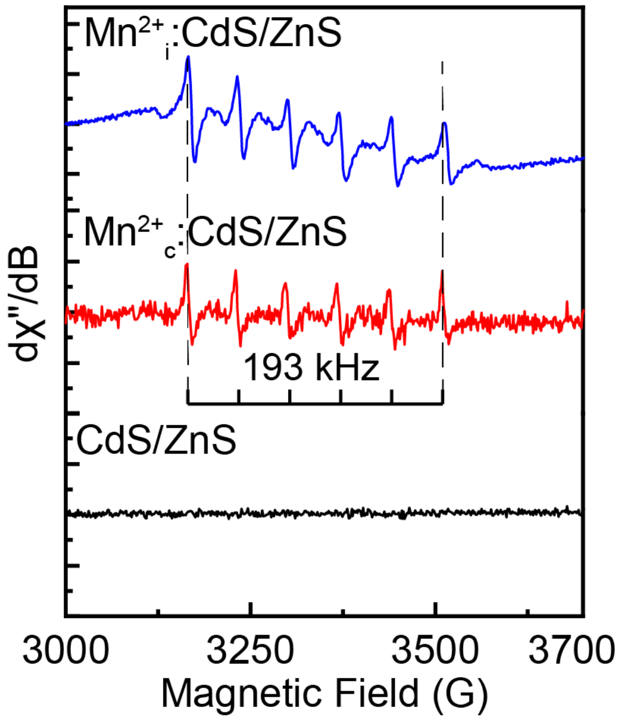

Figure. S5. Electron paramagnetic resonance (EPR) spectra of undoped and Mn^{2+}-doped CdS/ZnS QDs.

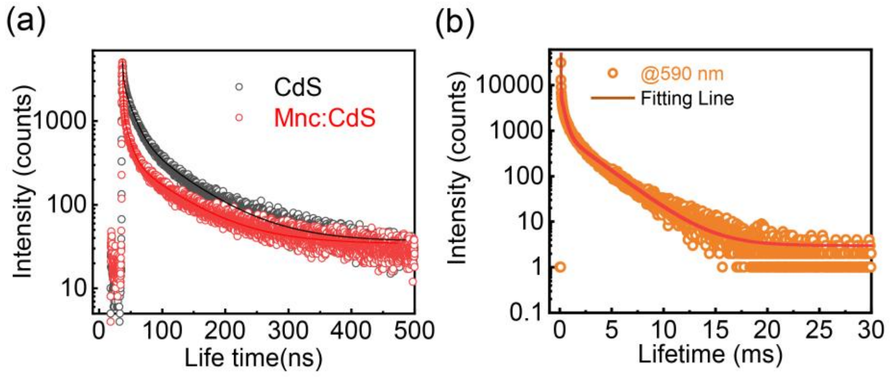

Figure S6. (a)Decay curve and fitting line of CdS QDs (black curve) and $Mn^{2+}{}_{C}$:CdS/ZnS QDs (red curve) at 465 nm and (b) Mn emission decay curve of $Mn^{2+}{}_{C}$:CdS/ZnS QDs @590 nm.

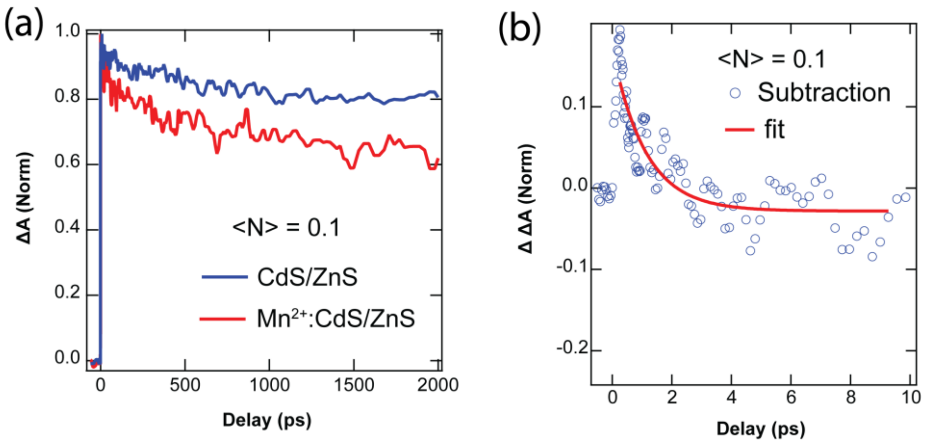

Figure S7. (a) 1S bleach kinetics of undoped and Mn^{2+}-doped CdS/ZnS QDs at excitation of <N> =0.1. (b) Subtracted TA kinetics and the monoexponential fit, yielding τ_{ET} ~ 3 ps.

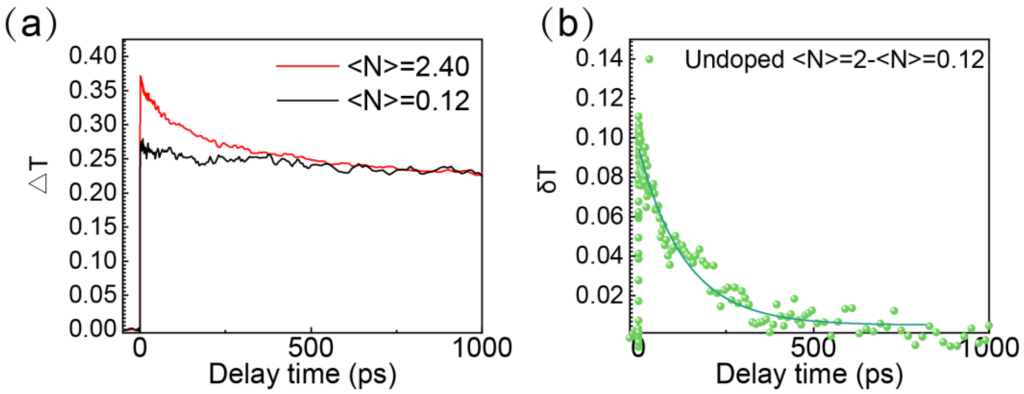

Figure S8. (a) The 1S TA dynamics of the undoped CdS QDs obtained using 3.35 eV excitation and two different pump fluences, ⟨N⟩ = 0.12 (black) and 2.4 (red). (b) The extracted Auger dynamics obtained by subtracting tail-normalized high- and low-pump-fluence traces (δT is the difference between TA signals for the different ⟨N⟩ values).

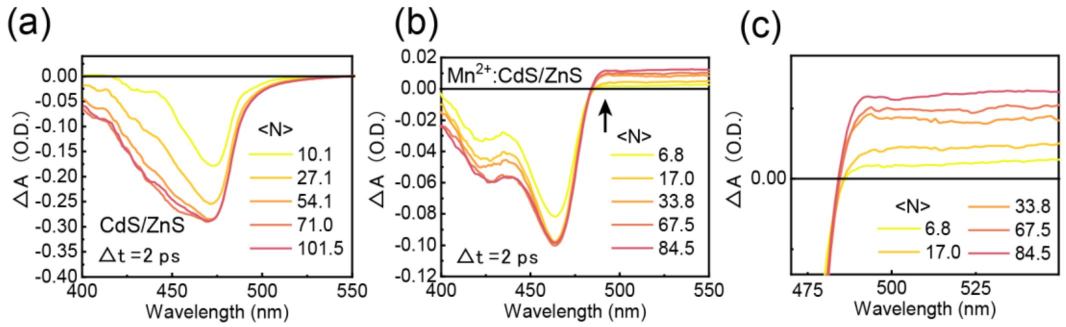

Figure. S9. TA spectra of CdS/ZnS (a) and Mn^{2+}-CdS/ZnS QDs (b) at different excitation rates. (c) Zoom-in spectra of the PIA signal in Mn^{2+}-CdS/ZnS QDs at different excitation rates.

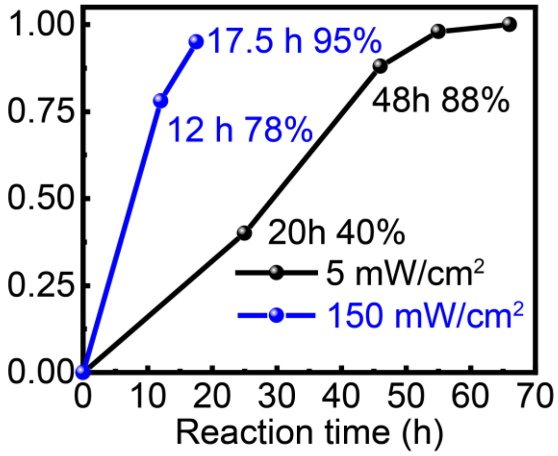

Figure S10. Model reaction procedure under different irradiation power monitored by NMR.

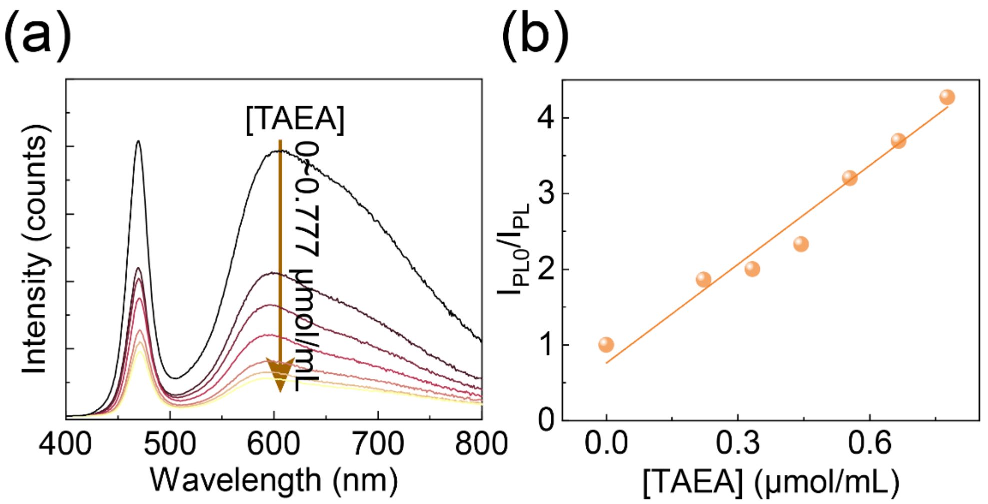

Figure. S11. (a) PL spectra of Mn^{2+}:CdS/ZnS QDs with various concentrations of TAEA molecules (0–1.55 μmol). (b) I_{PL0}/I_{PL} vs [TAEA]. I_{PL0} represents the PL intensity of QDs without TAEA.

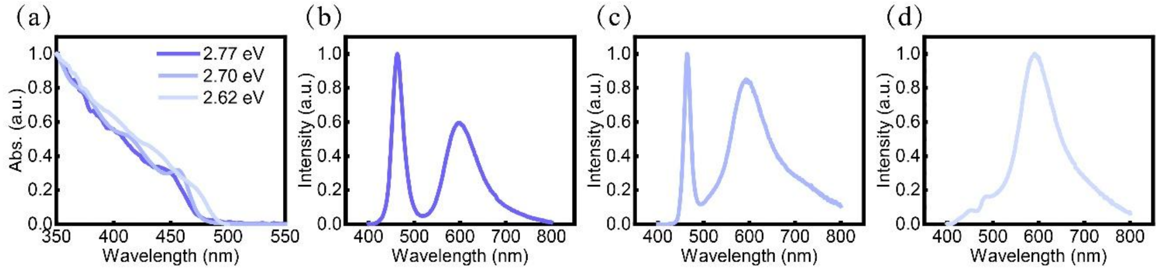

Figure S12. (a) Size dependent absorption spectra; (b) PL spectrum of Mn^{2+}:CdS/ZnS QDs with bandgap of 2.77 eV; (c) PL spectrum of Mn^{2+}:CdS/ZnS QDs with bandgap of 2.70 eV; (d) PL spectrum of Mn^{2+}:CdS/ZnS QDs with bandgap of 2.62 eV.

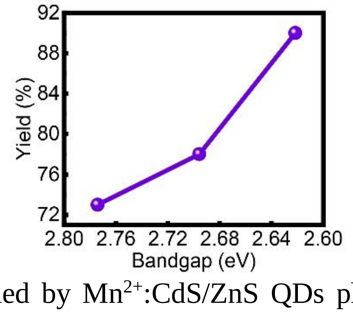

Figure S13. Reaction yields afforded by Mn^{2+}:CdS/ZnS QDs photocatalysts with different size.

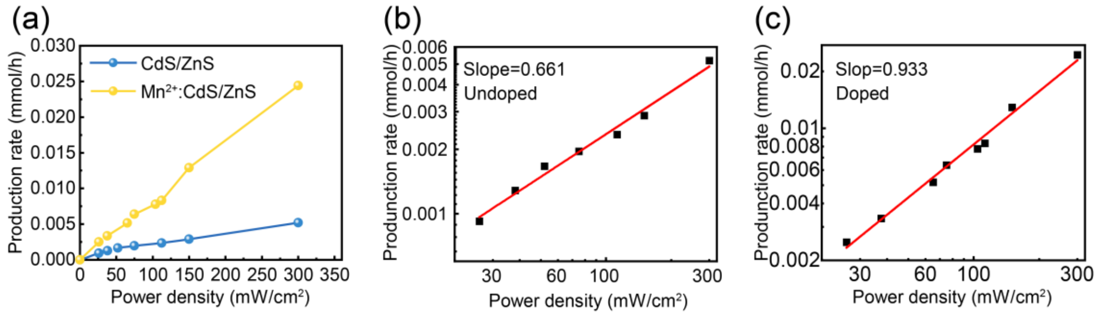

Figure. S14 Reaction rate as a function of irradiation intensity for undoped and doped CdS/ZnS QDs.

Table S2. ICP result of as prepared $Mn^{2+}{}_C$:CdS/ZnS Quantum dots.

**Mn (ppm)**	**Cd (ppm)**	**Zn (ppm)**
0.275	43.8	38.1

Table S3. PL Decay time fitting results of $Mn^{2+}{}_C$:CdS/ZnS quantum dots decay curves at 590 nm and 465 nm.

	τ_1	τ_2	τ_3
@590 nm	0.01 ms	0.33 ms	2.57 ms
CdS@465 nm	1.68 ns	14.07 ns	71.57 ns
Mnc:CdS@465 nm	1.04 ns	9.67 ns	71.53 ns

Table S4 TA kinetics fitting result of CdS, Mn^{2+}_C:CdS/ZnS, Mn^{2+}_I:CdS/ZnS quantum dots at respective photobleaching position.

	**CdS**		**CdS-Mn-core**		**CdS-Mn-inter**	
	Coef.	Sig.	Coef.	Sig.	Coef.	Sig.
ΔA_{Mn}	0	0	0.002988	0.0001683	0.001951	0.0001664
ΔA_{trap}	0.00083	0.0001177	0.002373	0.000175	0.00098	0.002232
ΔA_{re}	0.01398	5.63e-05	0.01000	0.000165	0.011989	0.0001205
t_{Mn}/ps	0	0	186.0	29.6	97.2	20.6
t_{trap}/ps	8.9	1.4	8.9	1.4	8.9	1.4
t_{re}/ps	8349.8	800.3	8349.8	800.3	8349.8	800.3

X. ^{1}H NMR data

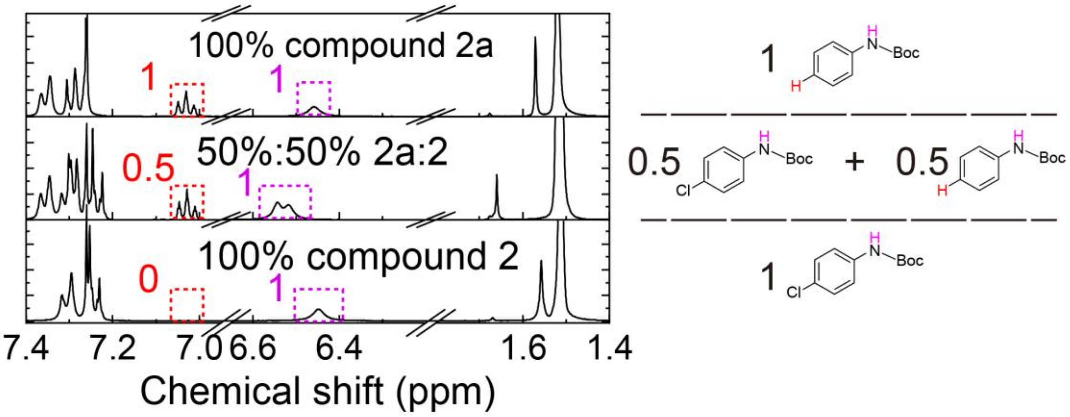

Figure S15. The NMR spectra of pure compound 2, the mixture of compound 2 and 2a with 1:1 ratio, and pure compound 2a (from bottom to top). The resonance at round 7.03 ppm is the signal from the hydrogen of compound 2a (marked with red) which substitutes the Cl in compound 2, and the resonance at around 6.4-6.5 is the signal from the H connected with N (marked with purple). Since there is both 1 H signal appear at 6.5-6.4 ppm in 2 and 2a NMR spectra, we set its integration to 1. No matter what the ratio of 2a to 2 is, the integration of purple H is always kept the same if the total mole quantity of 2a and 2 doesn't change. While the integration of red H will be larger with the increase of 2a linearly. Therefore, we can calculate the percentage of compound 2a and 2 according to the integration ratio of red H to purple H.

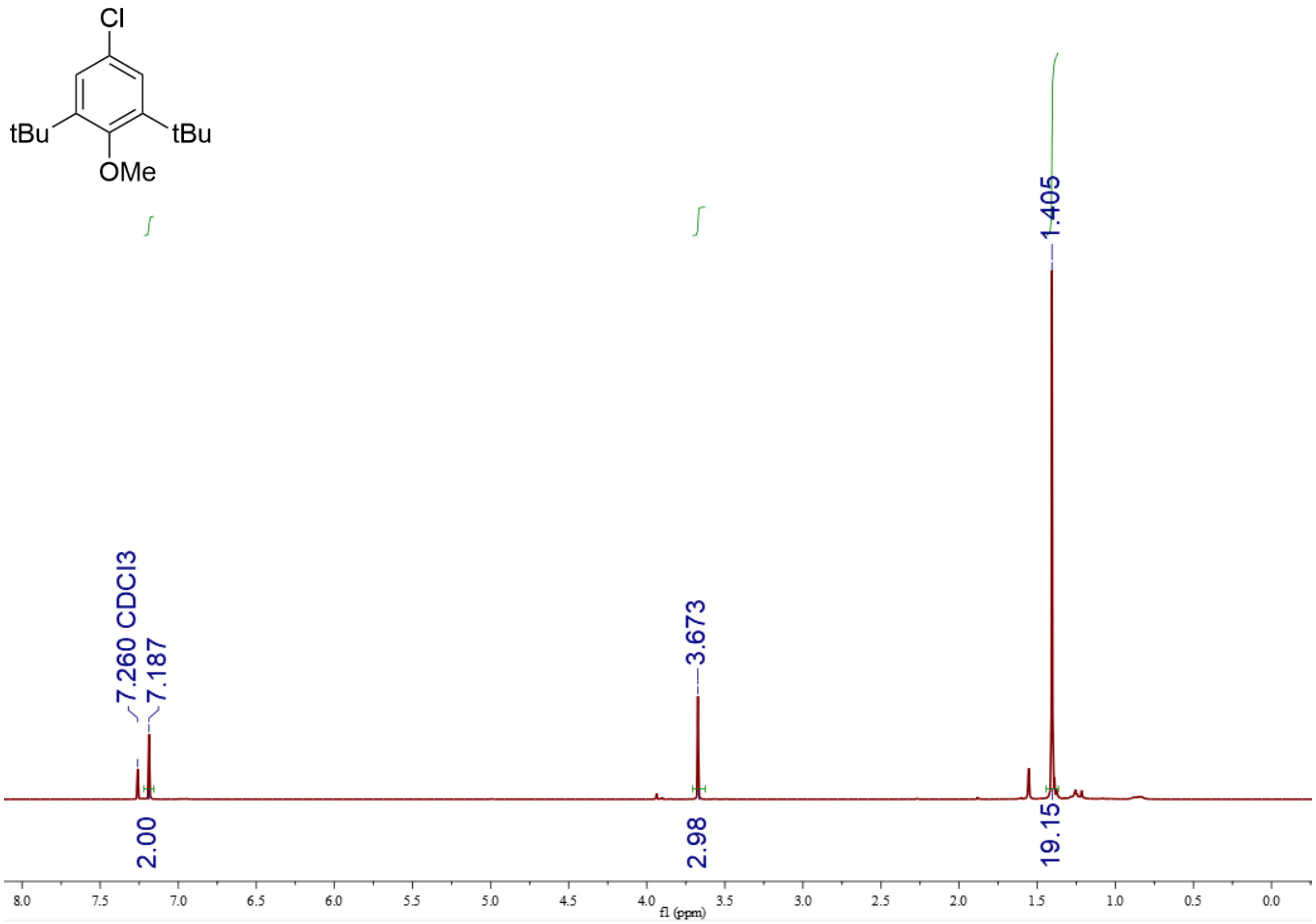

Figure S16. ^{1}H NMR spectrum of compound **1b** (400 MHz, $CDCl_3$)

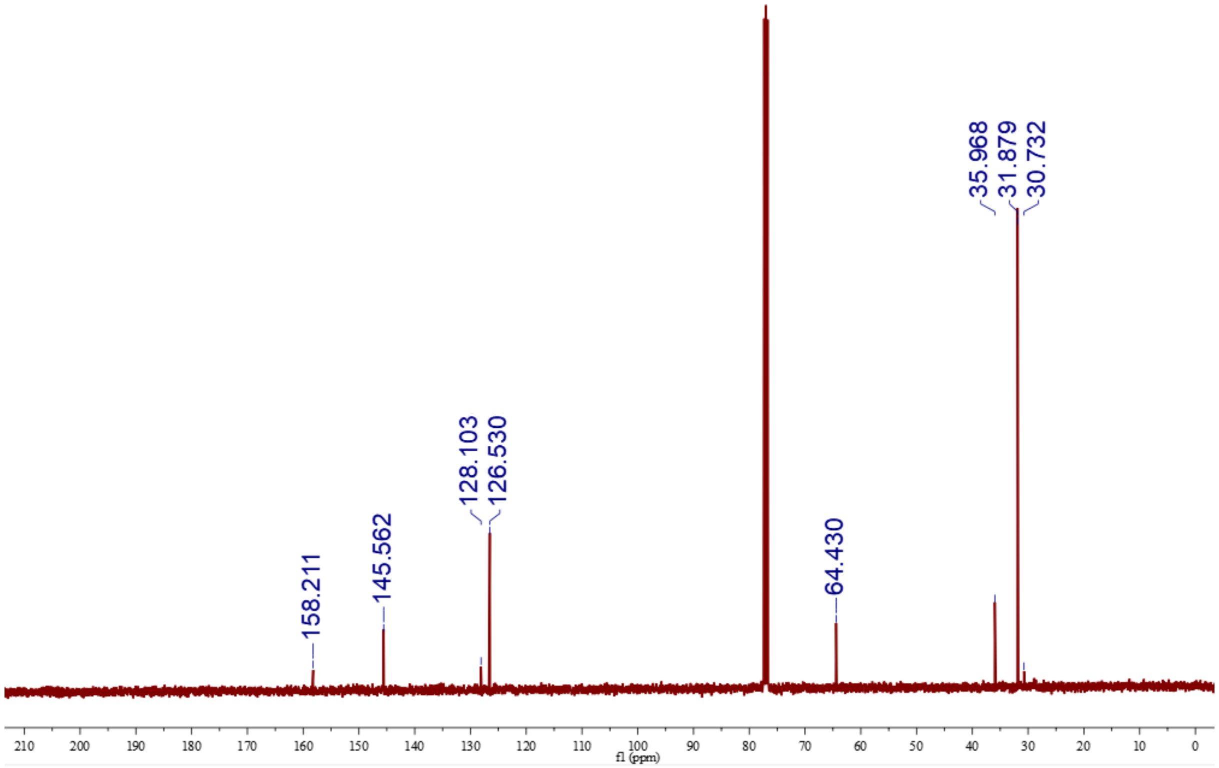

Figure S17. ^{13}C NMR spectrum of compound **1b** (101 MHz, $CDCl_3$)

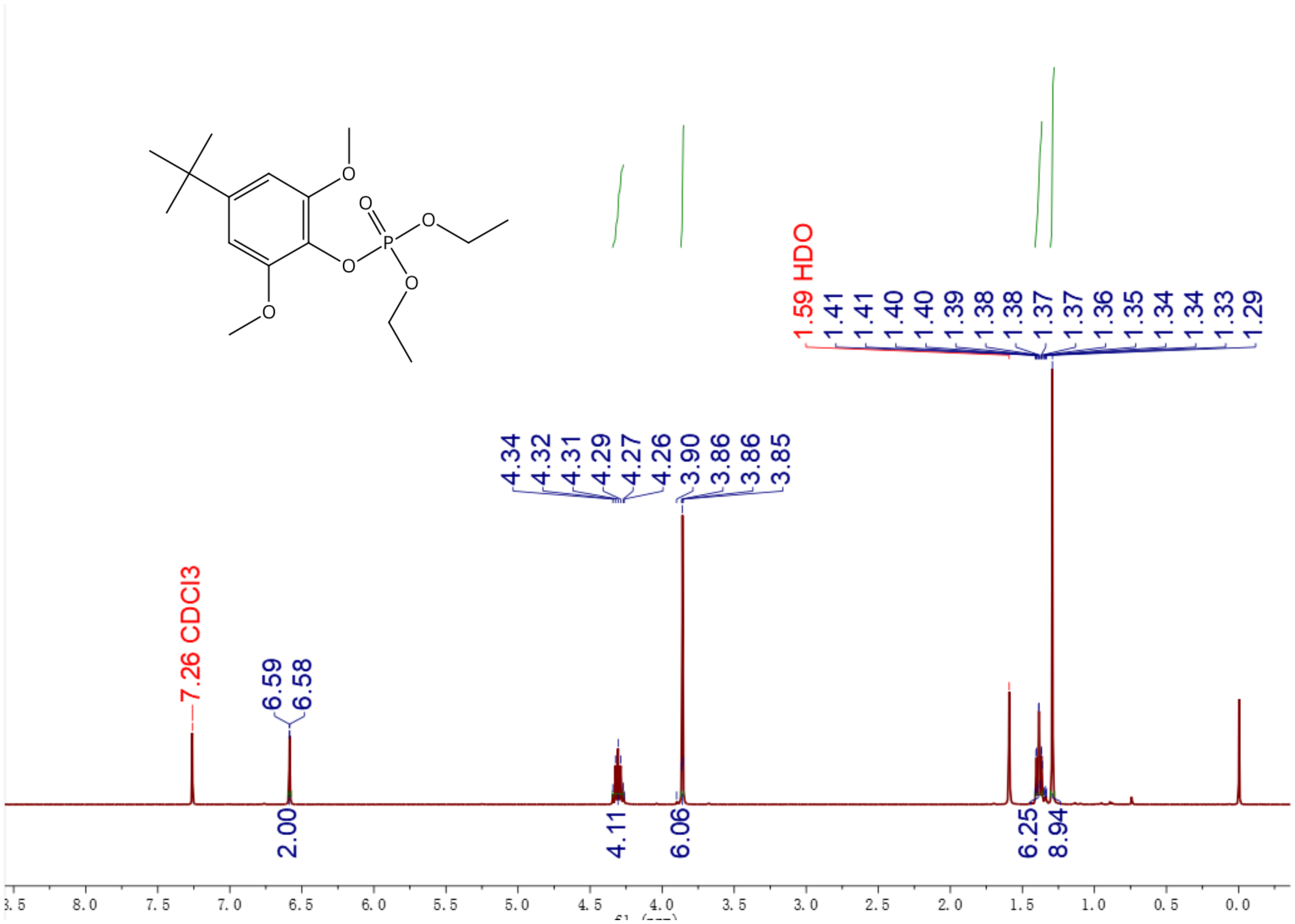

Figure S18. ^{1}H NMR spectrum of compound **3a** (400 MHz, $CDCl_3$)

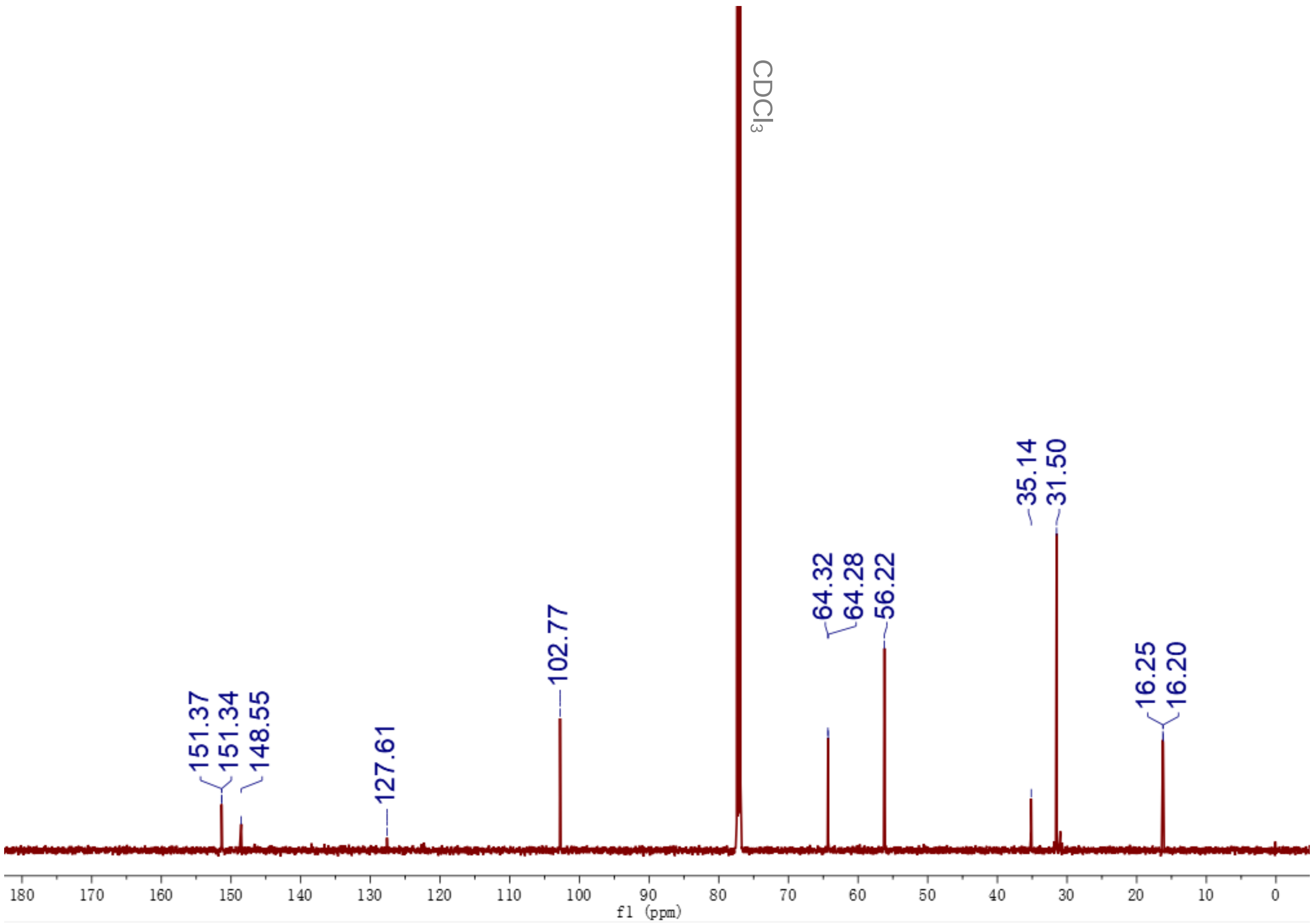

Figure S19. ^{13}C NMR spectrum of compound **3a** (101 MHz, $CDCl_3$)

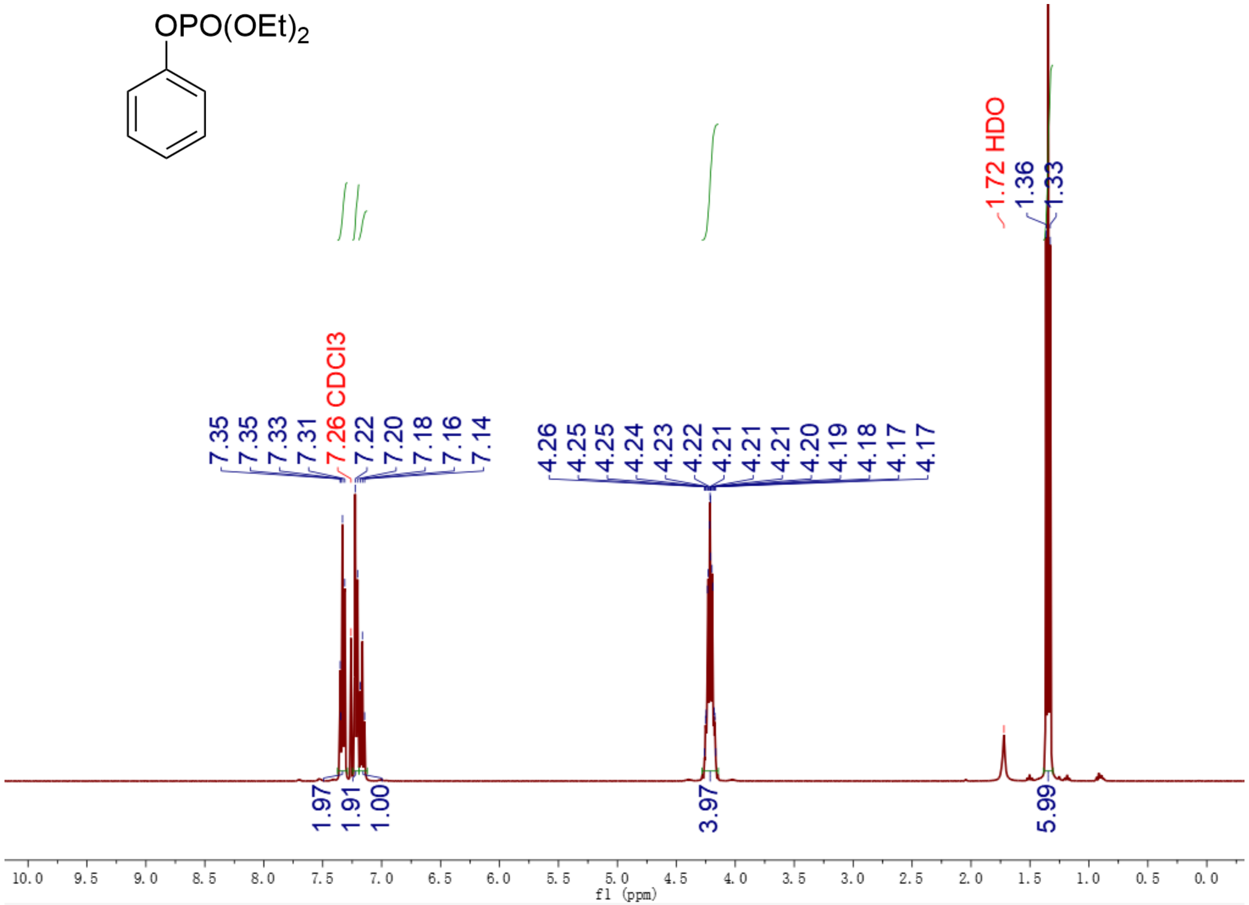

Figure S20. ^{1}H NMR spectrum of compound **3b** (400 MHz, $CDCl_3$)

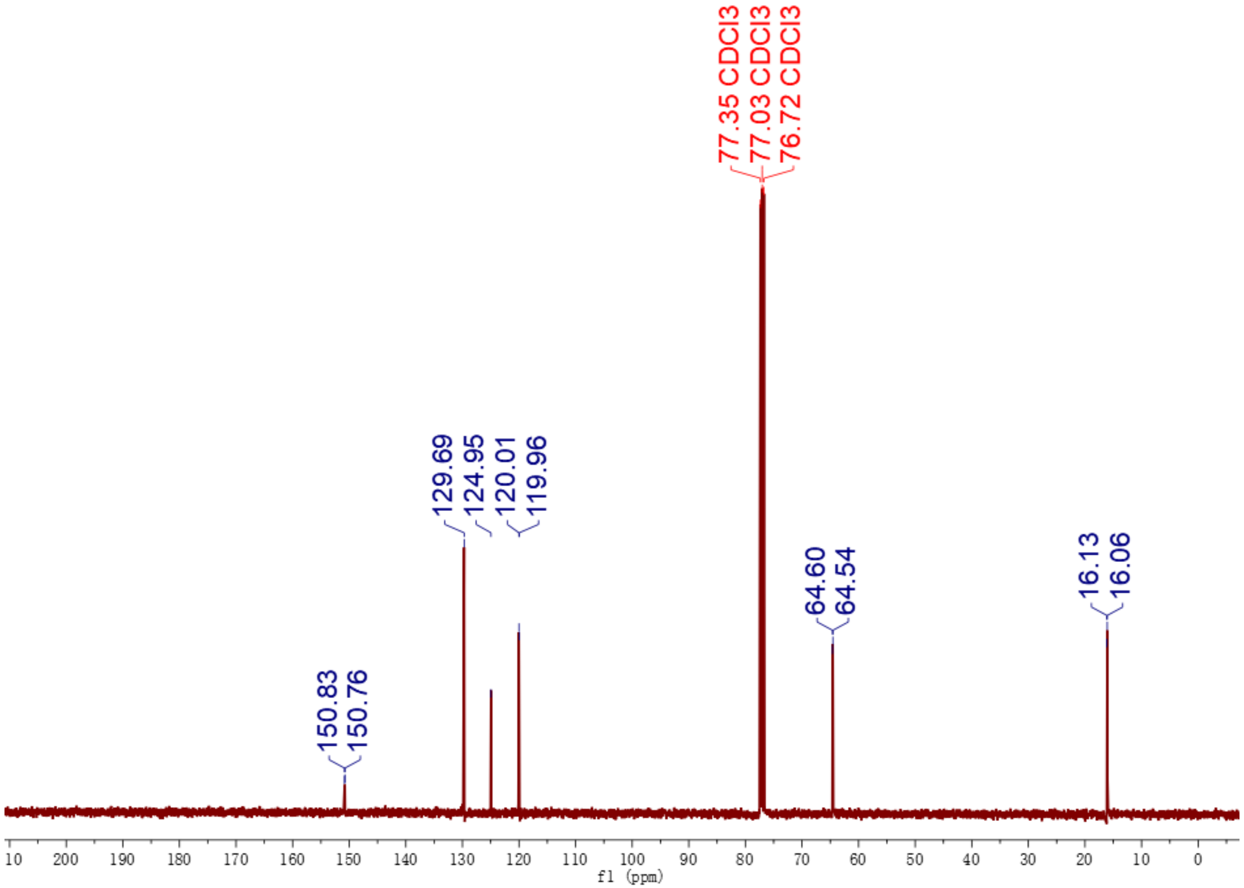

Figure S21. ^{13}C NMR spectrum of compound **3b** (101 MHz, $CDCl_3$)

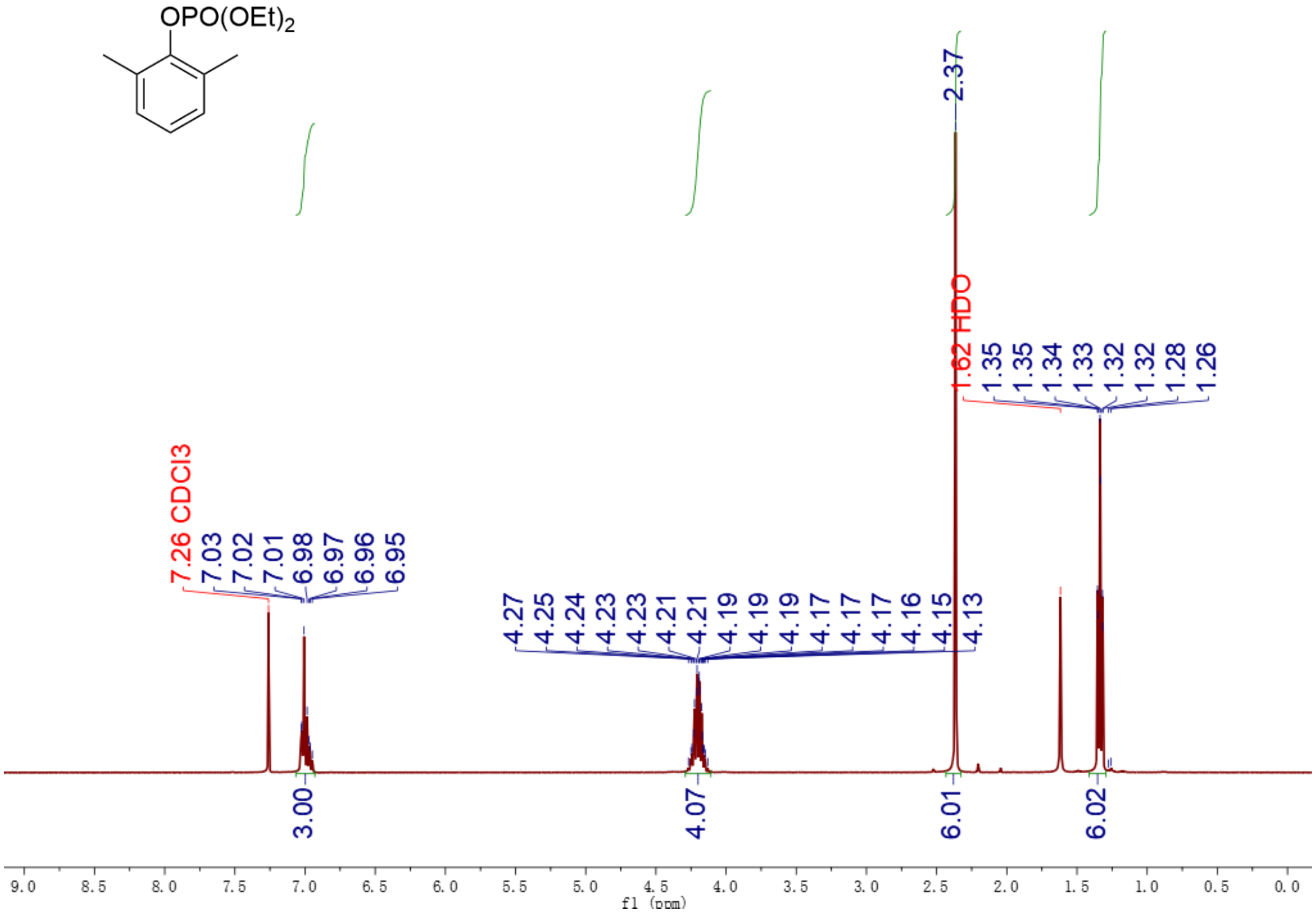

Figure S22. ^{1}H NMR spectrum of compound **3c** (400 MHz, $CDCl_3$)

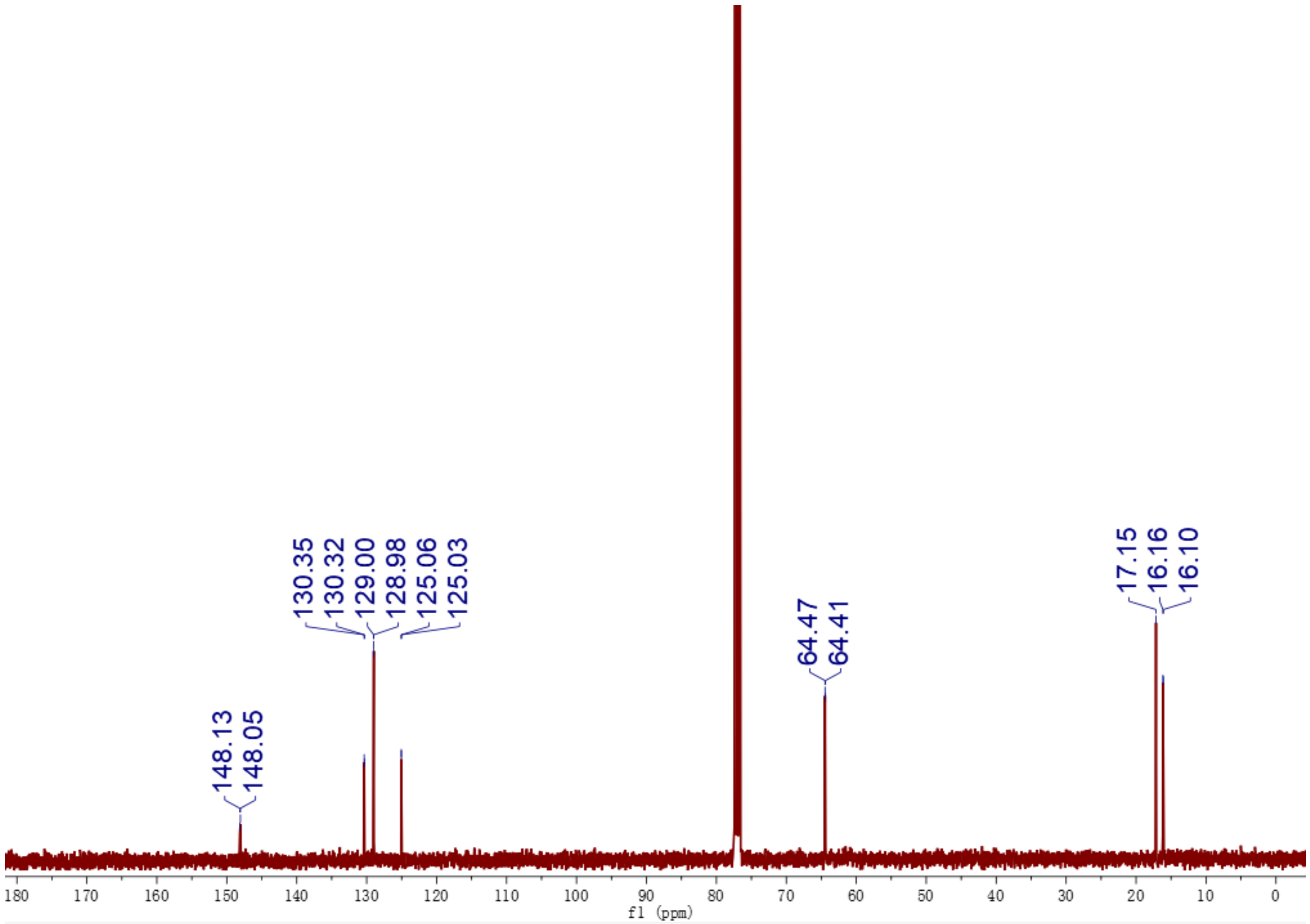

Figure S23. ^{13}C NMR spectrum of compound **3c** (101 MHz, $CDCl_3$)

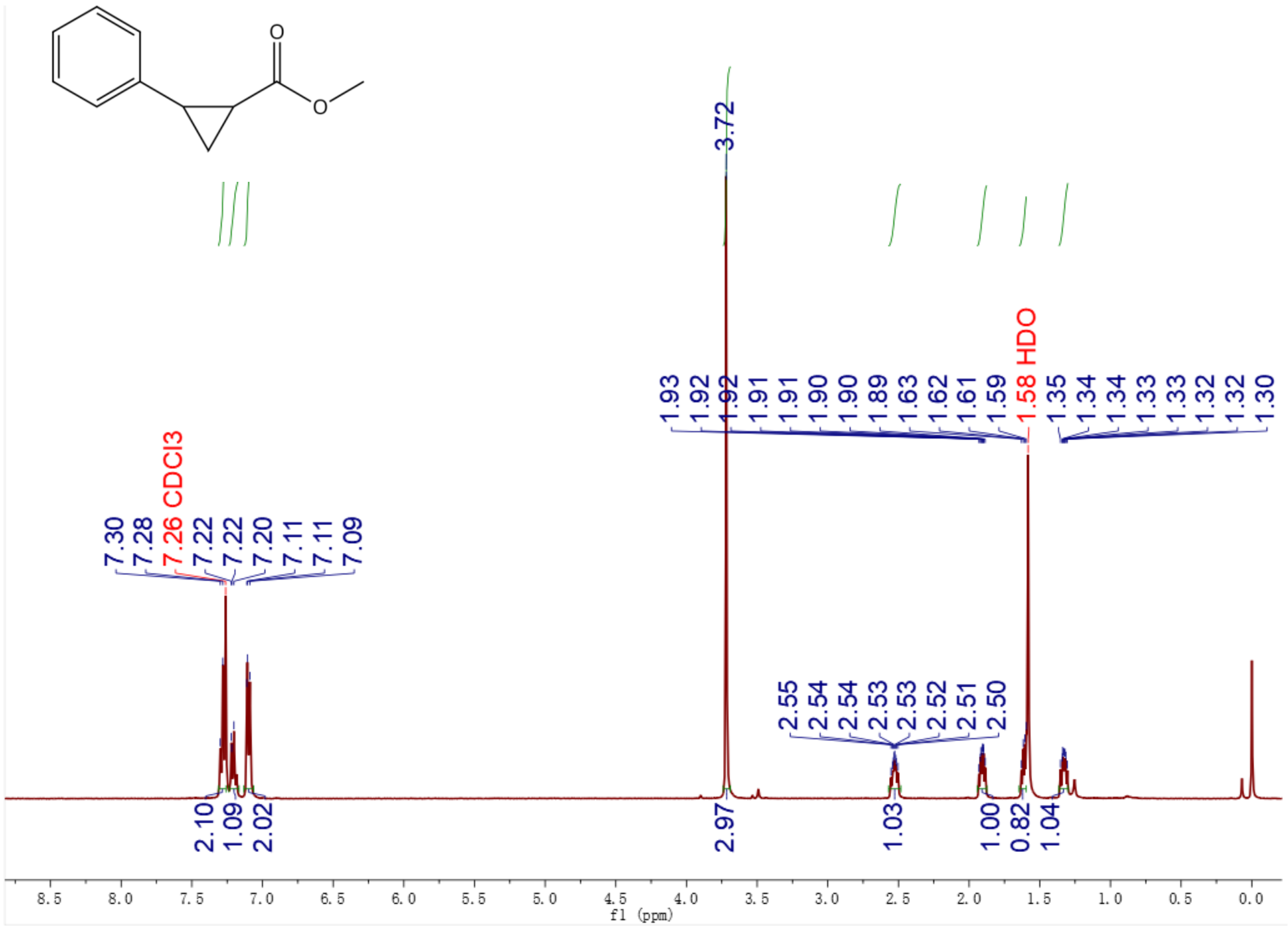

Figure S24. ^{1}H NMR spectrum of compound **5** (400 MHz, $CDCl_3$)

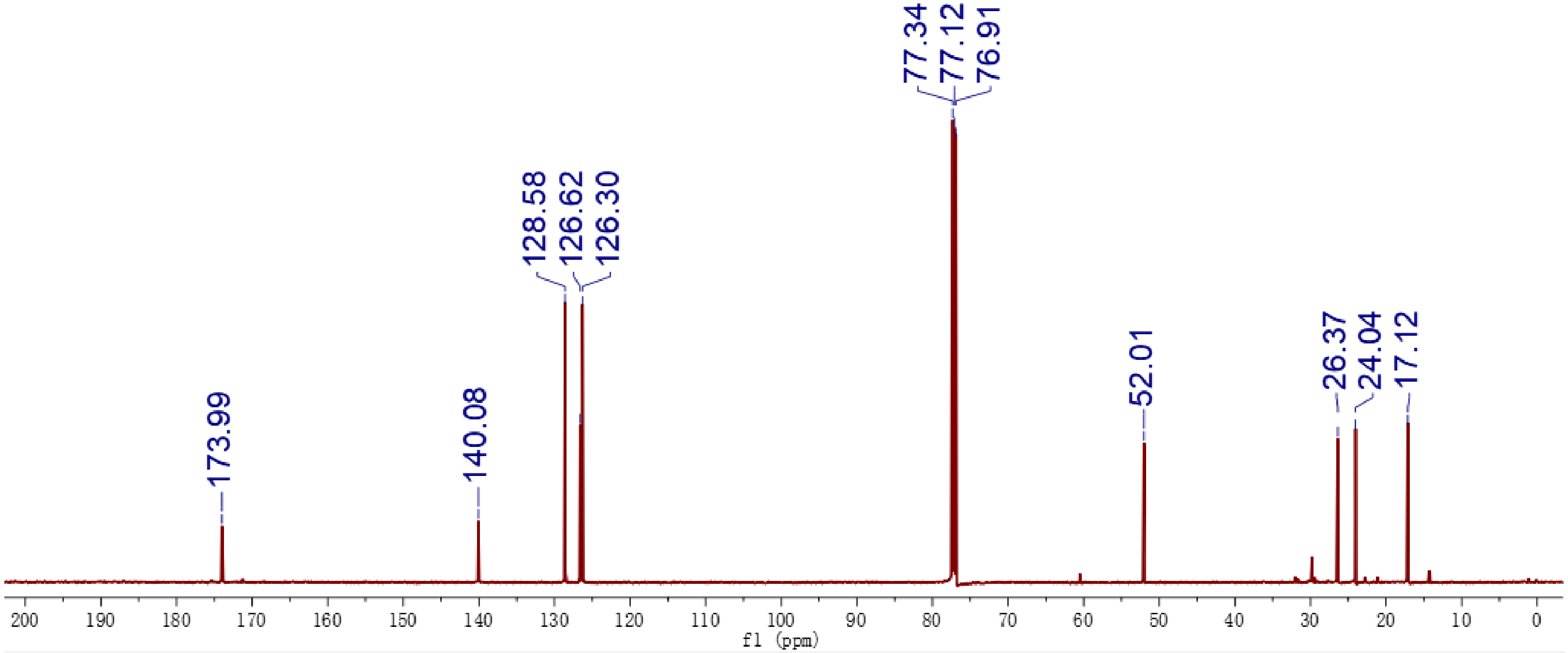

Figure S25. ^{13}C NMR spectrum of compound **5** (101 MHz, $CDCl_3$)

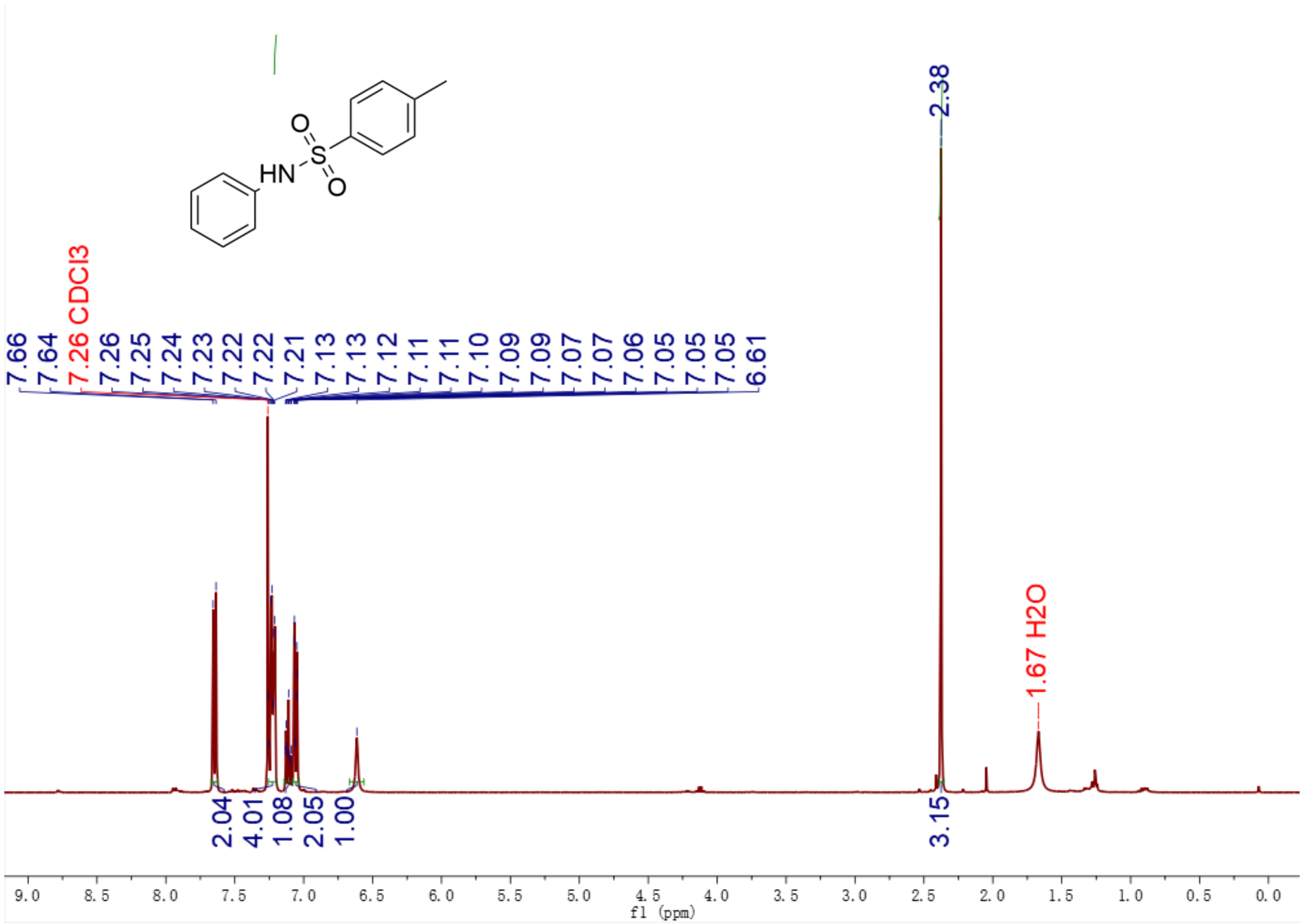

Figure S26. ^{1}H NMR spectrum of compound **9a** (400 MHz, $CDCl_3$)

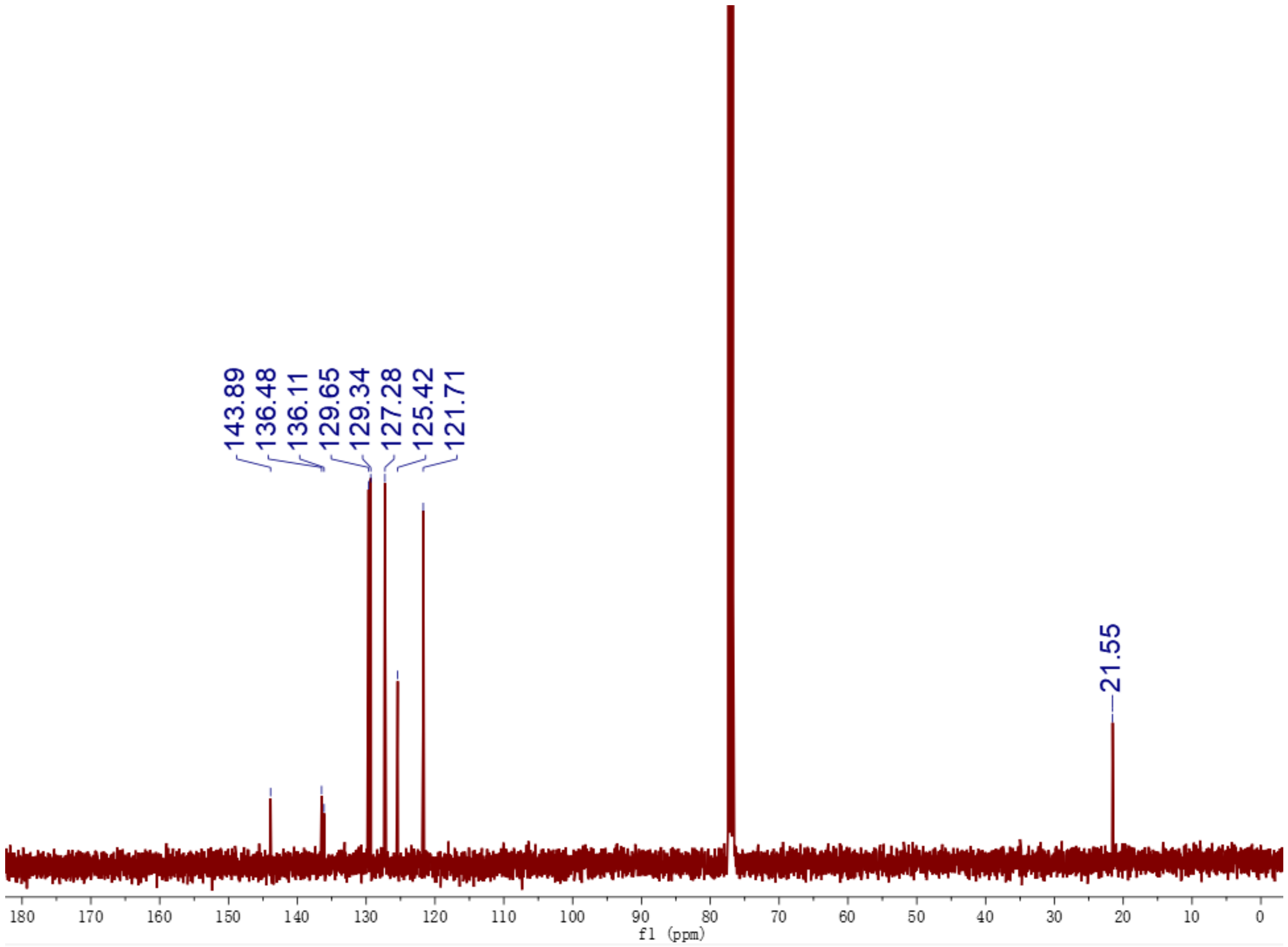

Figure S27. ^{13}C NMR spectrum of compound **9a** (101 MHz, $CDCl_3$)

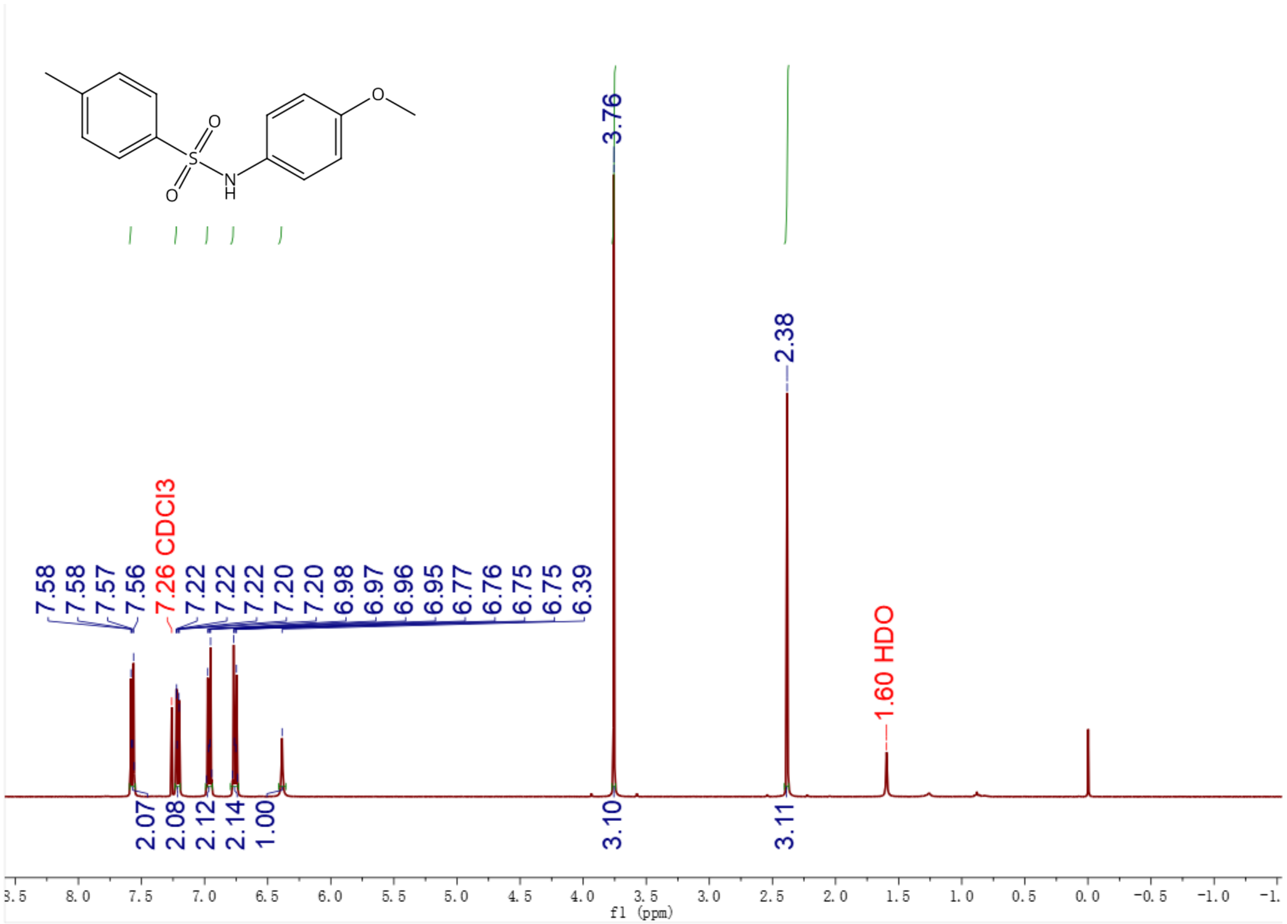

Figure S28. ^{1}H NMR spectrum of compound **9b** (400 MHz, $CDCl_3$)

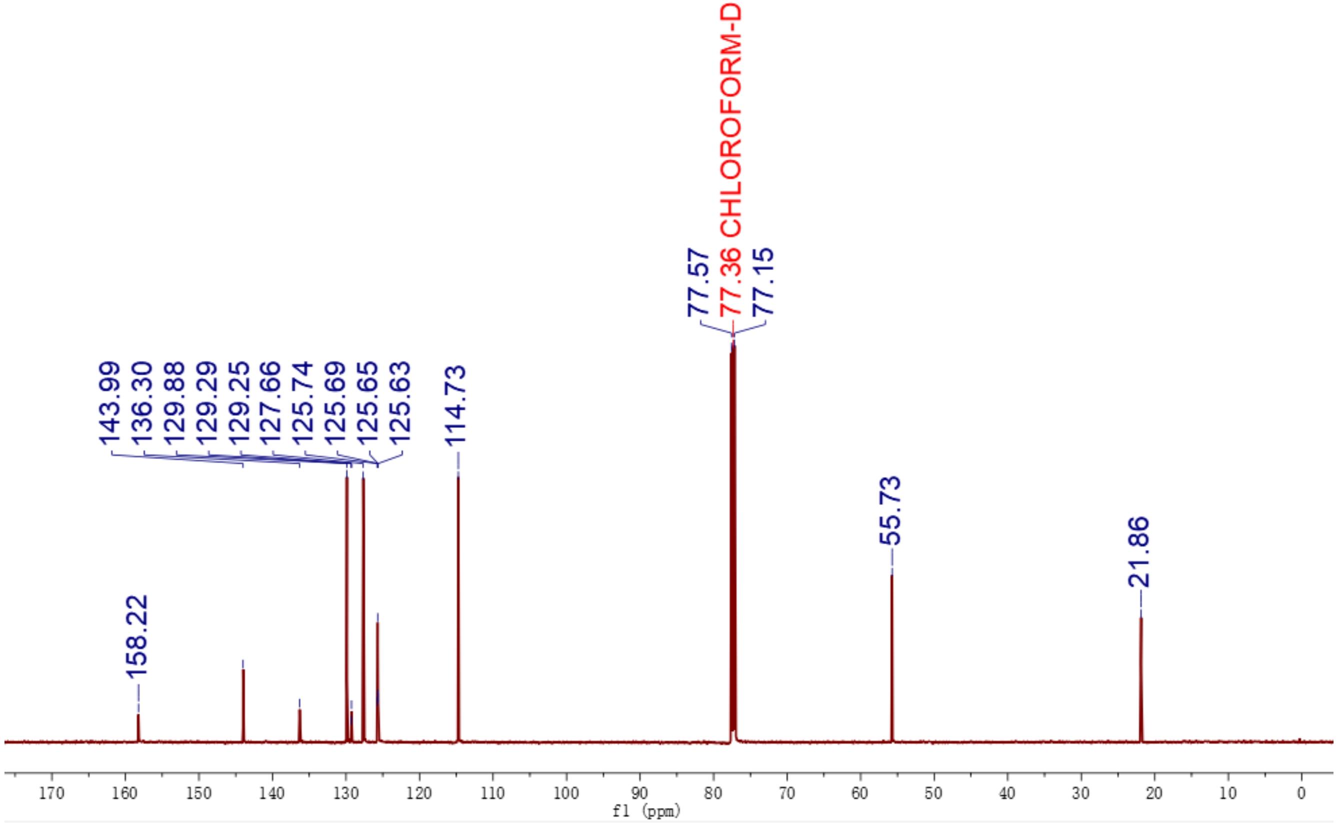

Figure S29. ^{13}C NMR spectrum of compound **9b** (101 MHz, $CDCl_3$)

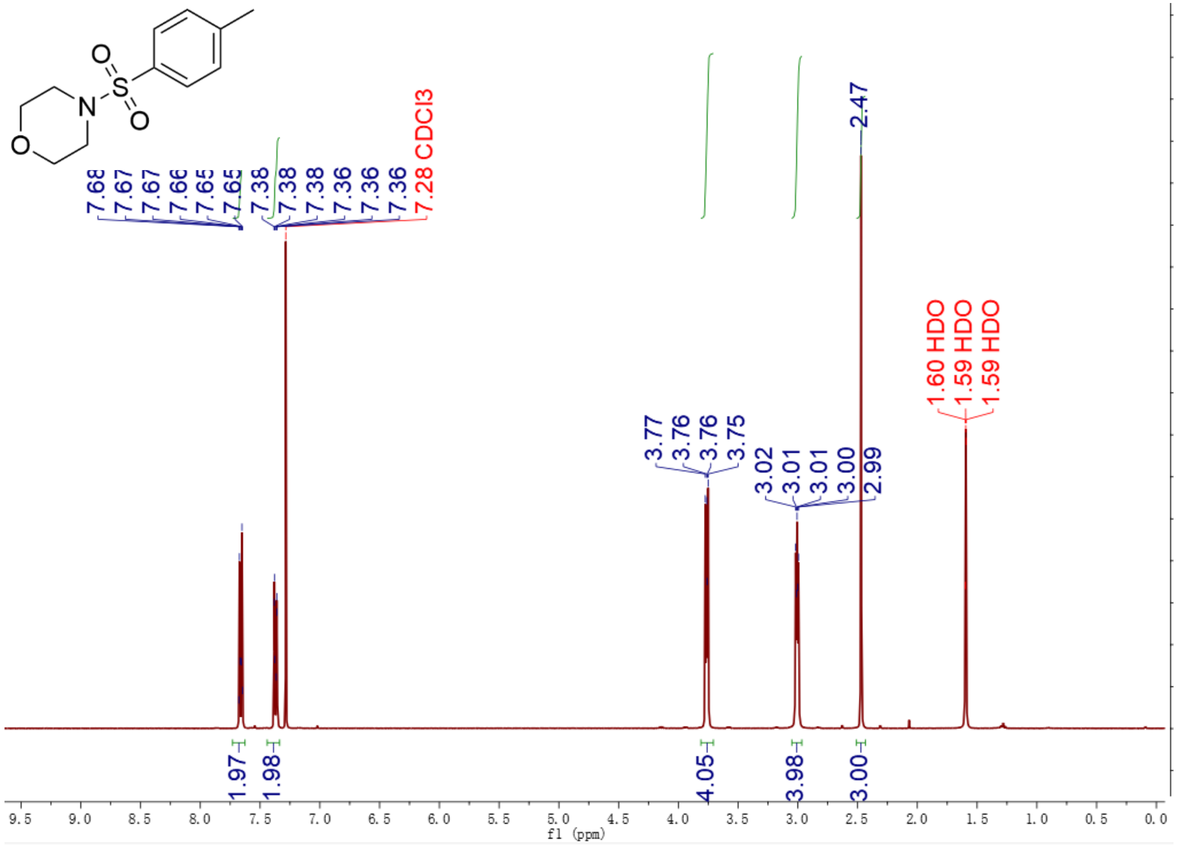

Figure S30. ^{1}H NMR spectrum of compound **9c** (400 MHz, $CDCl_3$)

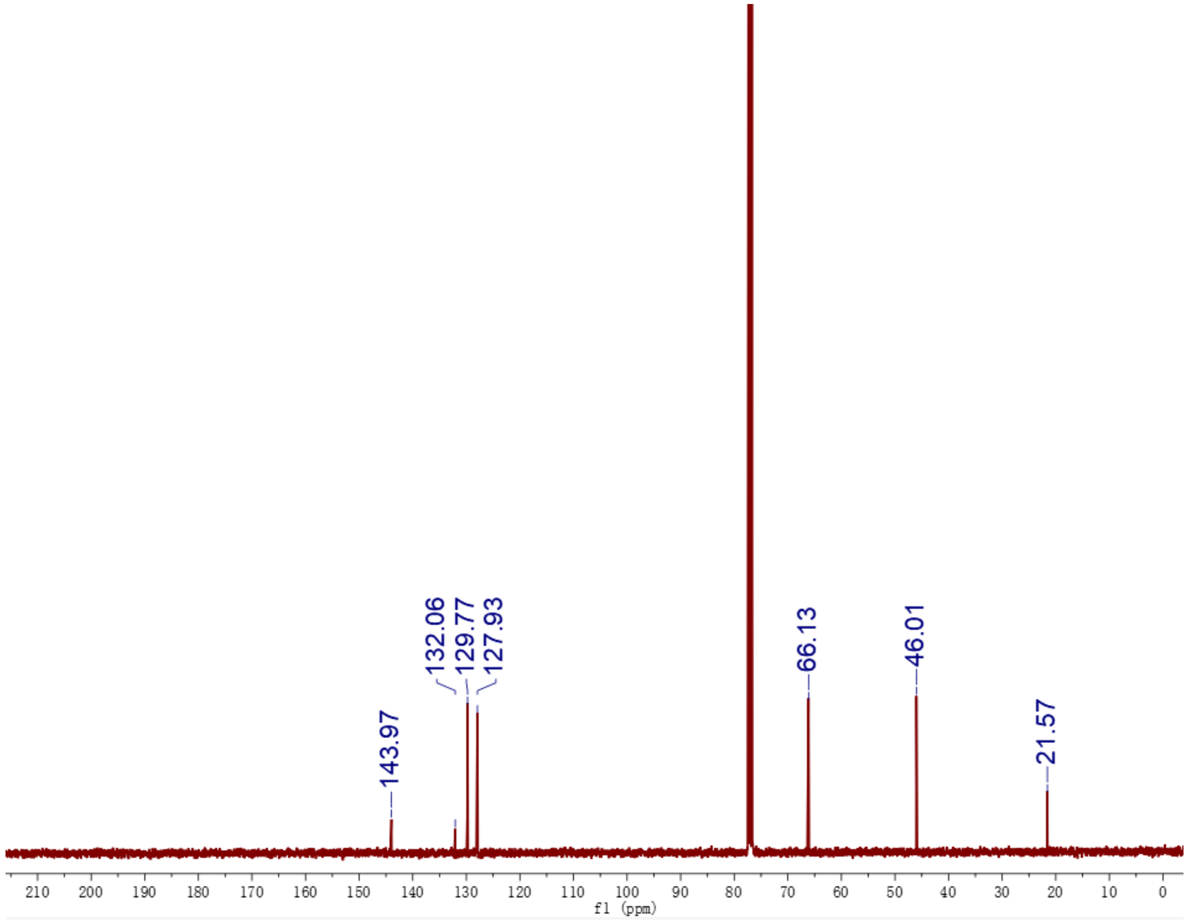

Figure S31. ^{13}C NMR spectrum of compound 9**c** (101 MHz, $CDCl_3$)

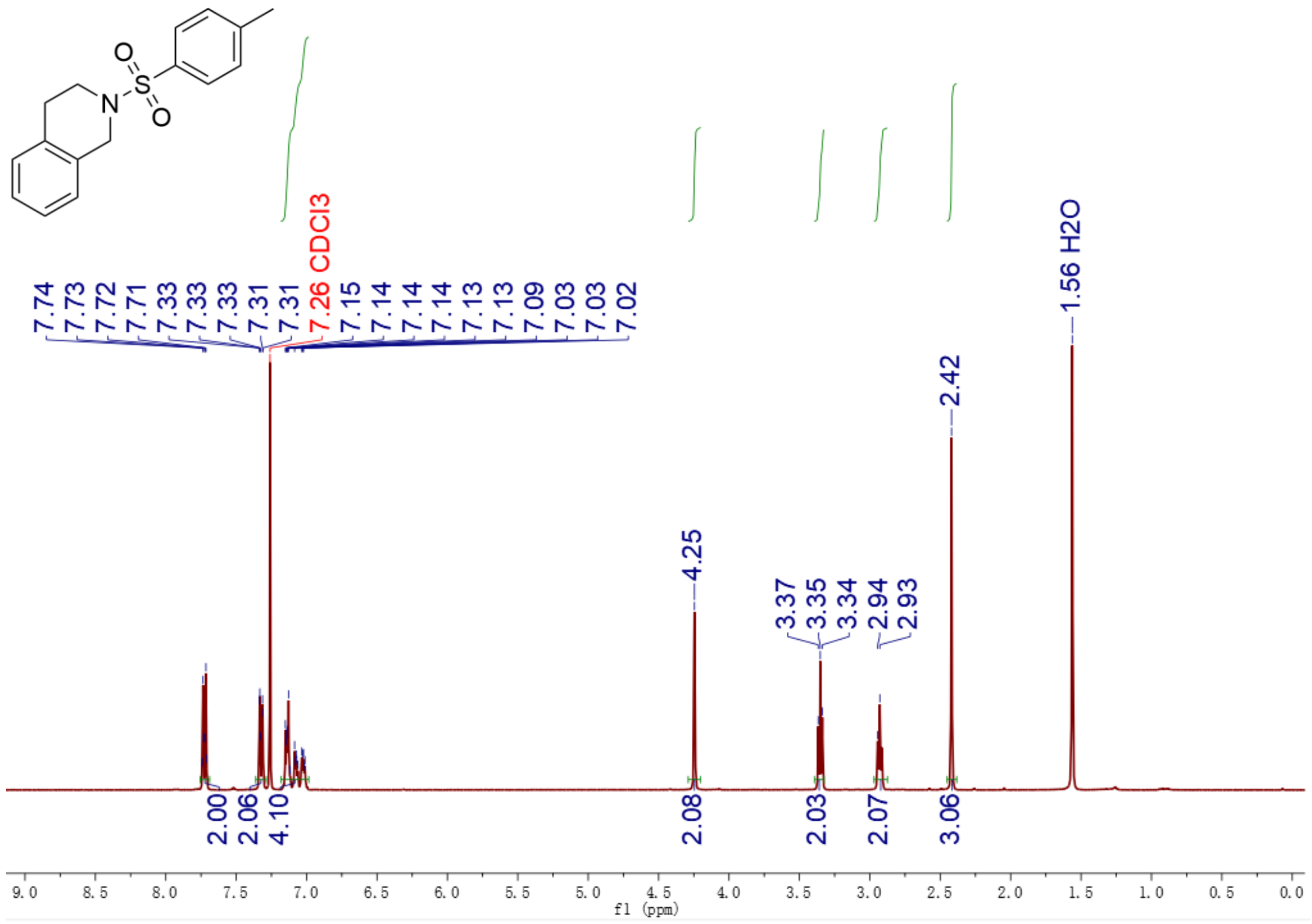

Figure S32. ^{1}H NMR spectrum of compound **9d** (400 MHz, $CDCl_3$)

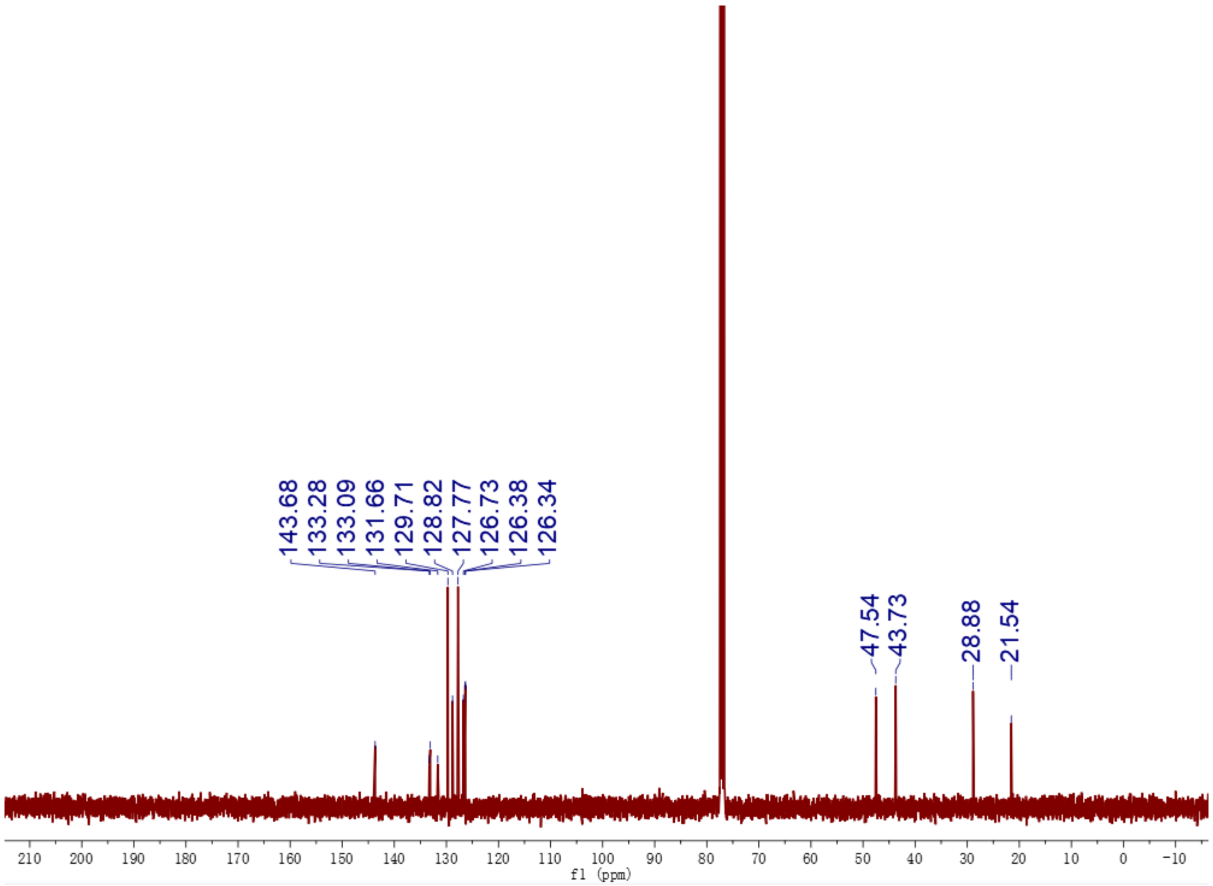

Figure S33. ^{13}C NMR spectrum of compound **9d** (101 MHz, $CDCl_3$)

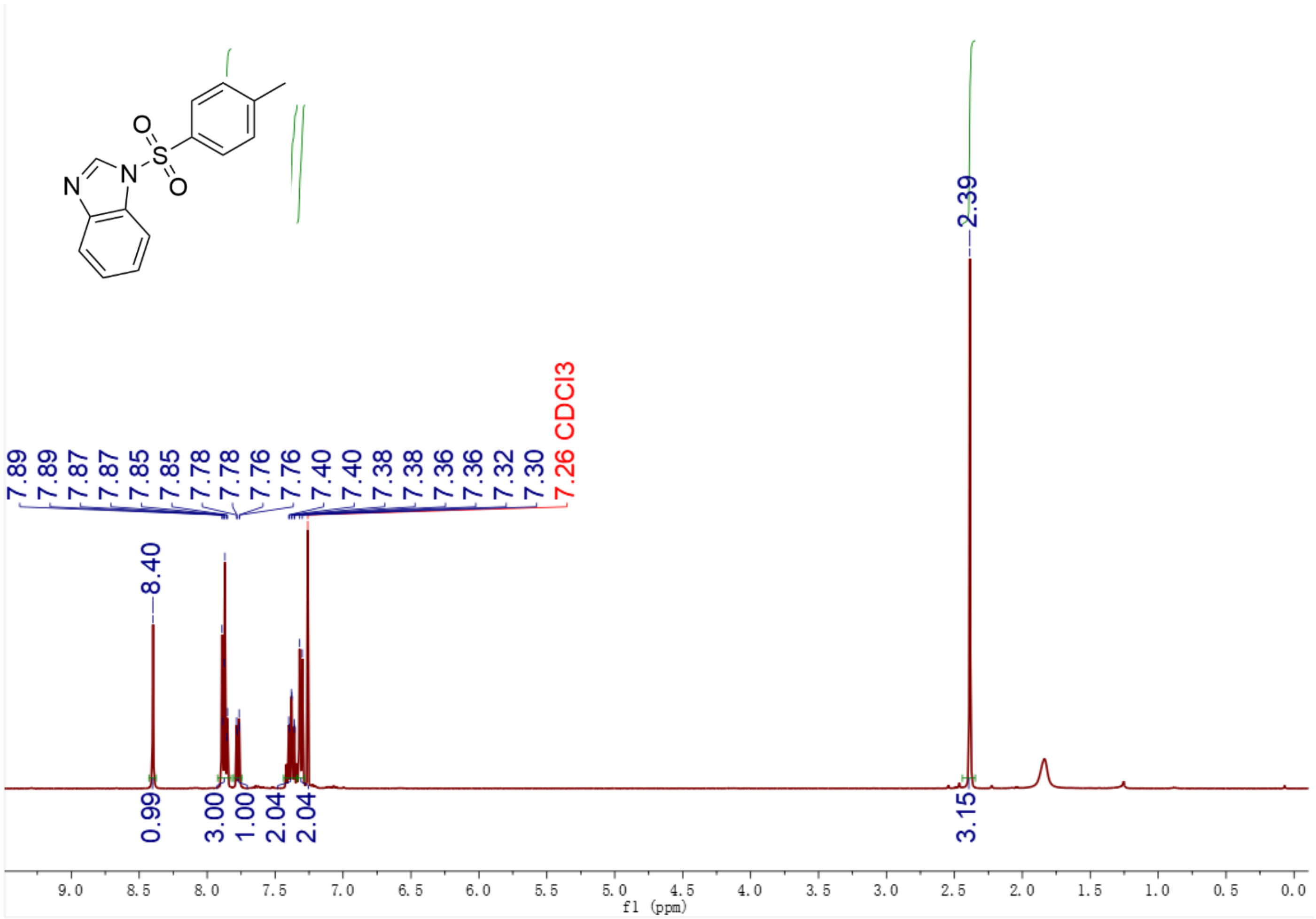

Figure S34. ^{1}H NMR spectrum of compound **9e** (400 MHz, $CDCl_3$)

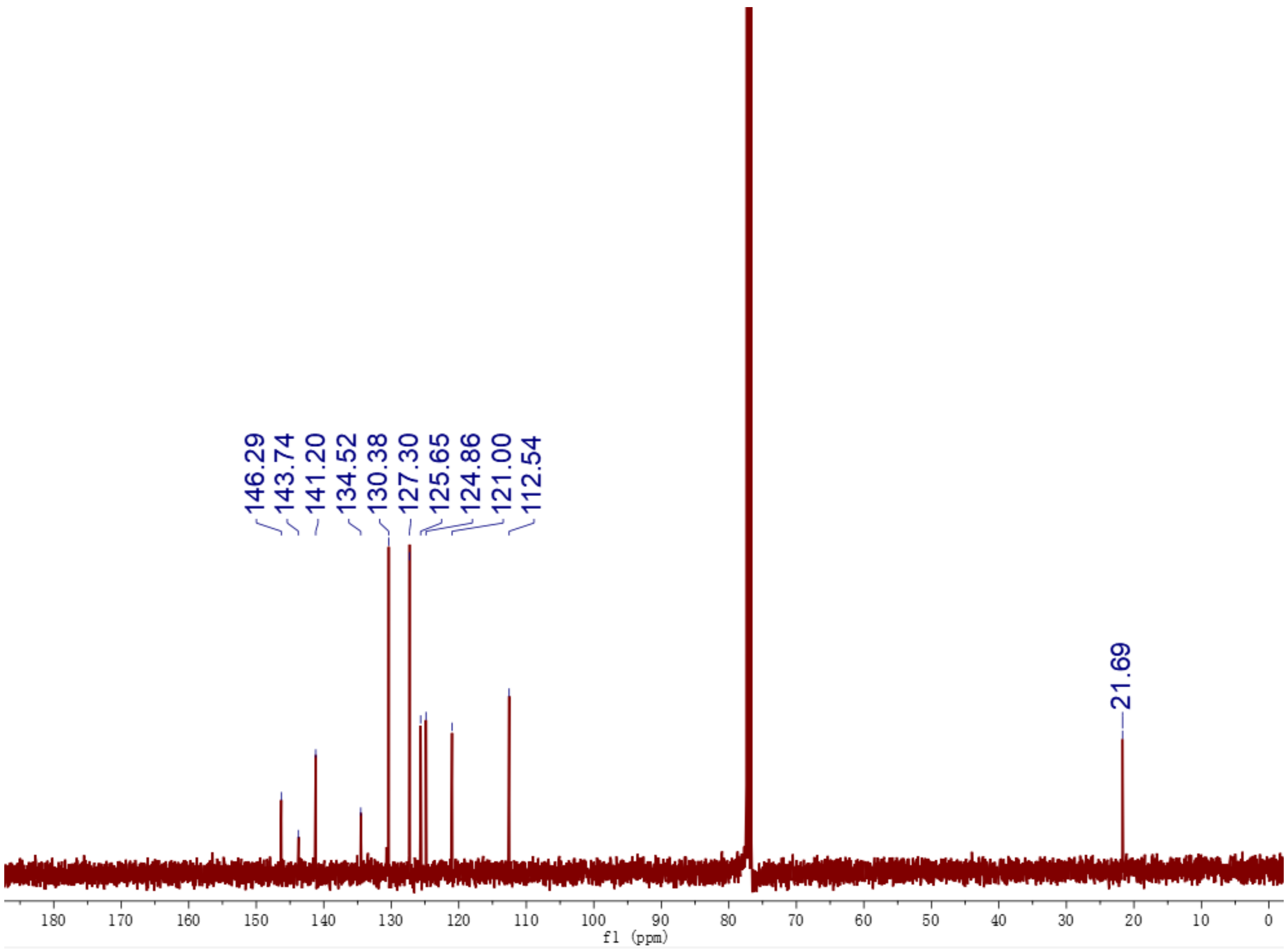

Figure S35. ^{13}C NMR spectrum of compound **9e** (101 MHz, $CDCl_3$)

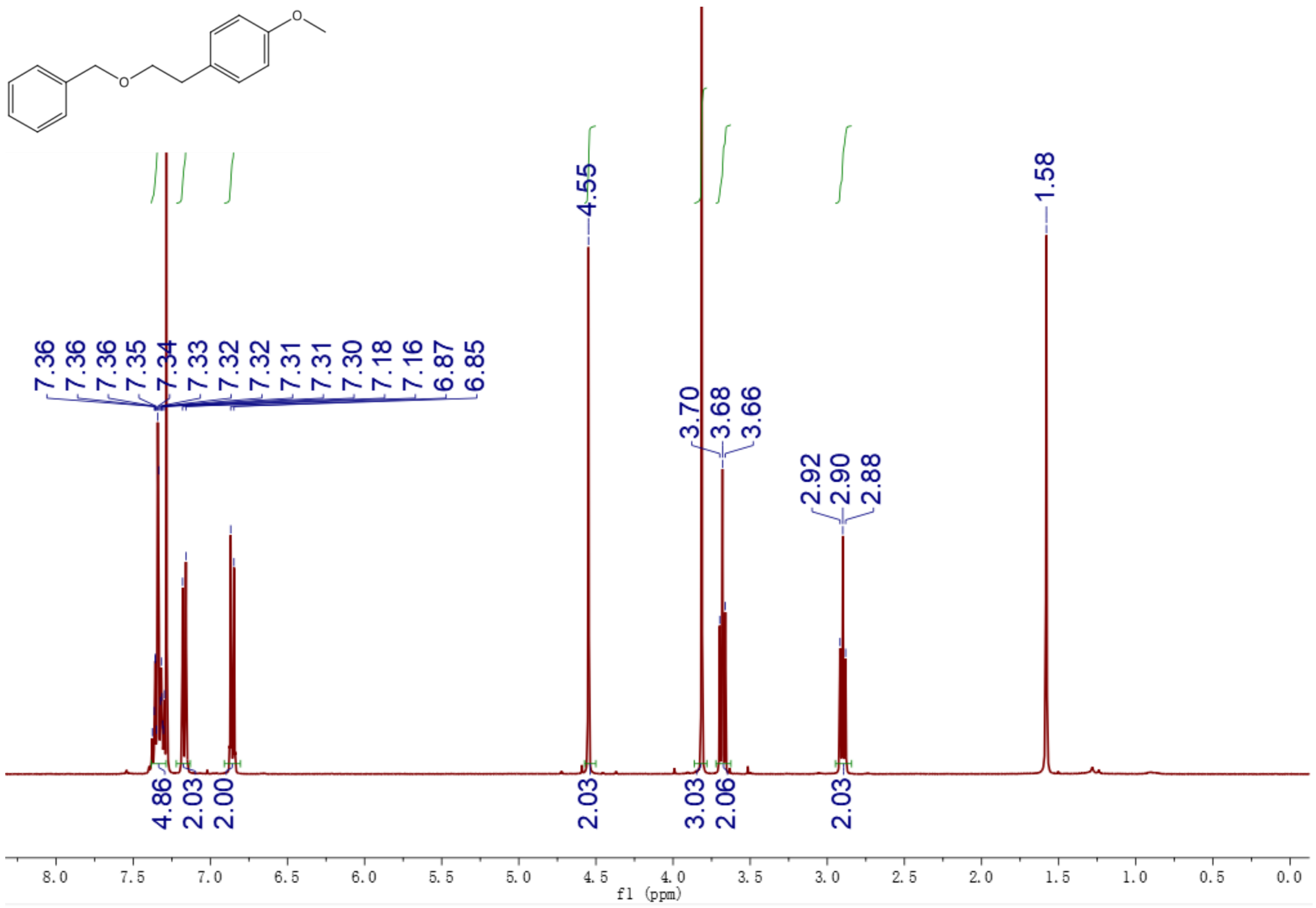

Figure S36. ^{1}H NMR spectrum of compound **11** (400 MHz, $CDCl_3$)

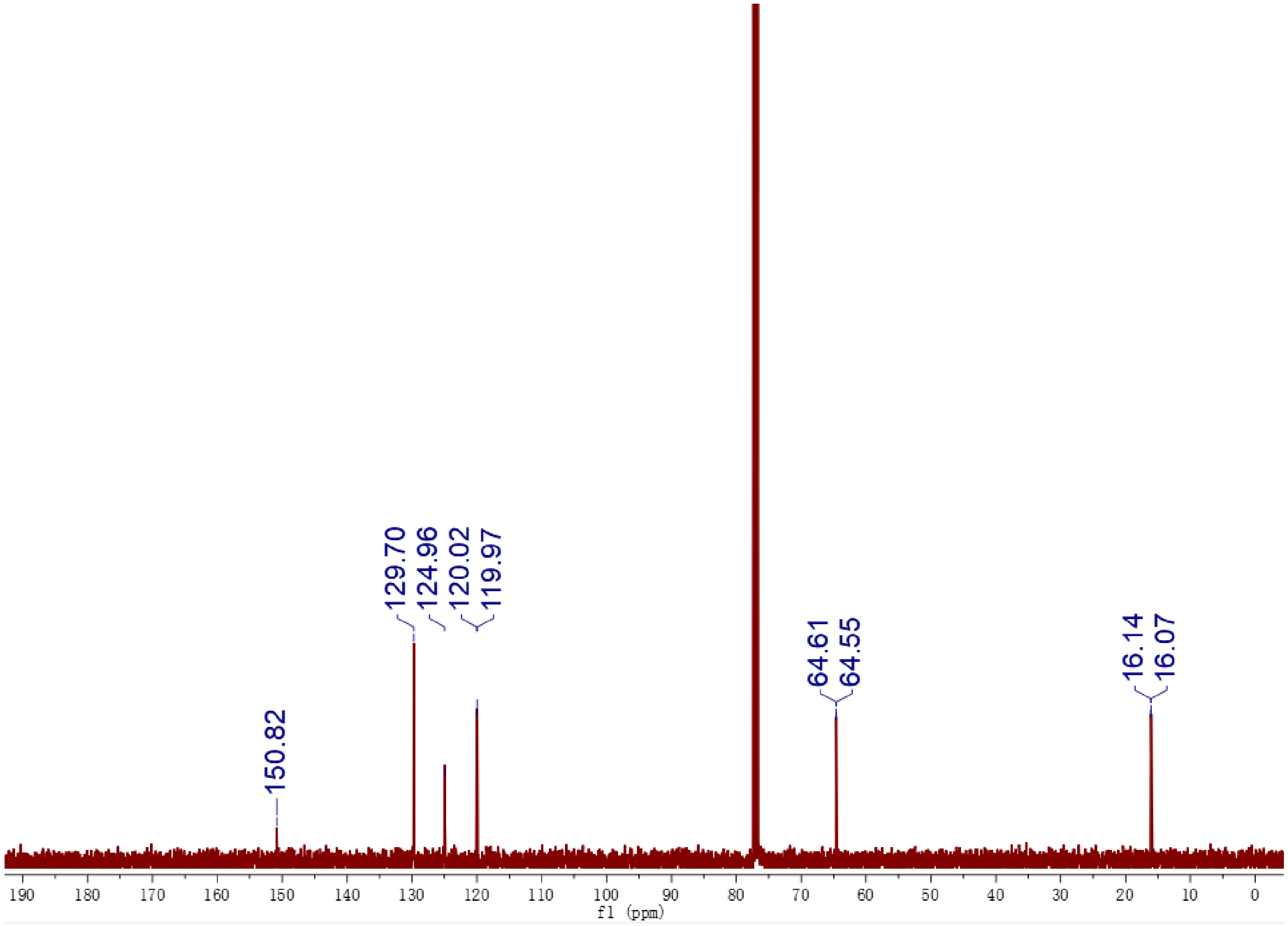

Figure S37. ^{13}C NMR spectrum of compound **11** (101 MHz, $CDCl_3$)

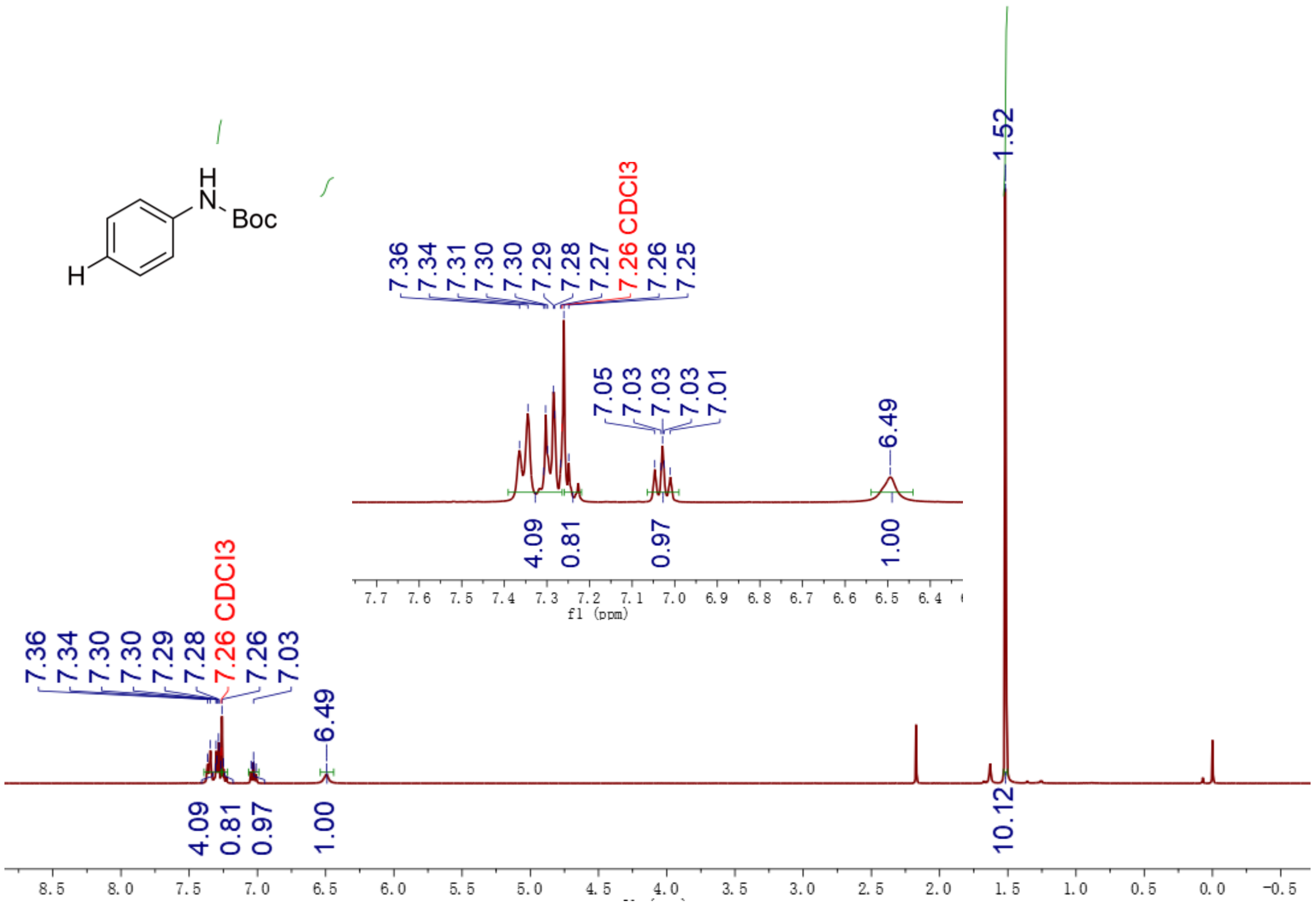

Figure S38. ^{1}H NMR spectrum of compound **2a** (400 MHz, $CDCl_3$)

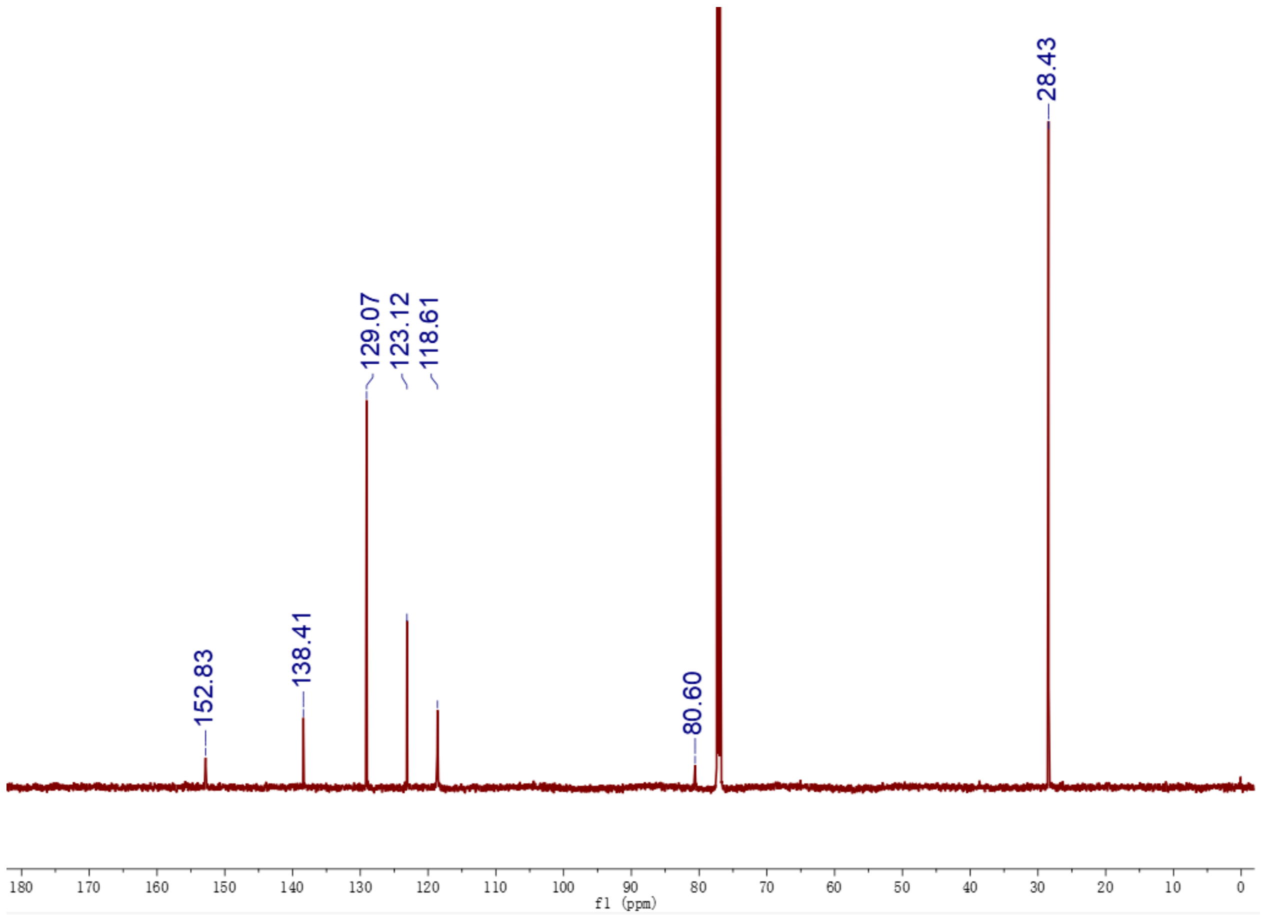

Figure S39. ^{13}C NMR spectrum of compound **2a** (101 MHz, $CDCl_3$)

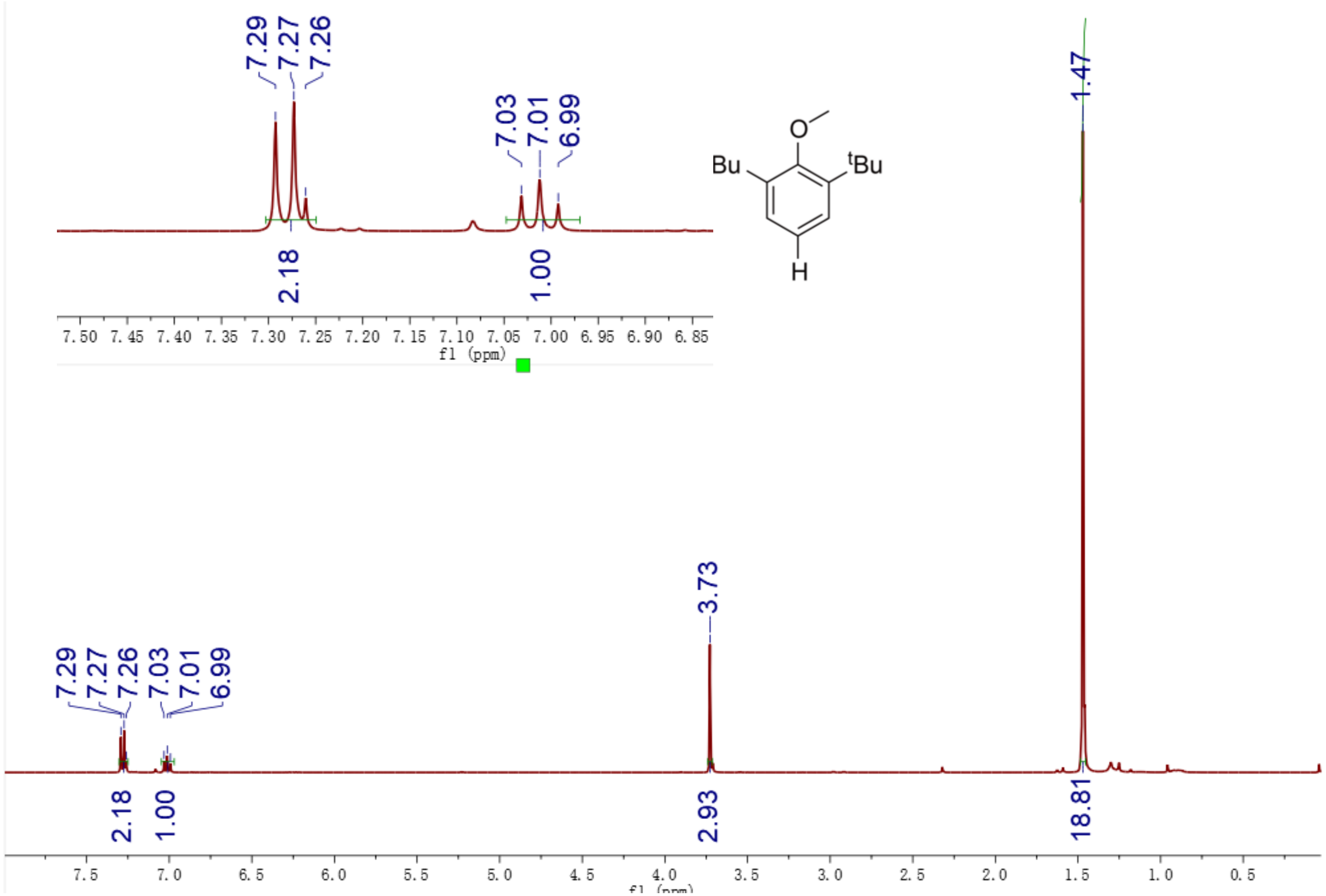

Figure S40. ^{1}H NMR spectrum of compound **2b** (400 MHz, $CDCl_3$) after 55 h under LED II

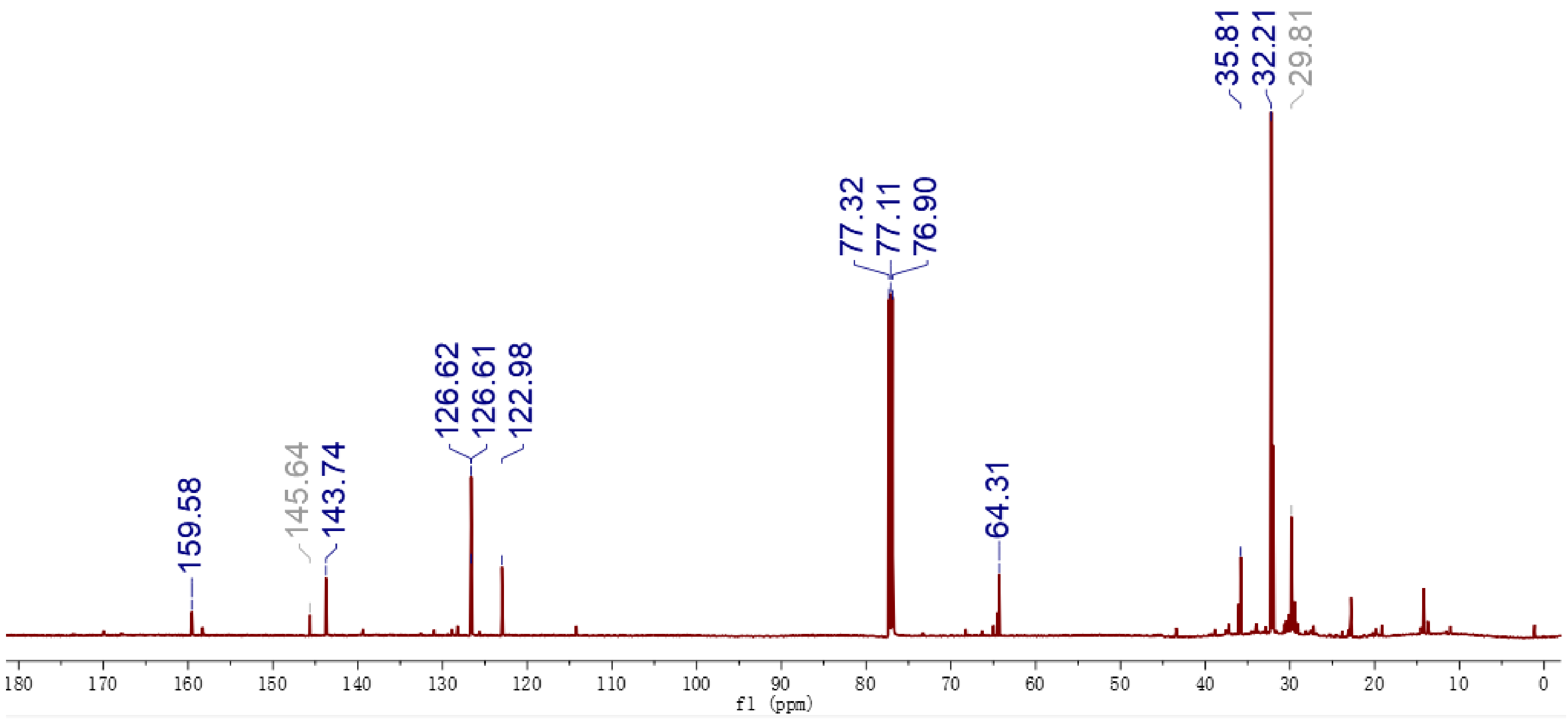

Figure S41. ^{13}C NMR spectrum of compound **2b** (101 MHz, $CDCl_3$)

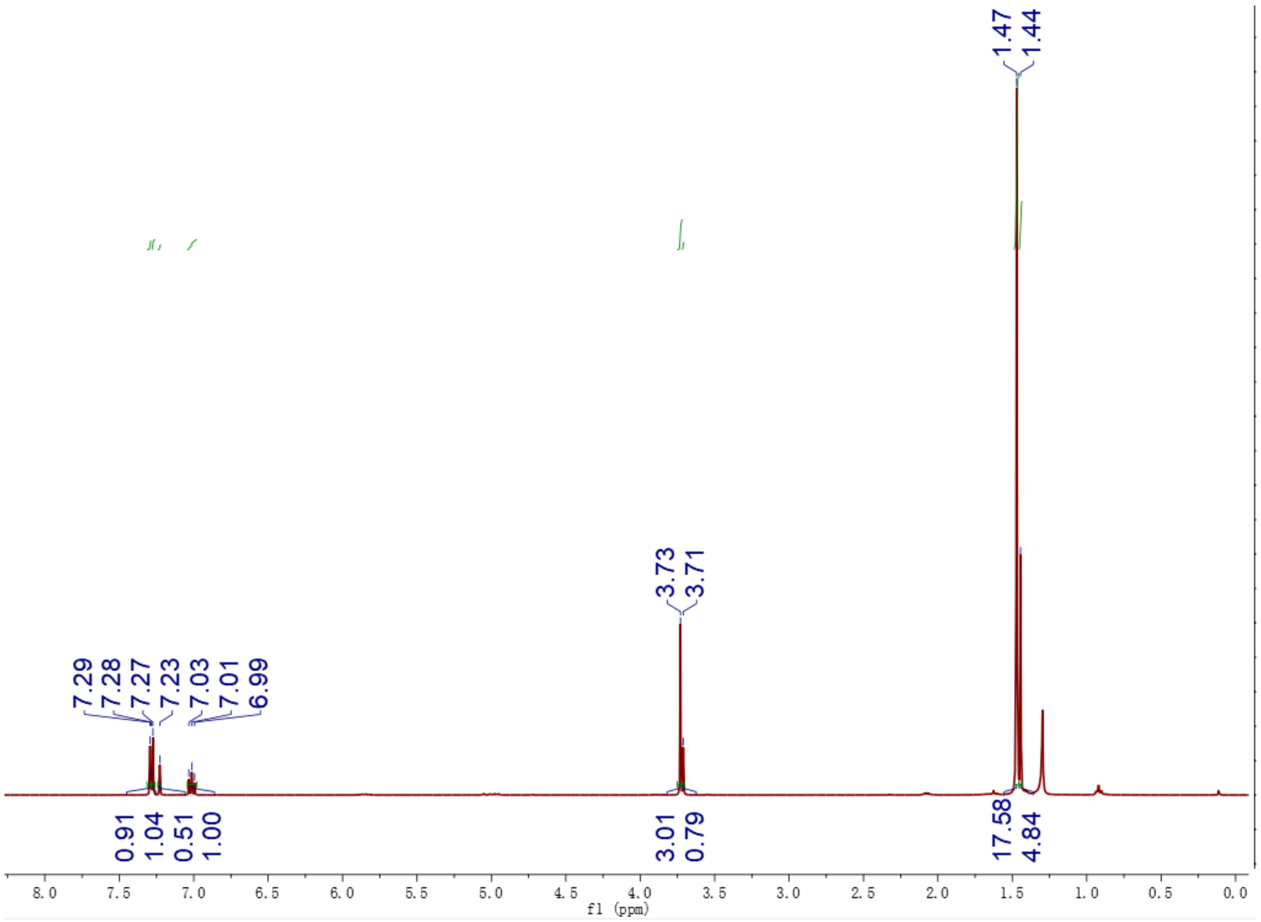

Figure S42. ^{1}H NMR spectrum of compound **2b** (400 MHz, $CDCl_3$) after 24 h under LED I

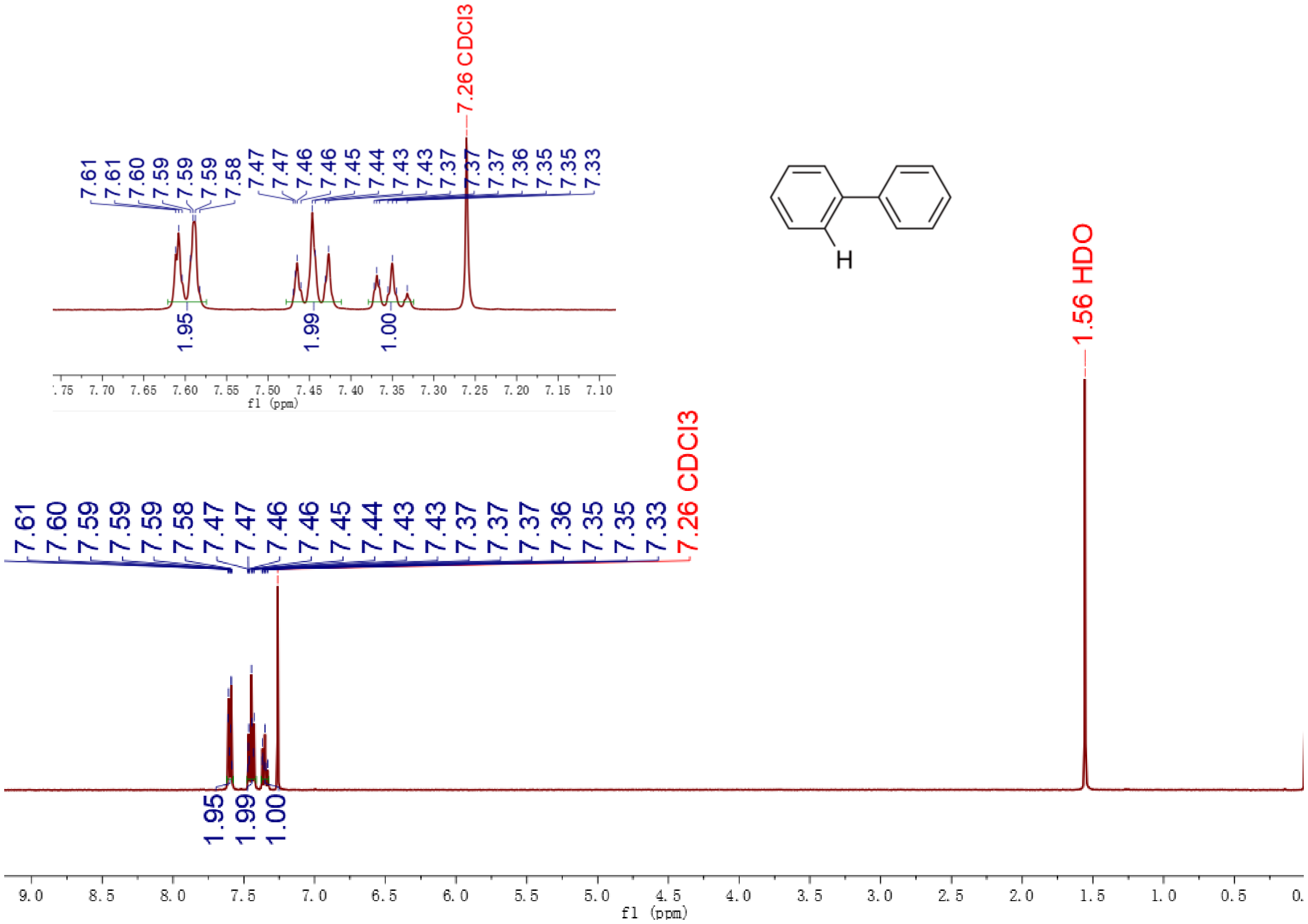

Figure S43. ^{1}H NMR spectrum of compound **2c** (400 MHz, $CDCl_3$)

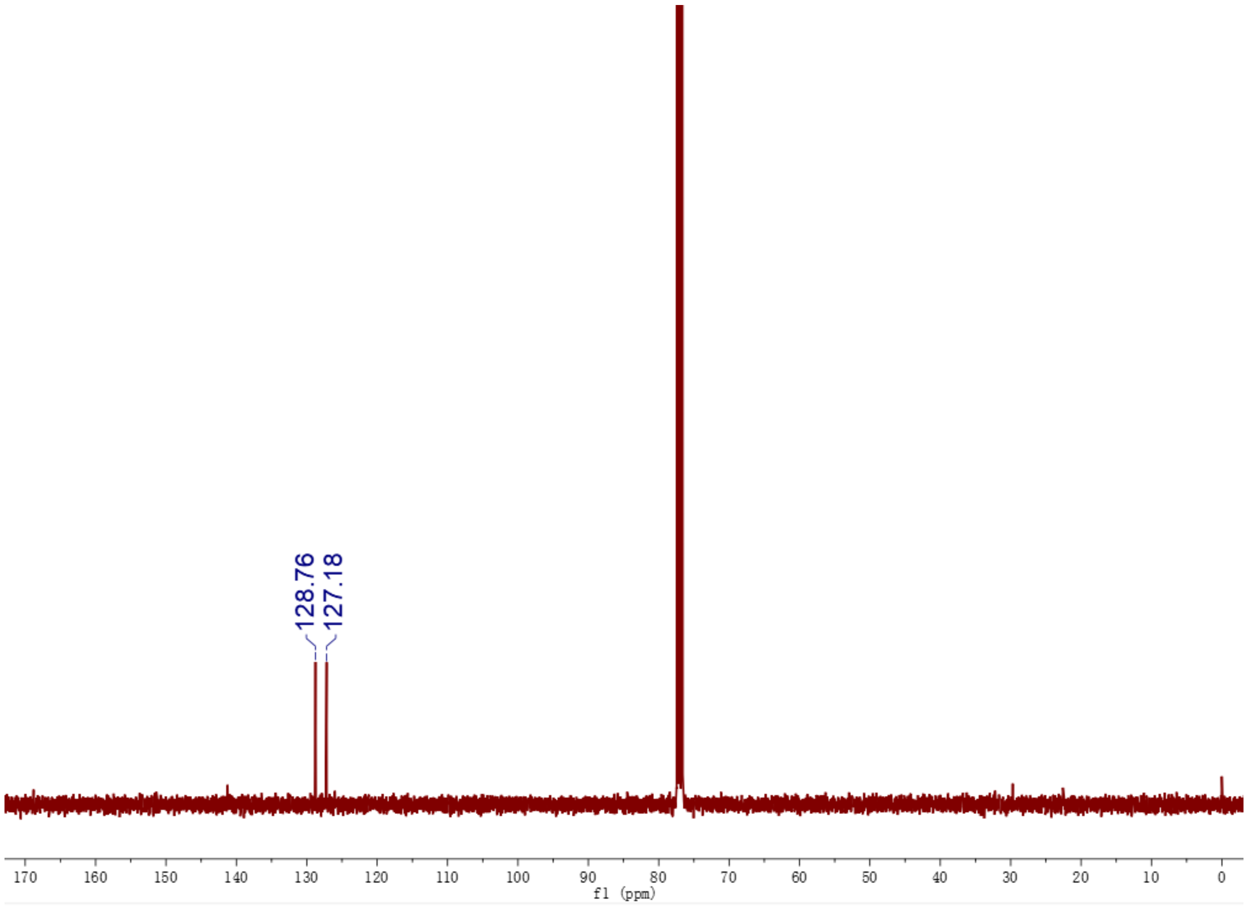

Figure S44. ^{13}C NMR spectrum of compound **2c** (101 MHz, $CDCl_3$)

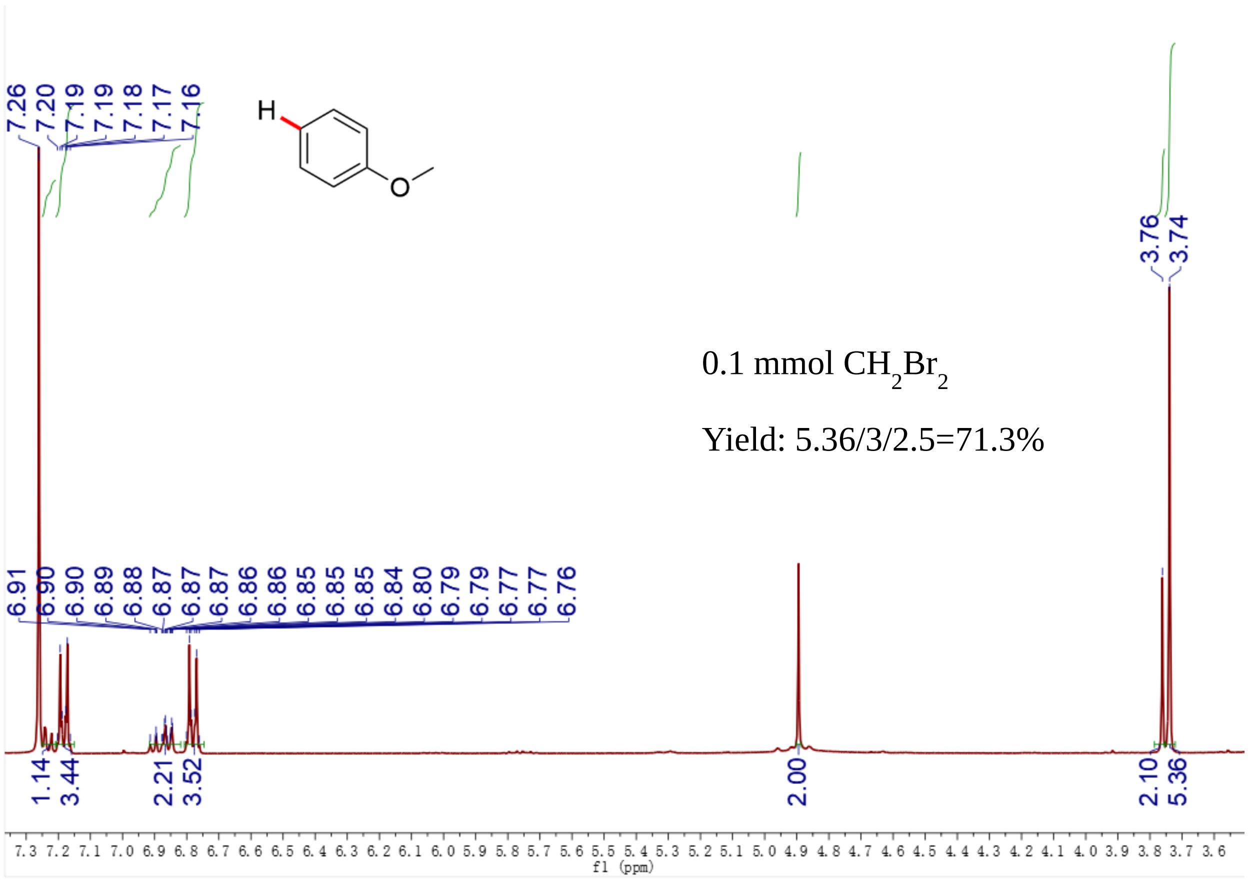

Figure S45. ^{1}H NMR spectrum of reaction mixture for **2d** (400 MHz, $CDCl_3$)

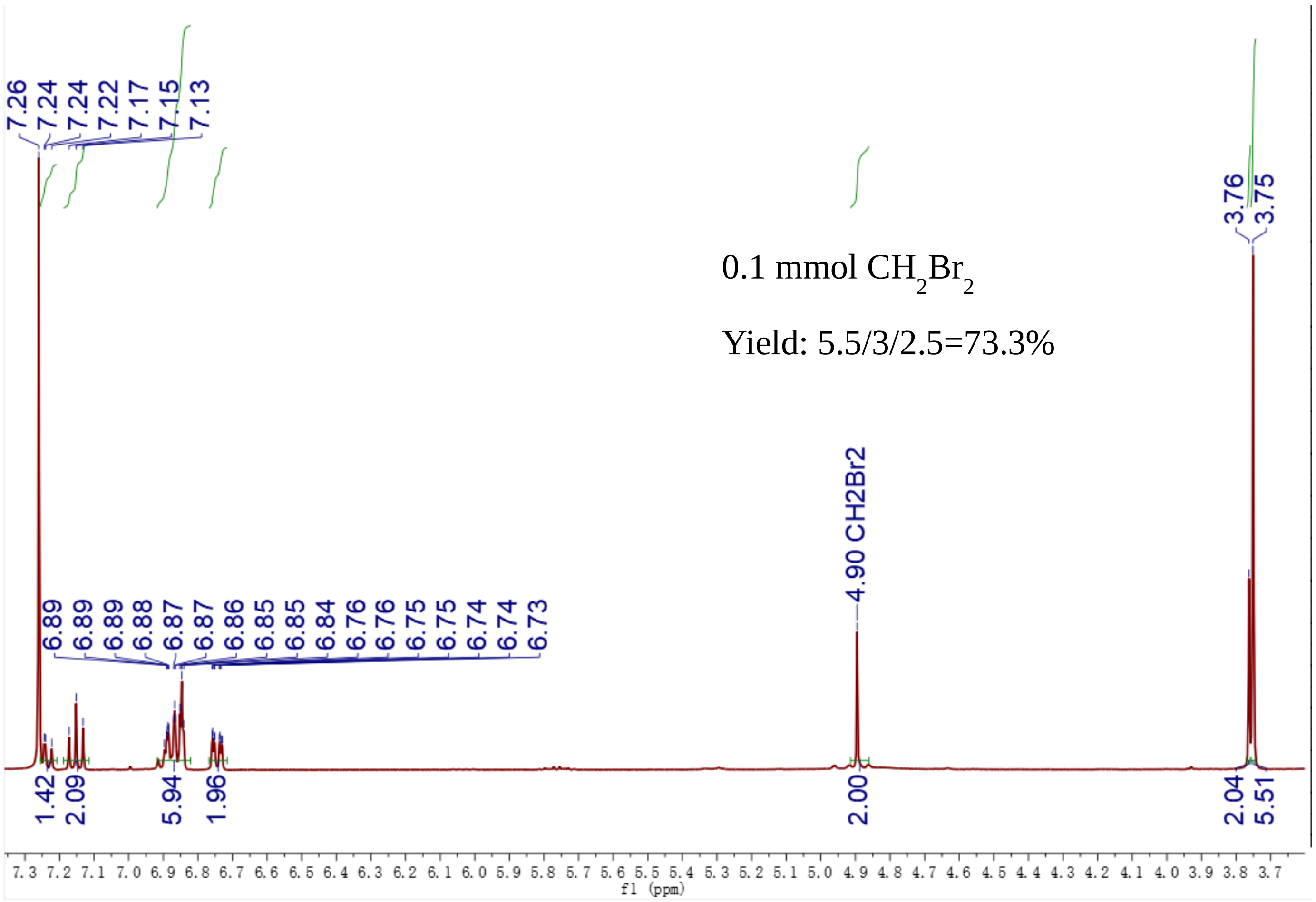

Figure S46. ^{1}H NMR spectrum of reaction mixture for **2e** (400 MHz, $CDCl_3$)

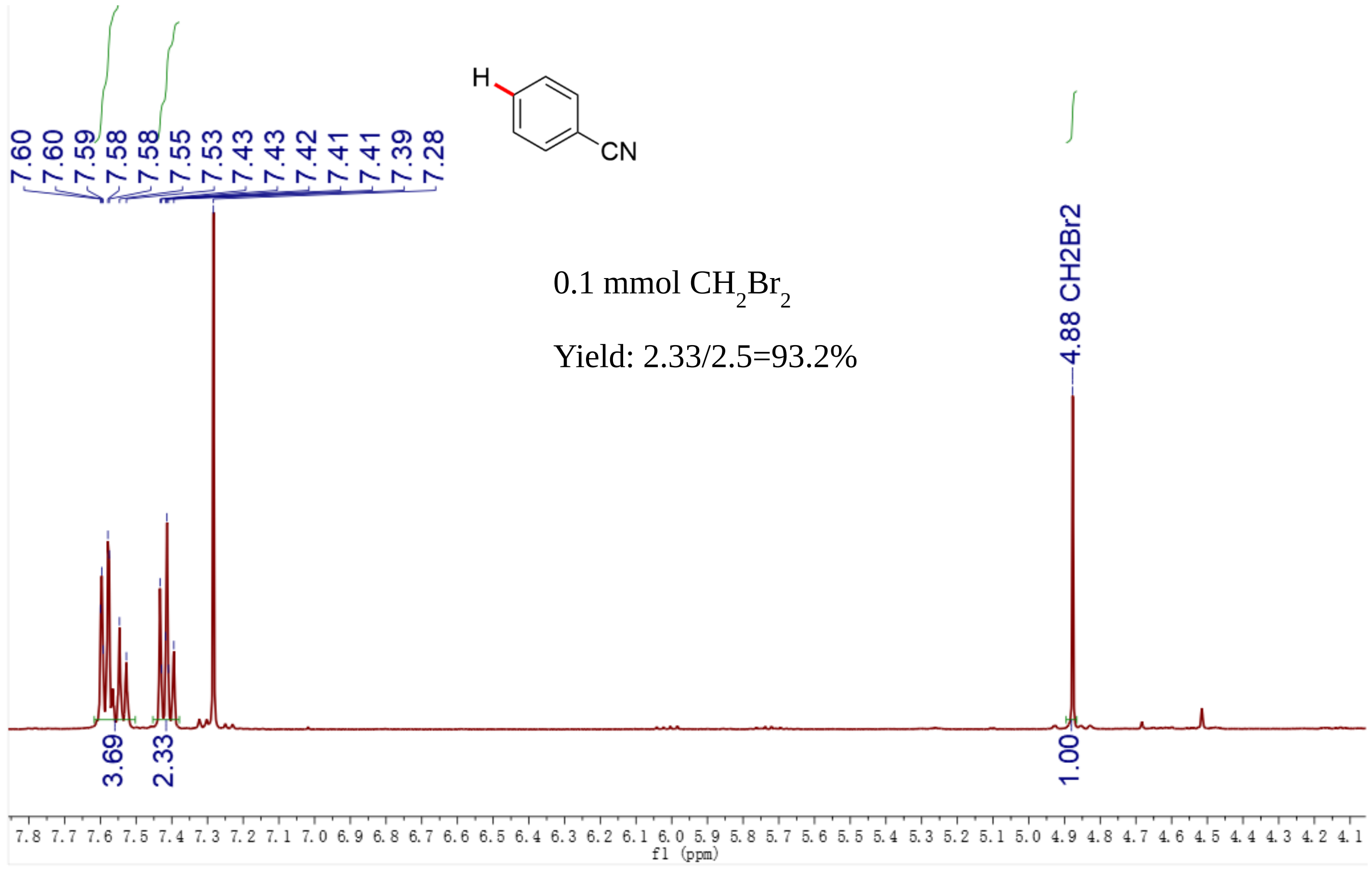

Figure S47. ^{1}H NMR spectrum of reaction mixture for **2f** (400 MHz, $CDCl_3$)

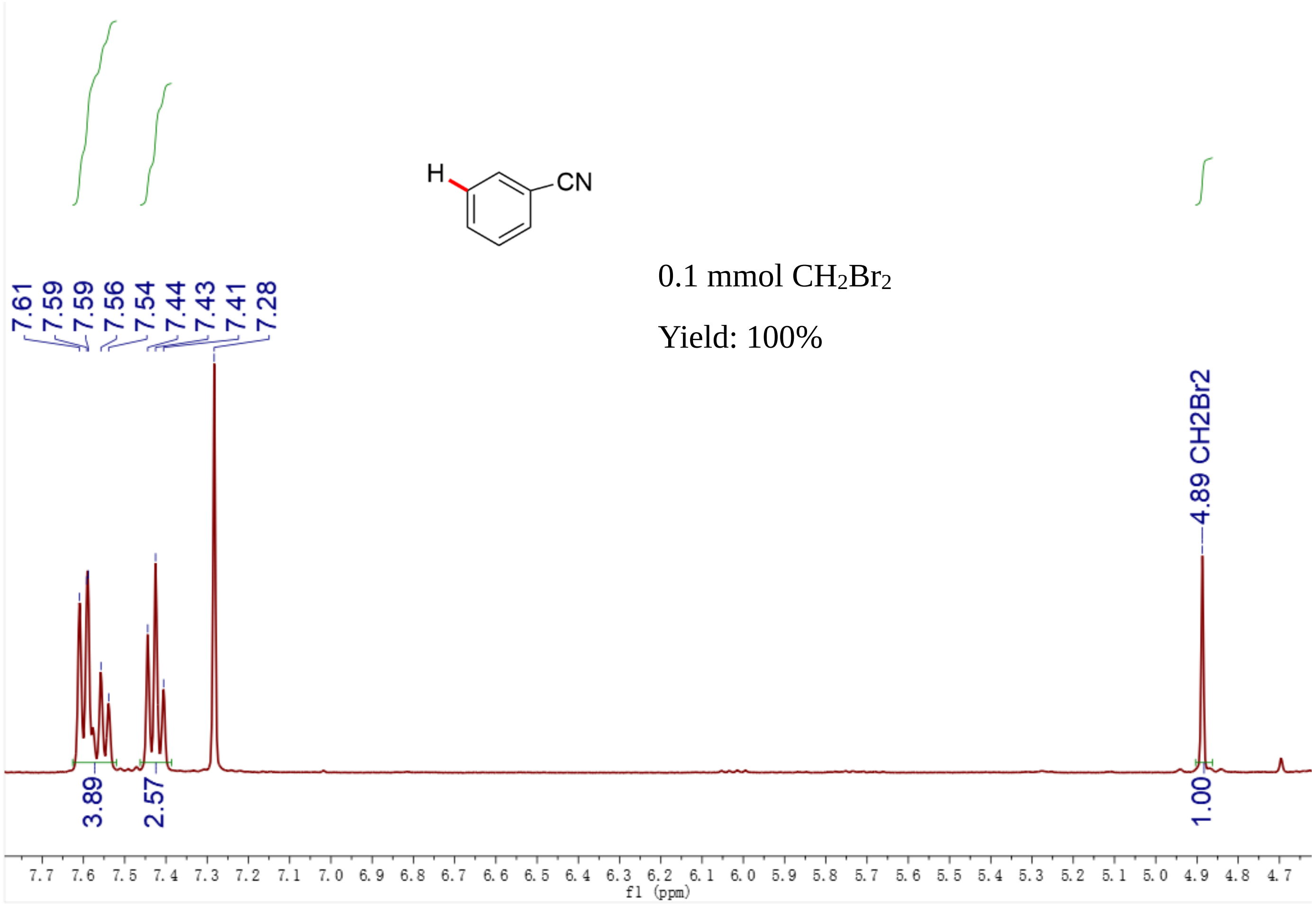

Figure S48. ^{1}H NMR spectrum of reaction mixture for **2g** (400 MHz, $CDCl_3$)

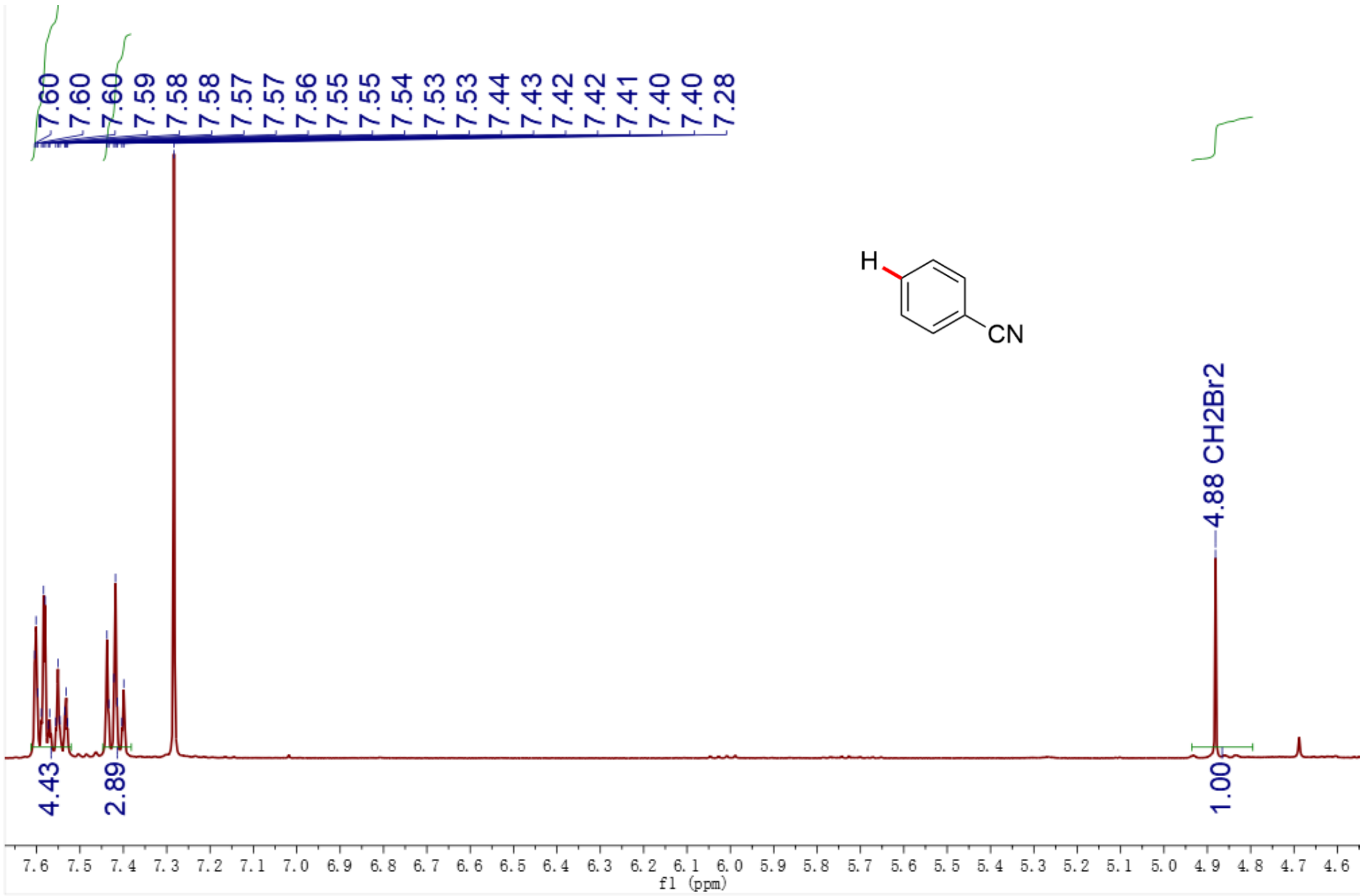

Figure S49. ^{1}H NMR spectrum of reaction mixture for **2l** (400 MHz, $CDCl_3$)

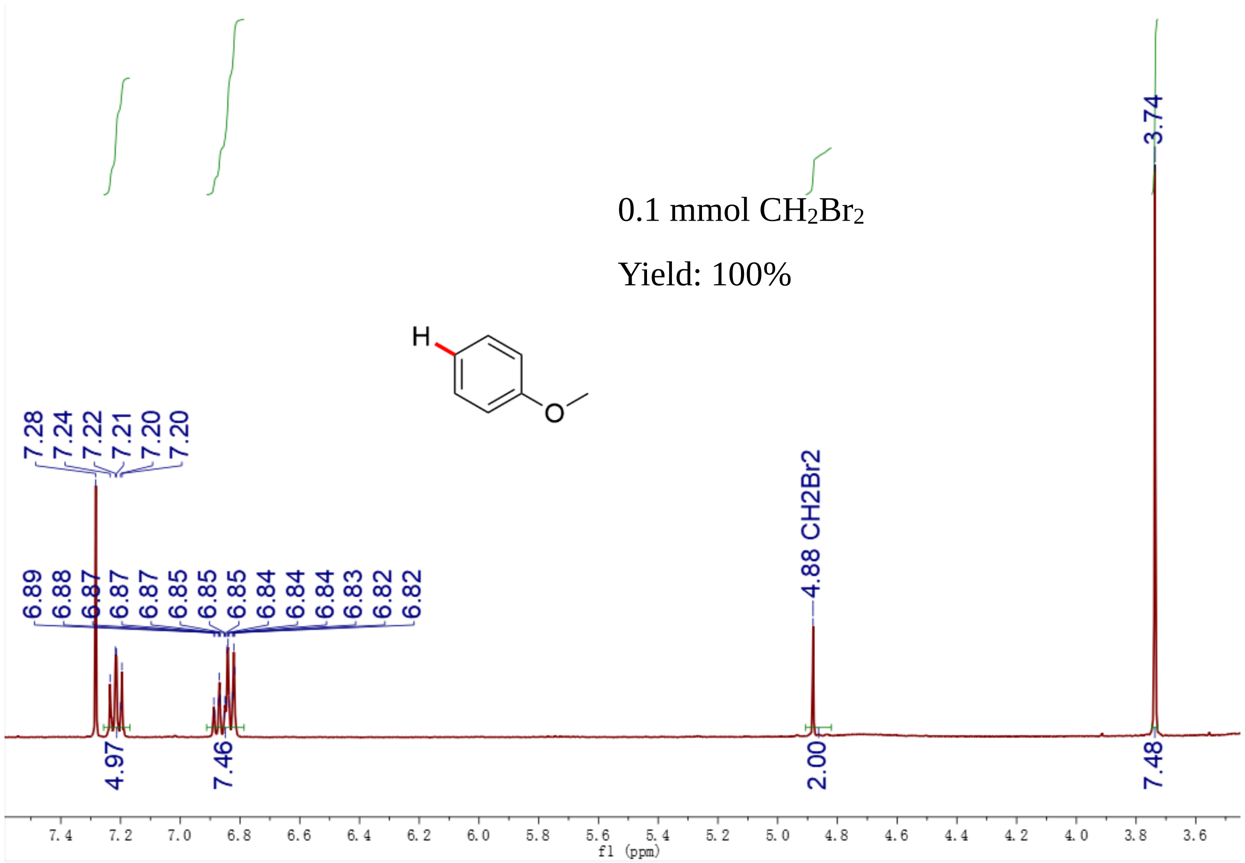

Figure S50. ^{1}H NMR spectrum of reaction mixture for **2o** (400 MHz, $CDCl_3$)

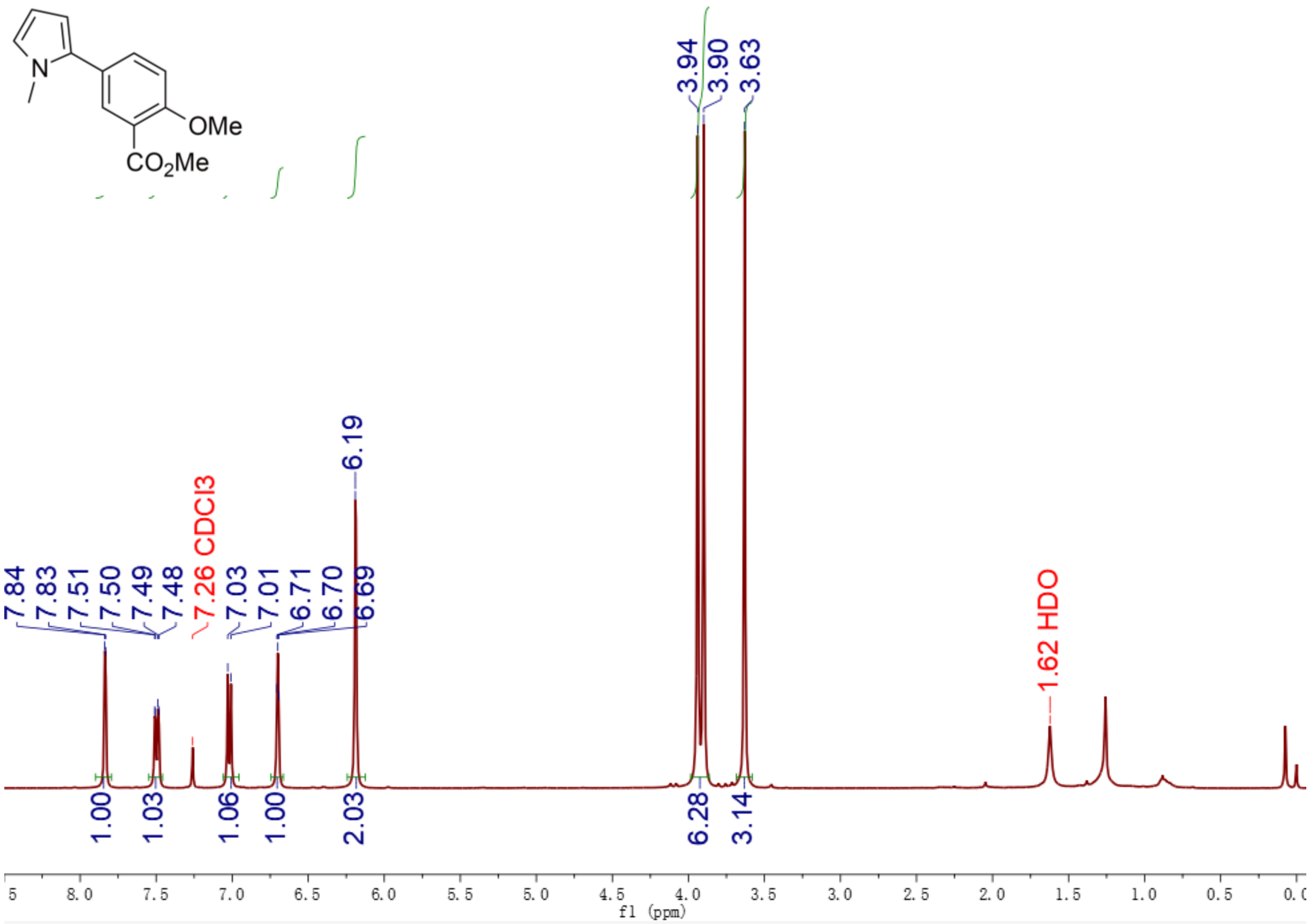

Figure S51. ^{1}H NMR spectrum of compound **2p** (400 MHz, $CDCl_3$)

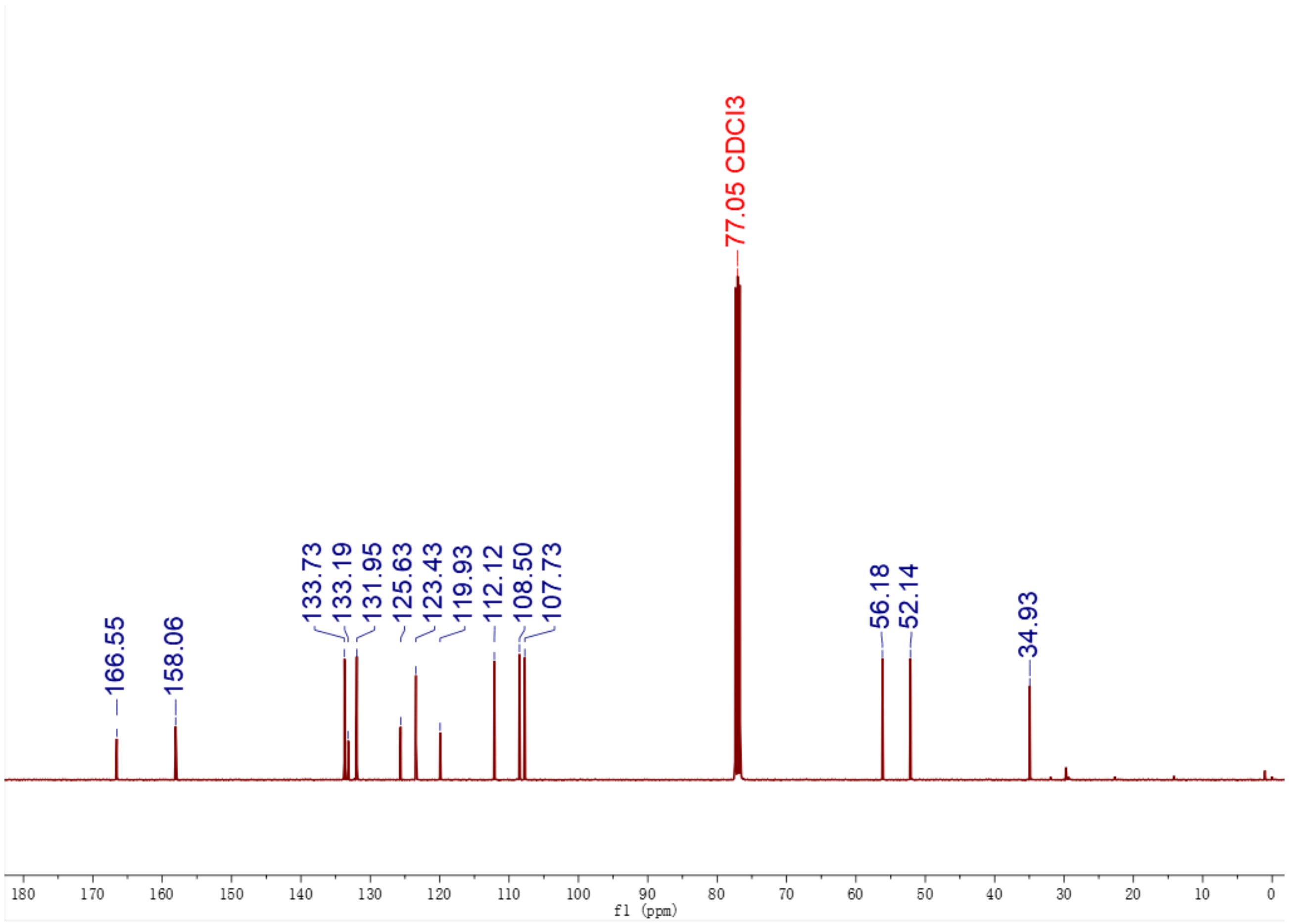

Figure S52. ^{13}C NMR spectrum of compound **2p** (101 MHz, $CDCl_3$)

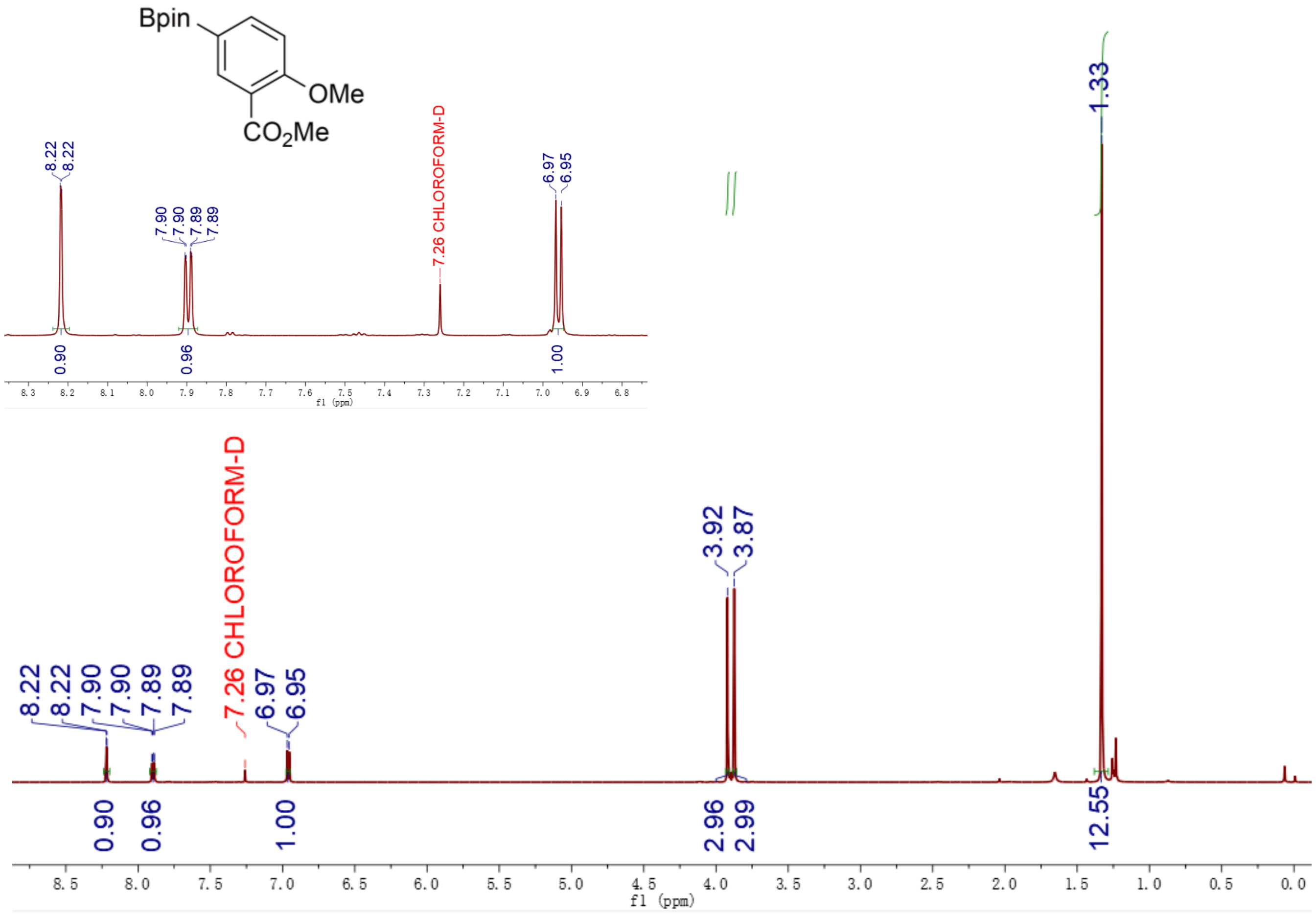

Figure S53. ^{1}H NMR spectrum of compound **2q** (400 MHz, $CDCl_3$)

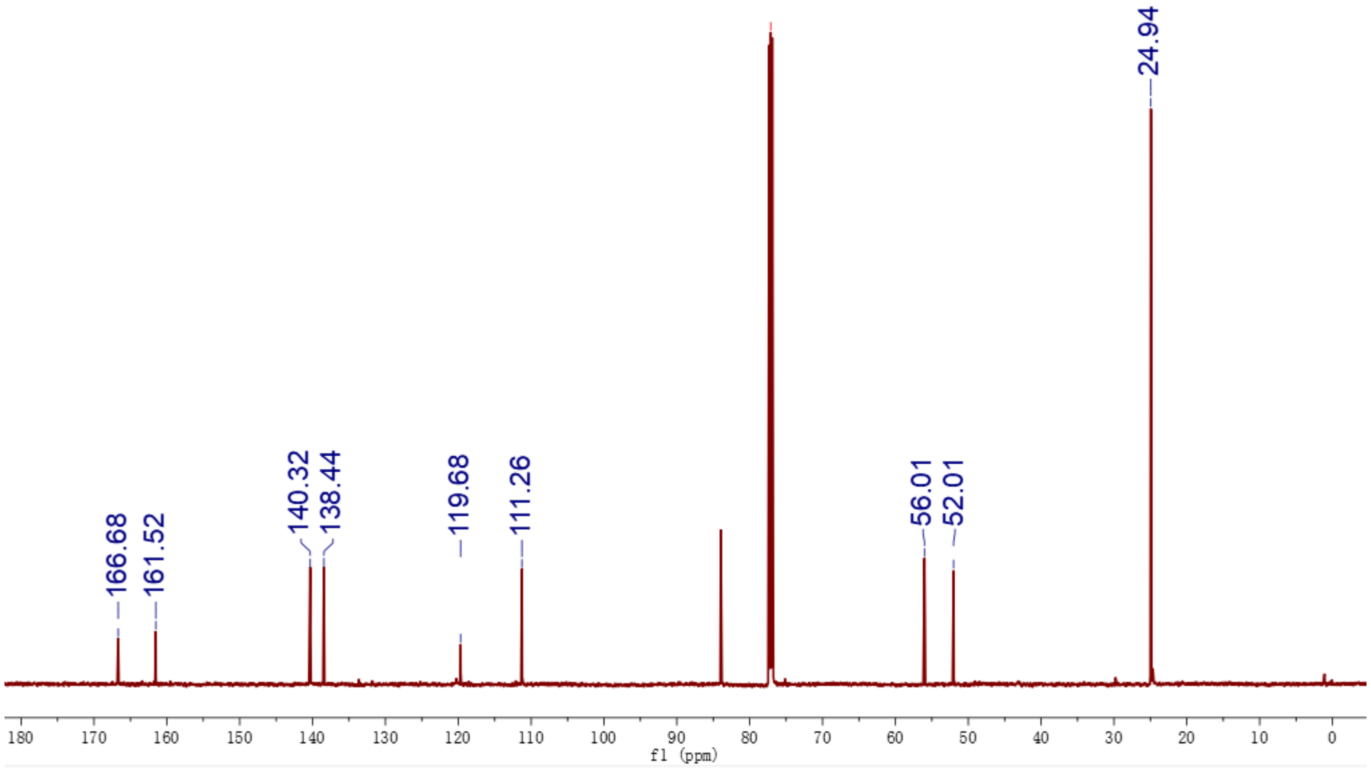

Figure S54. ^{13}C NMR spectrum of compound **2q** (101 MHz, $CDCl_3$)

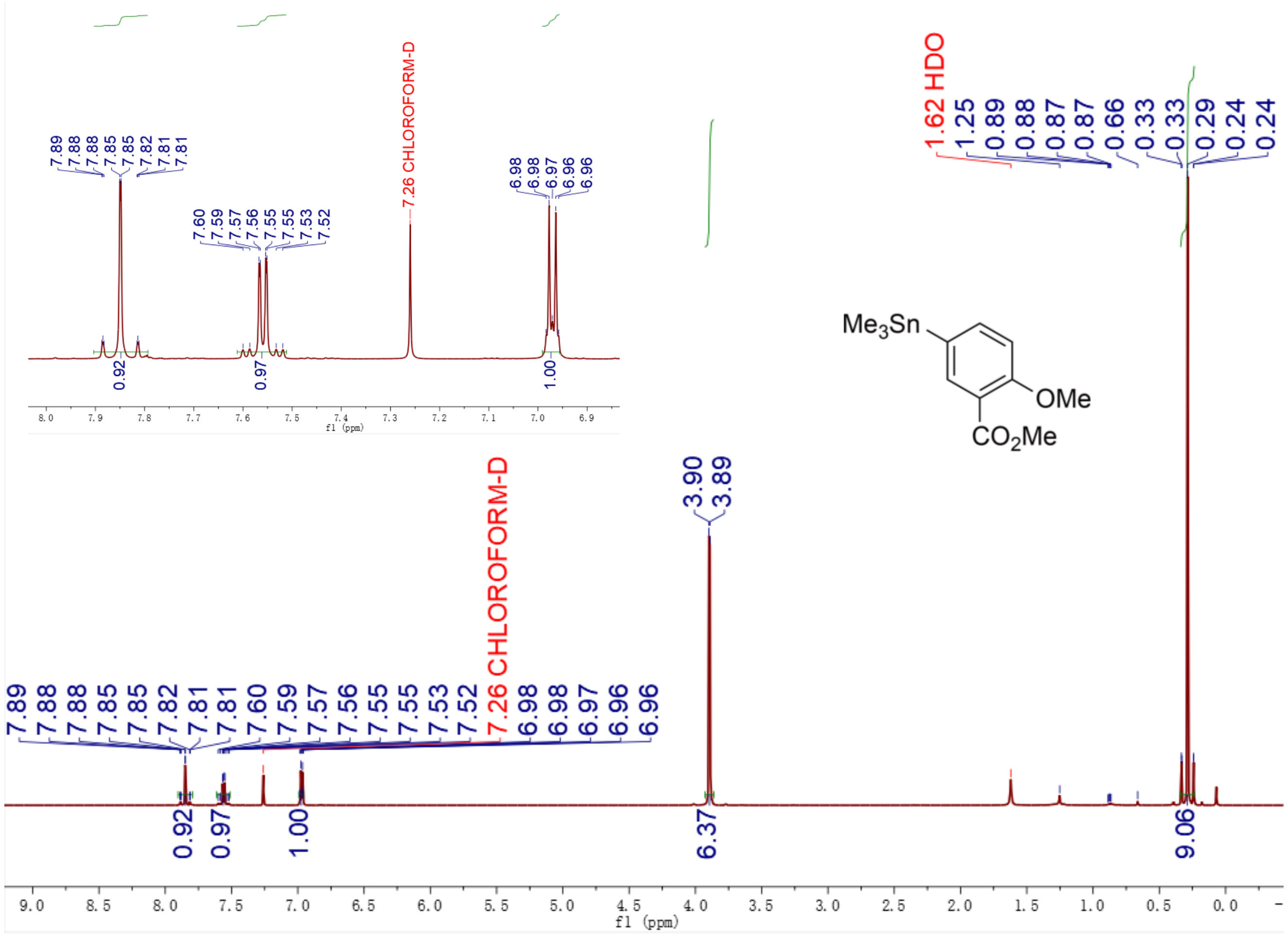

Figure S55. ^{1}H NMR spectrum of compound **2r** (400 MHz, $CDCl_3$)

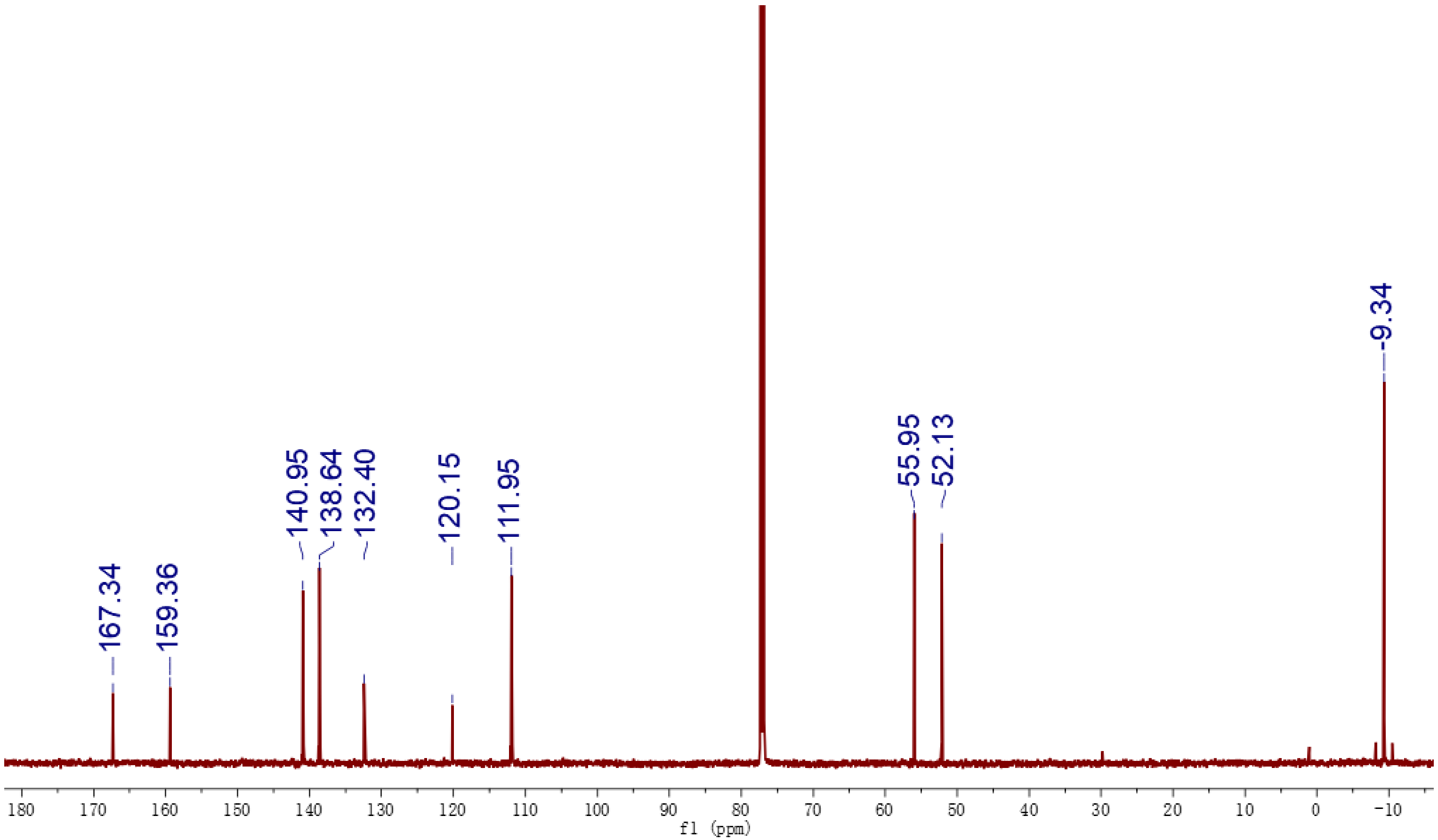

Figure S56. ^{13}C NMR spectrum of compound **2r** (101 MHz, $CDCl_3$)

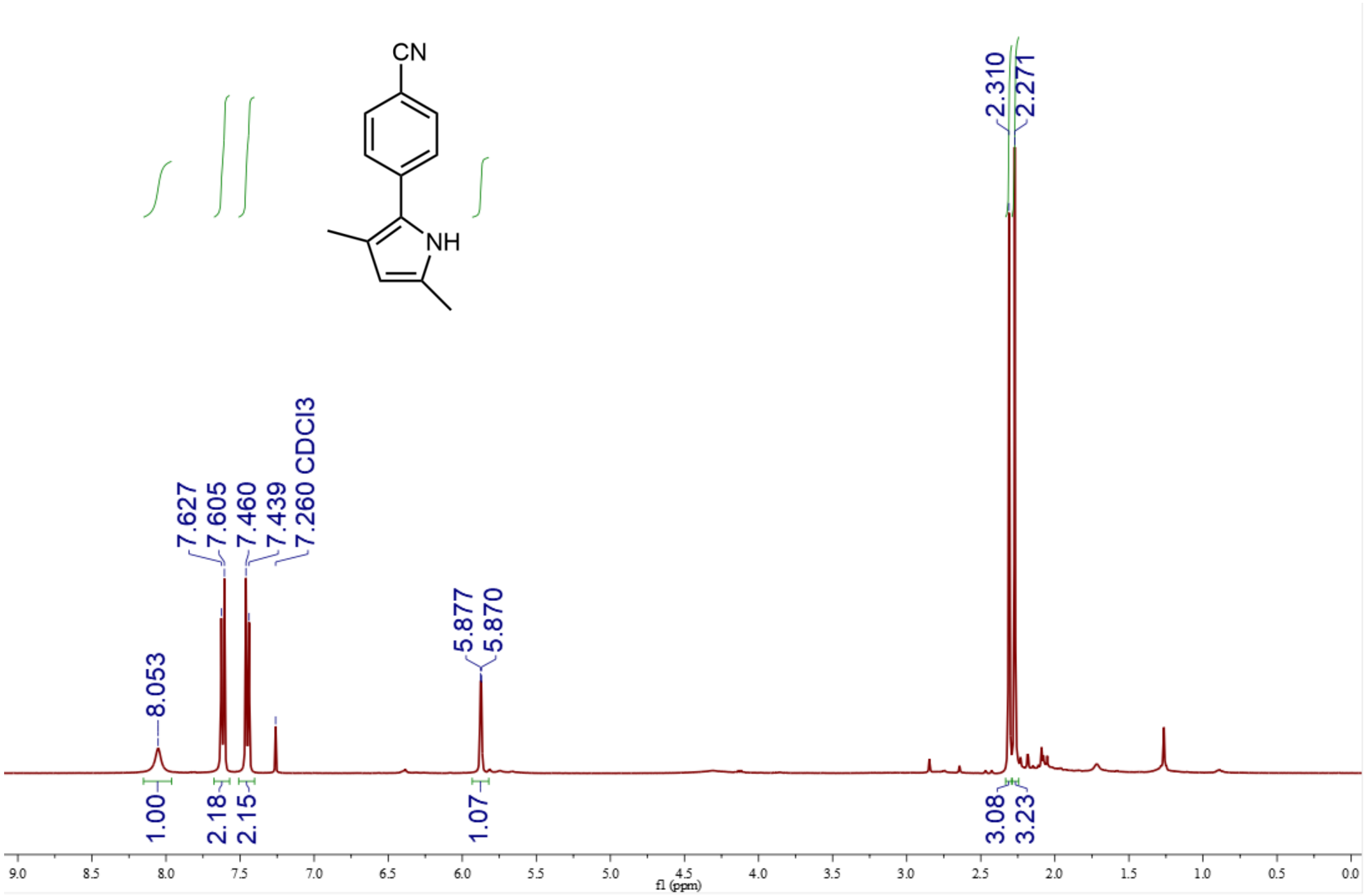

Figure S57. ^{1}H NMR spectrum of compound **2s** (400 MHz, $CDCl_3$)

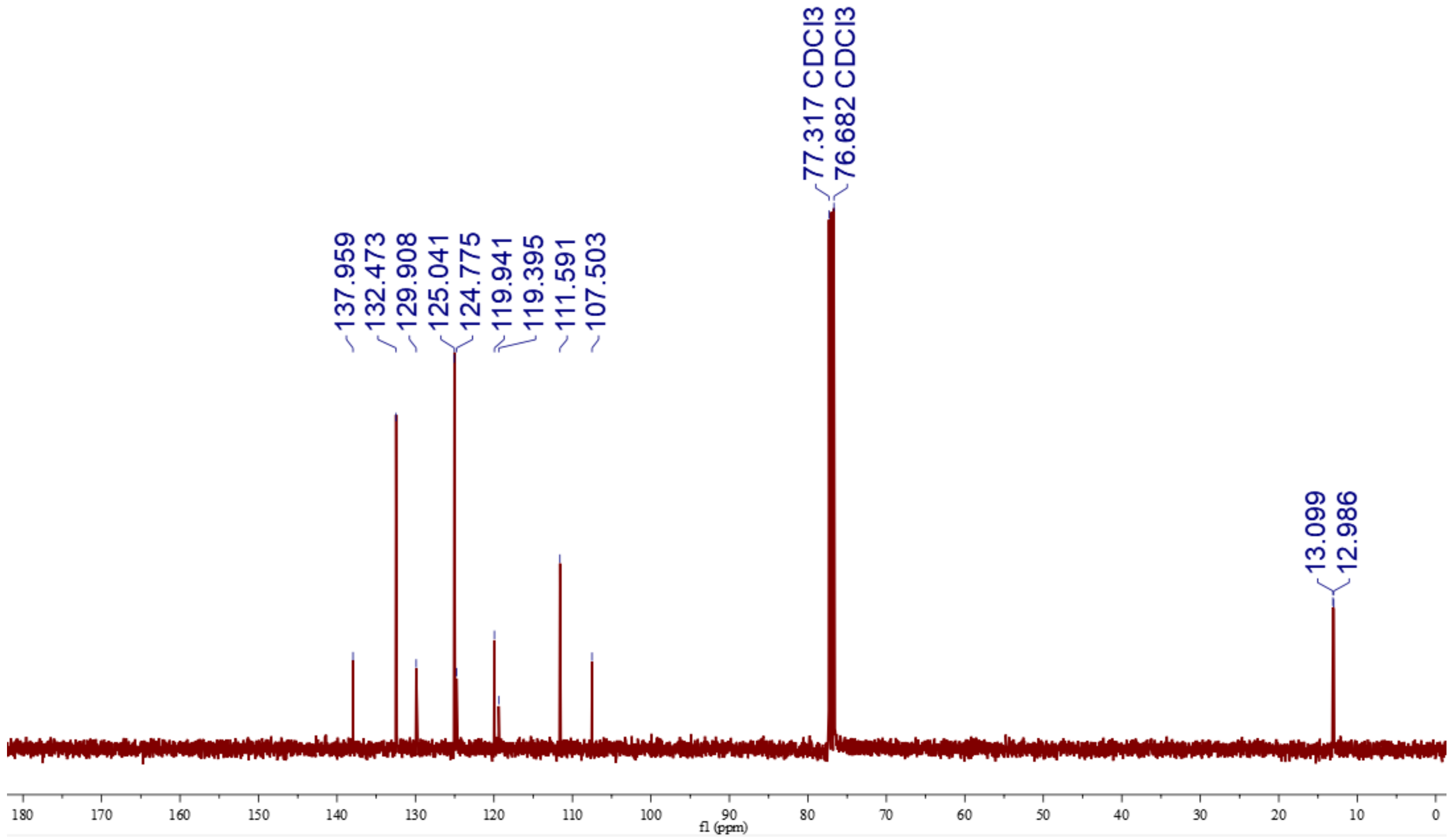

Figure S58. ^{13}C NMR spectrum of compound **2s** (101 MHz, $CDCl_3$)

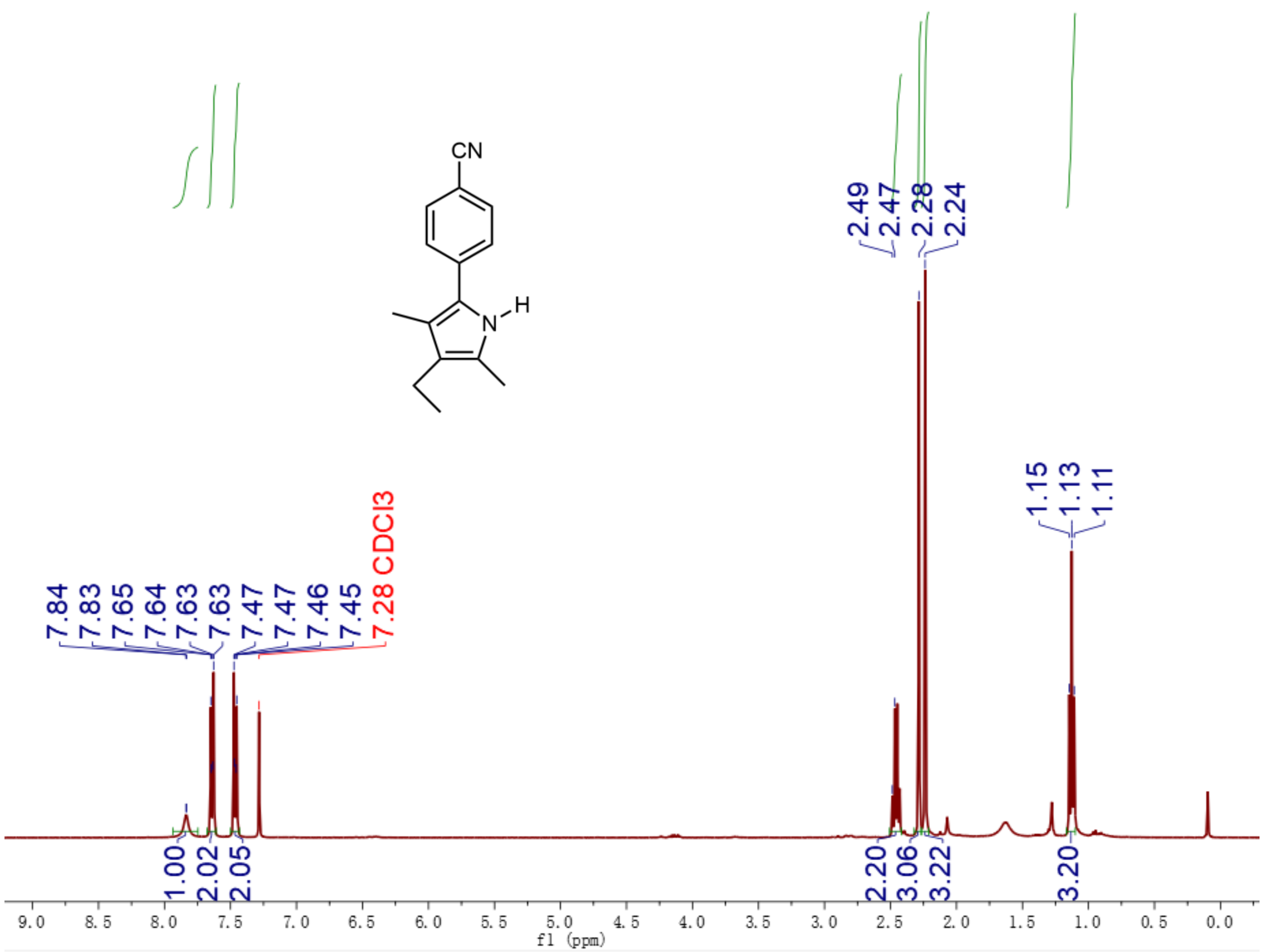

Figure S59. ^{1}H NMR spectrum of compound **2t** (400 MHz, $CDCl_3$)

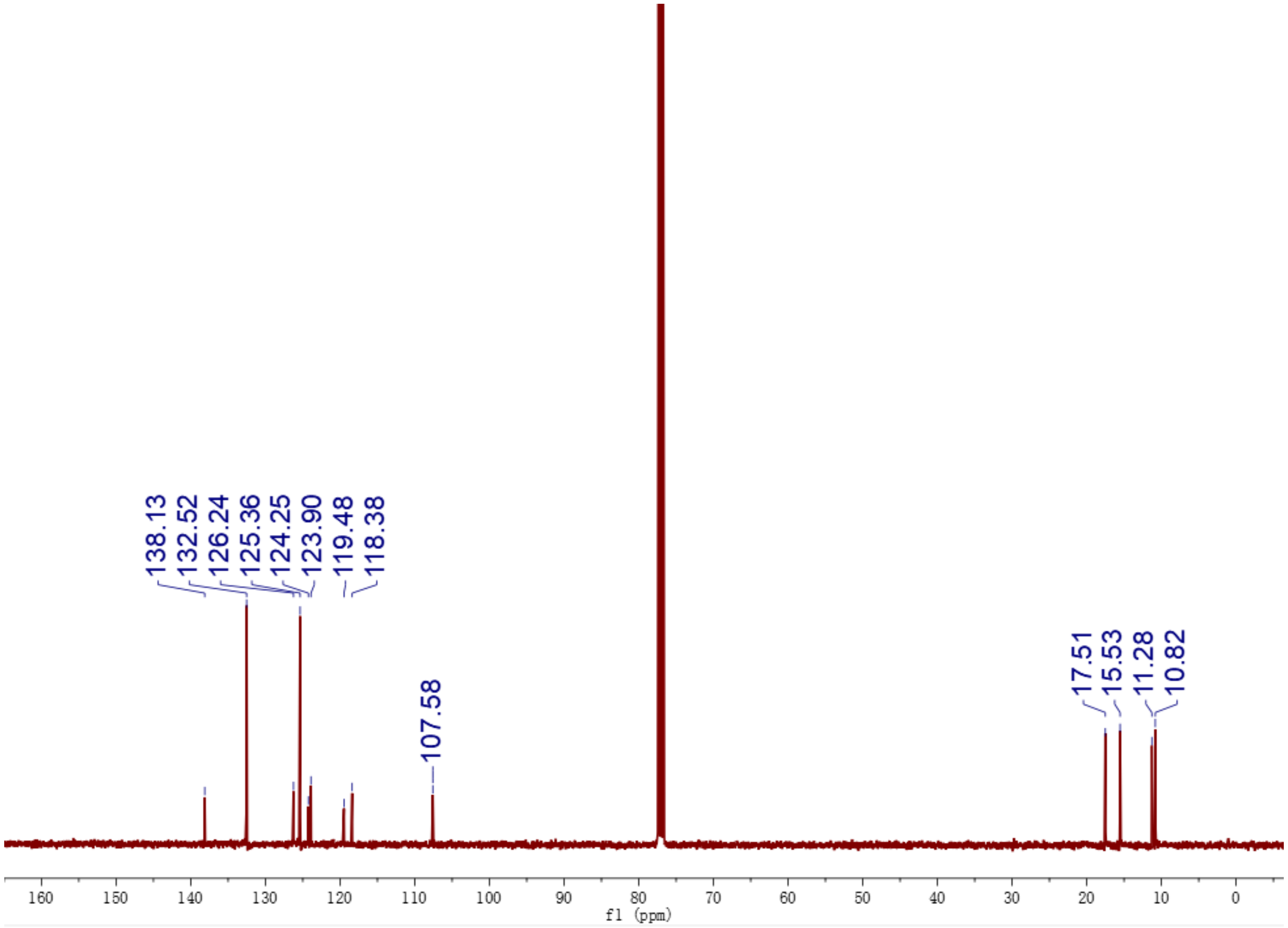

Figure S60. ^{13}C NMR spectrum of compound **2t** (101 MHz, $CDCl_3$)

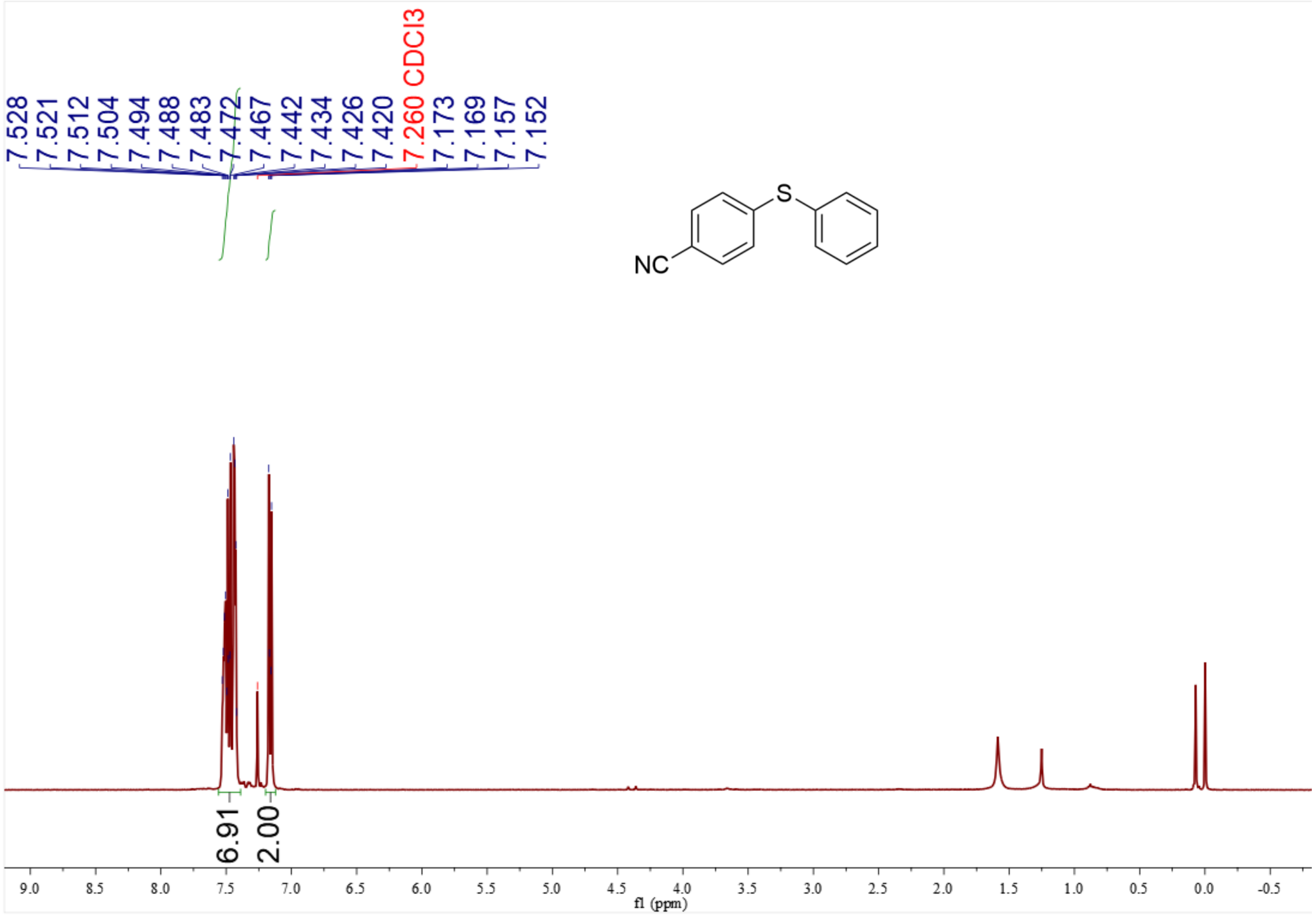

Figure S61. ^{1}H NMR spectrum of compound **2u** (400 MHz, $CDCl_3$)

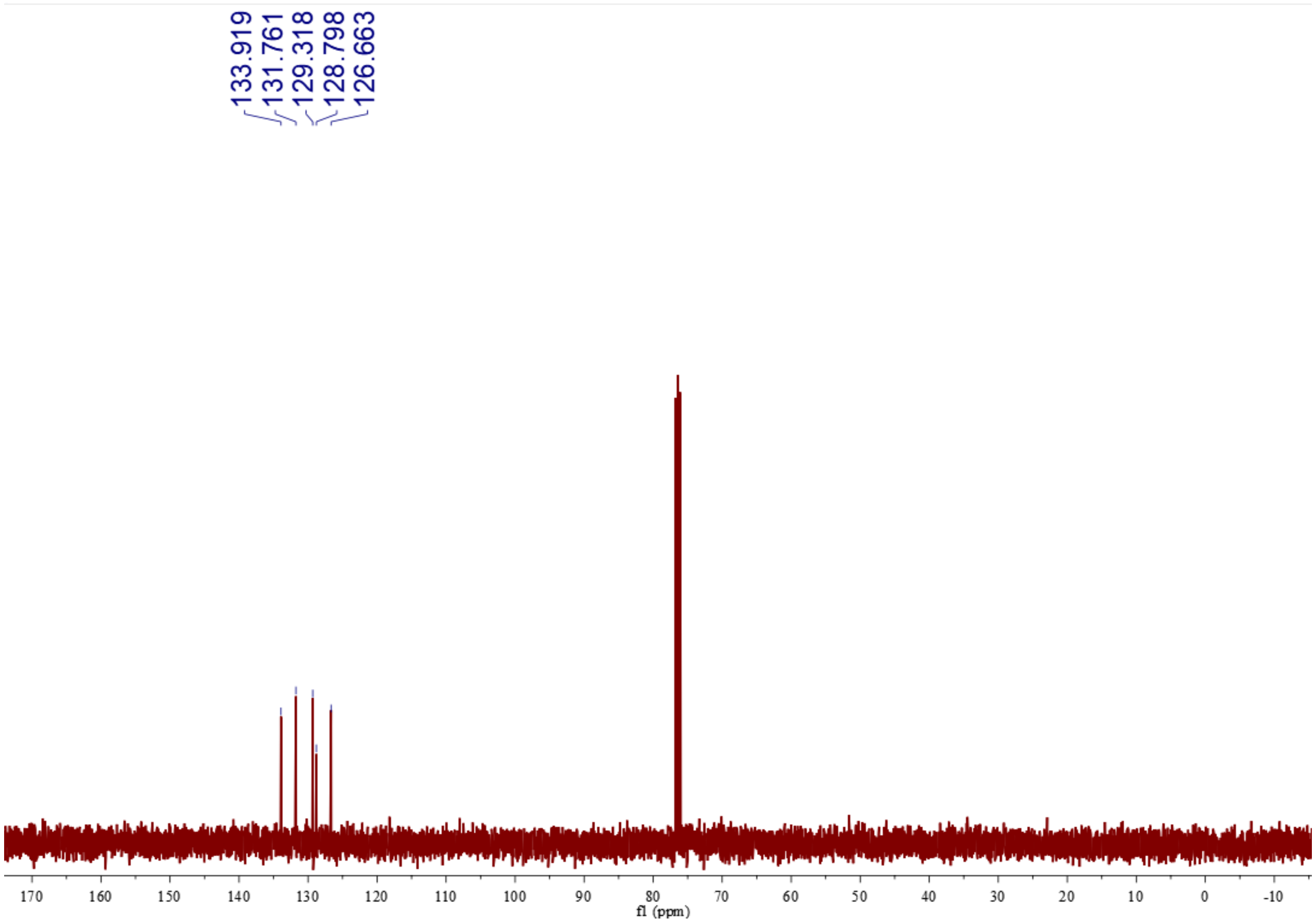

Figure S62. ^{13}C NMR spectrum of compound **2u** (101 MHz, $CDCl_3$)

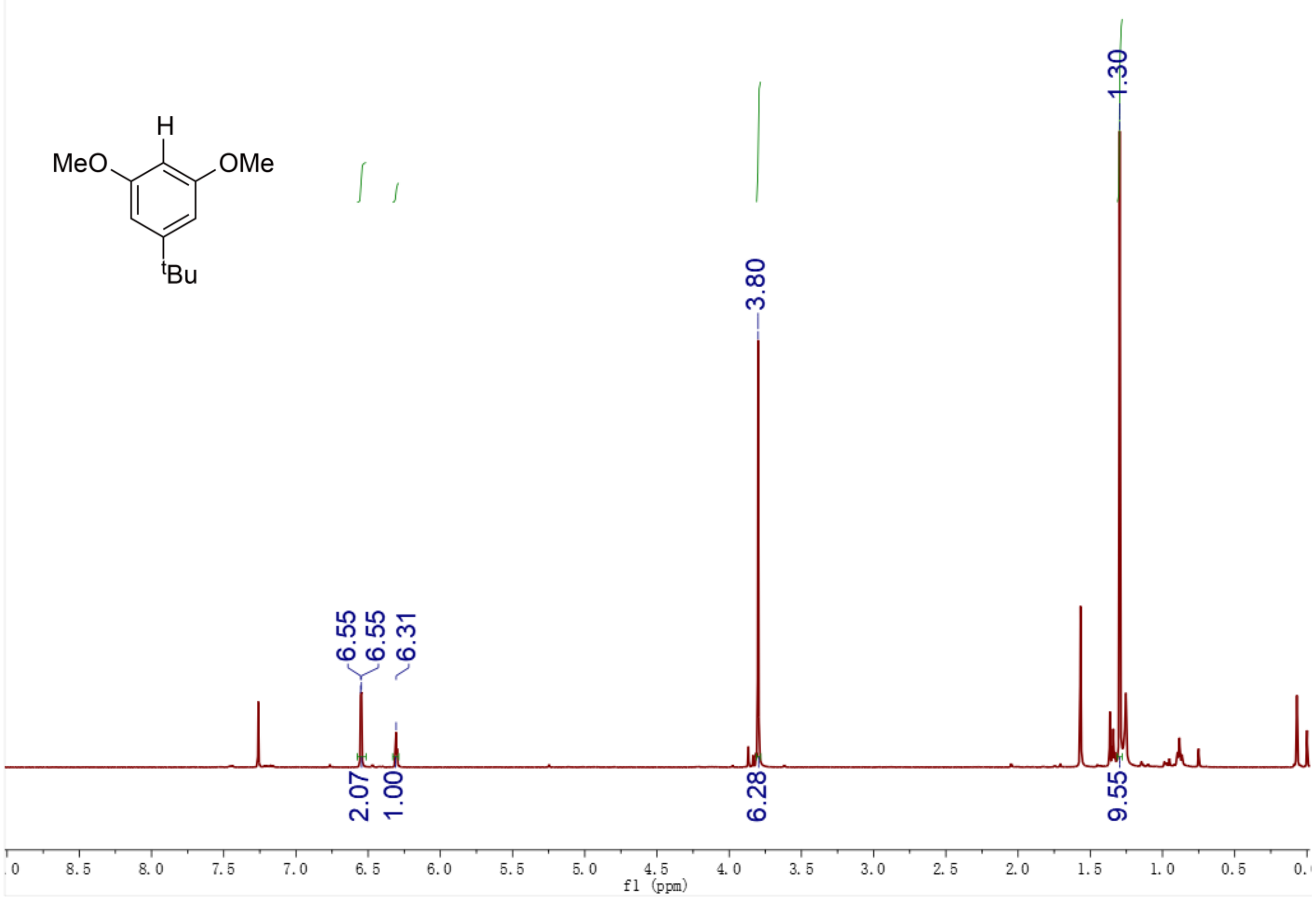

Figure S63. ^{1}H NMR spectrum of compound **4a** (400 MHz, $CDCl_3$)

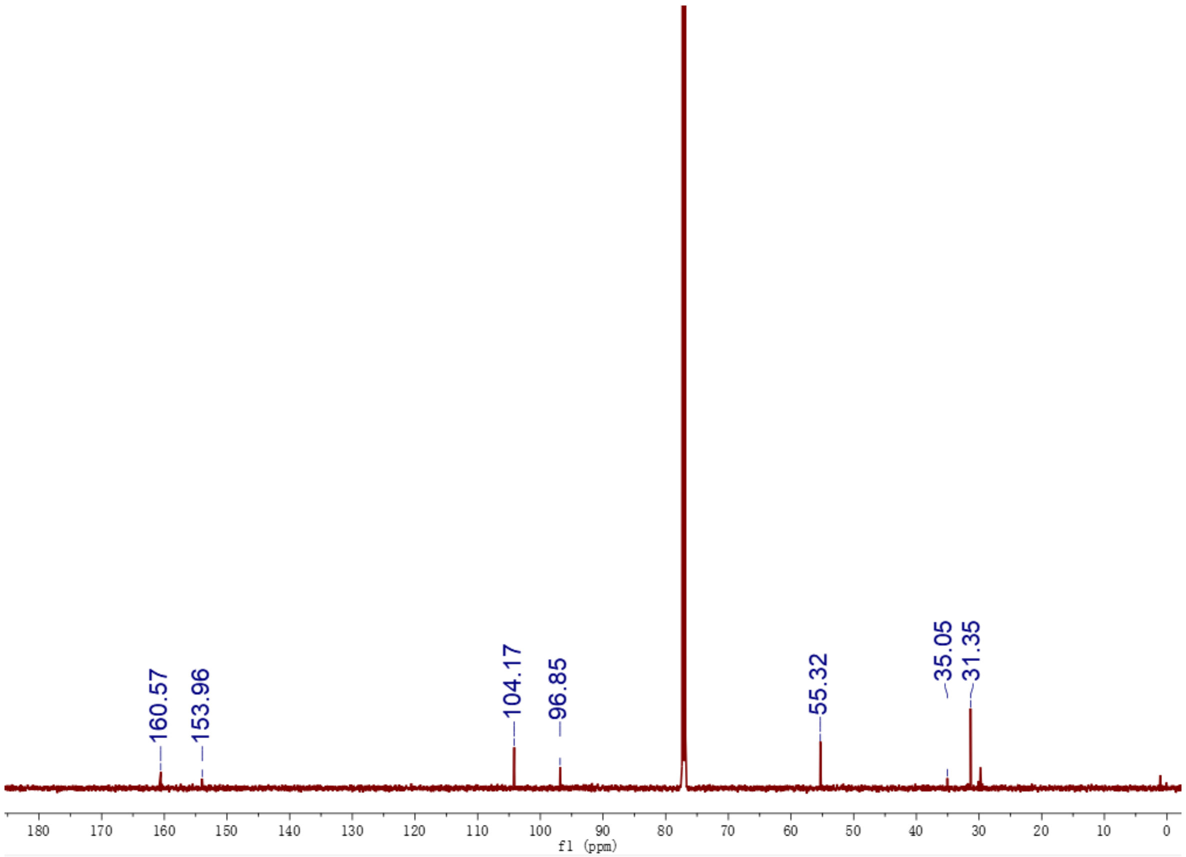

Figure S64. ^{13}C NMR spectrum of compound **4a** (101 MHz, $CDCl_3$)

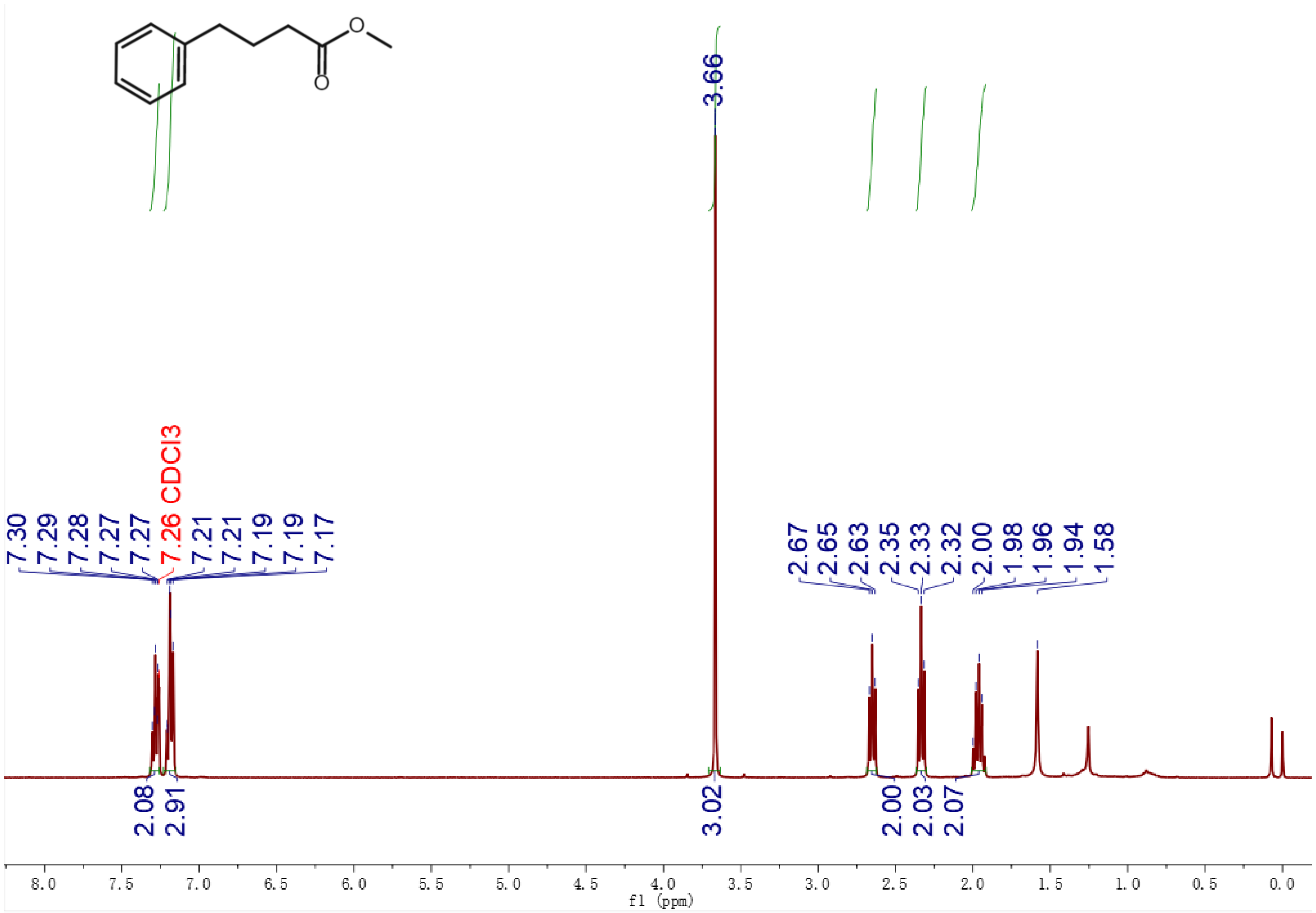

Figure S65. ^{1}H NMR spectrum of compound **6** (400 MHz, $CDCl_3$)

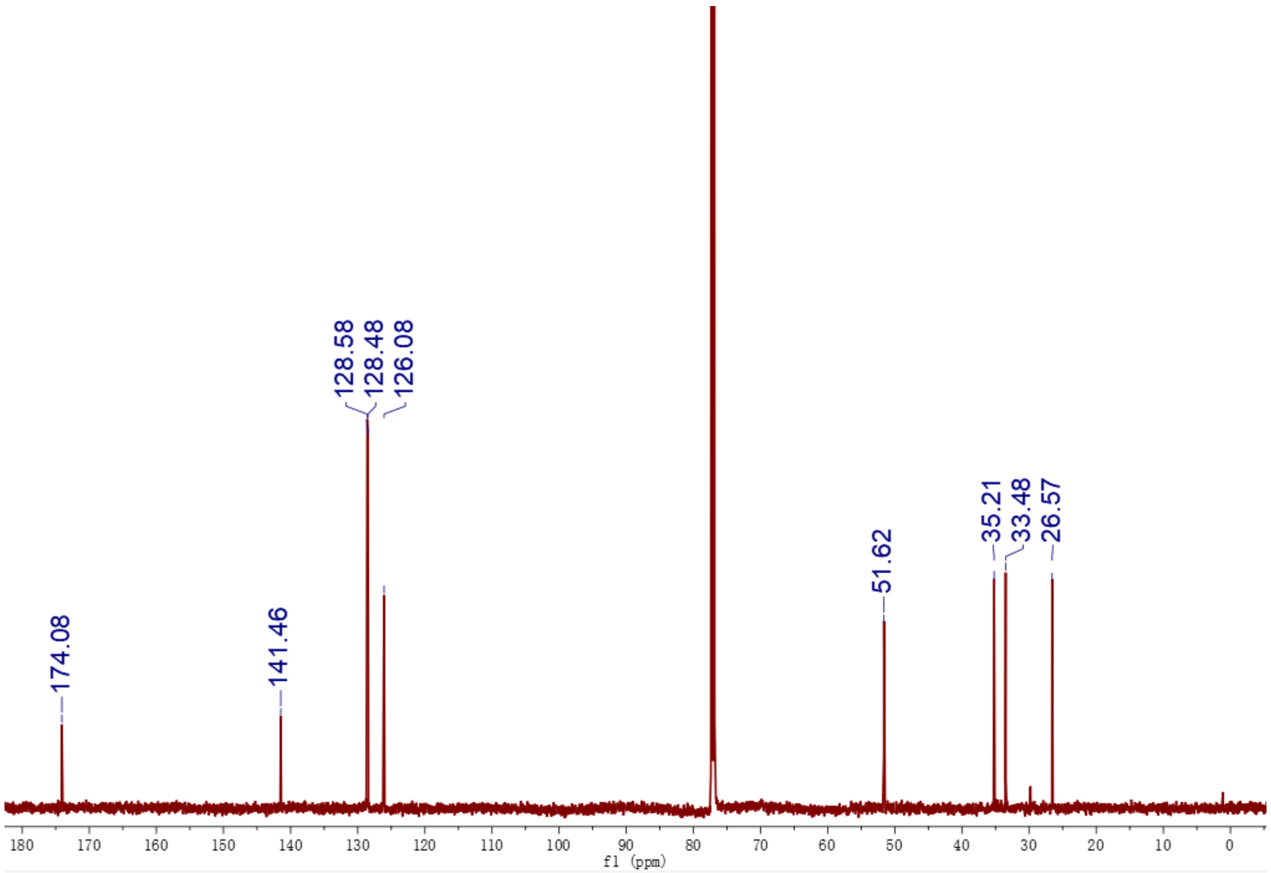

Figure S66. ^{13}C NMR spectrum of compound **6** (101 MHz, $CDCl_3$)

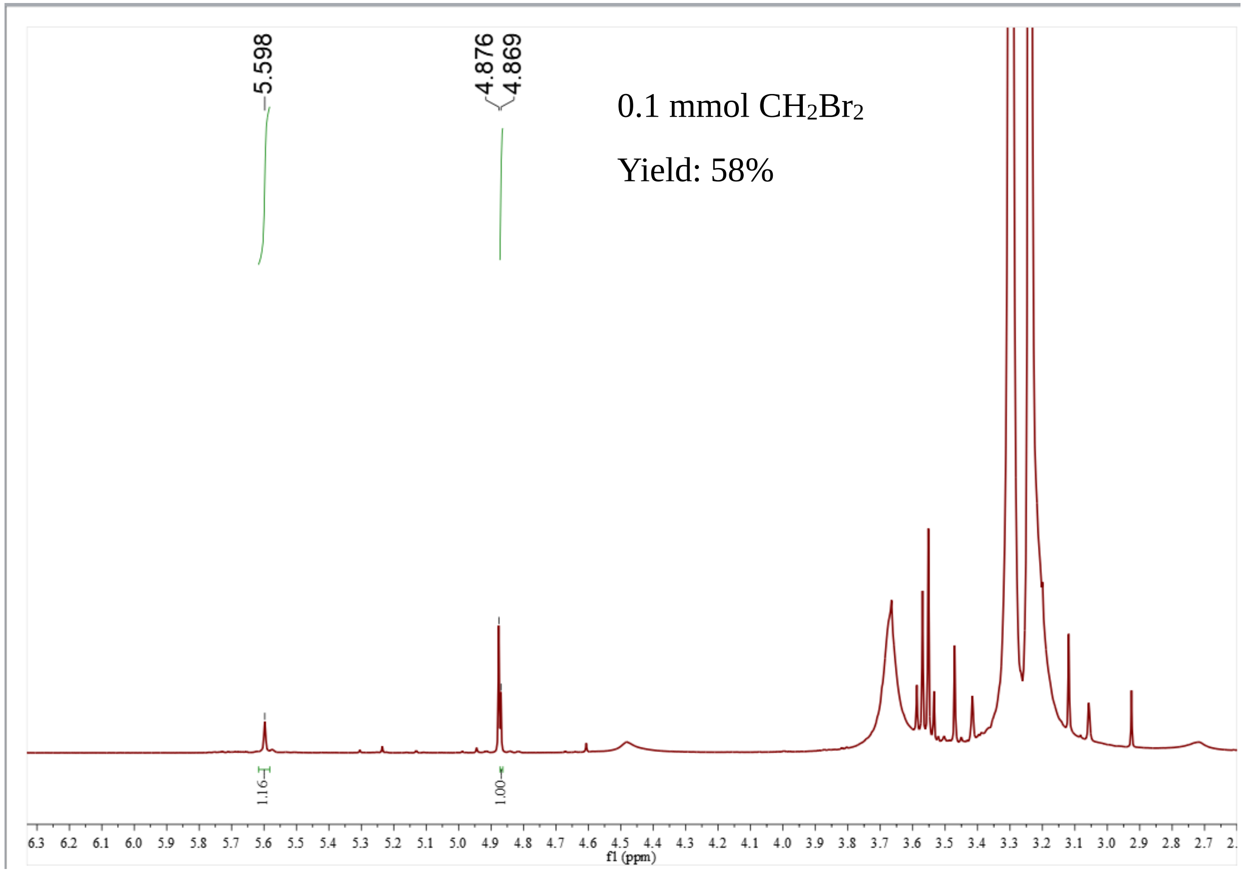

Figure S67. ^{1}H NMR spectrum of mixture contains compound **8** (400 MHz, $CDCl_3$)

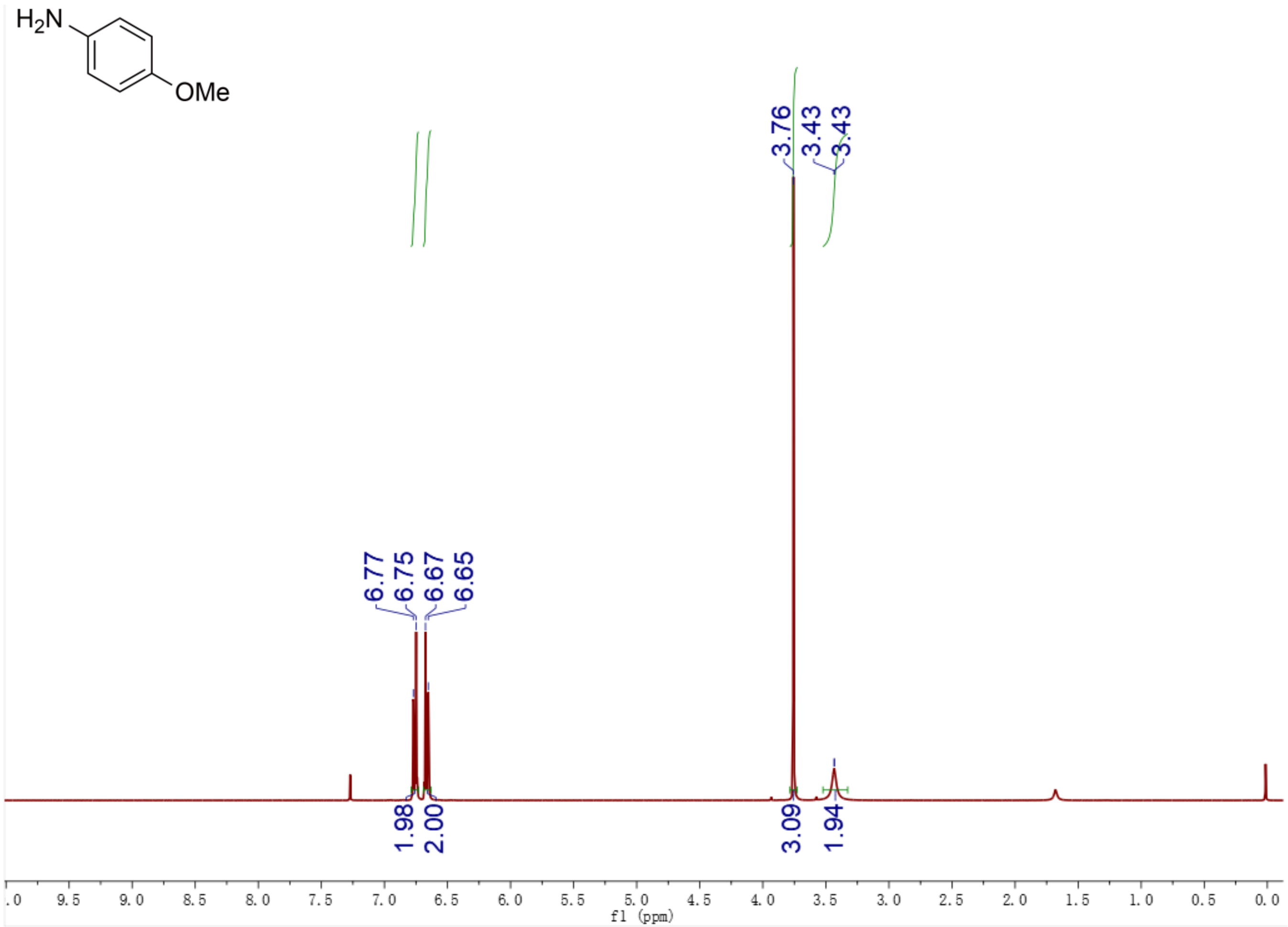

Figure S68. ^{1}H NMR spectrum of compound **10b** (400 MHz, $CDCl_3$)

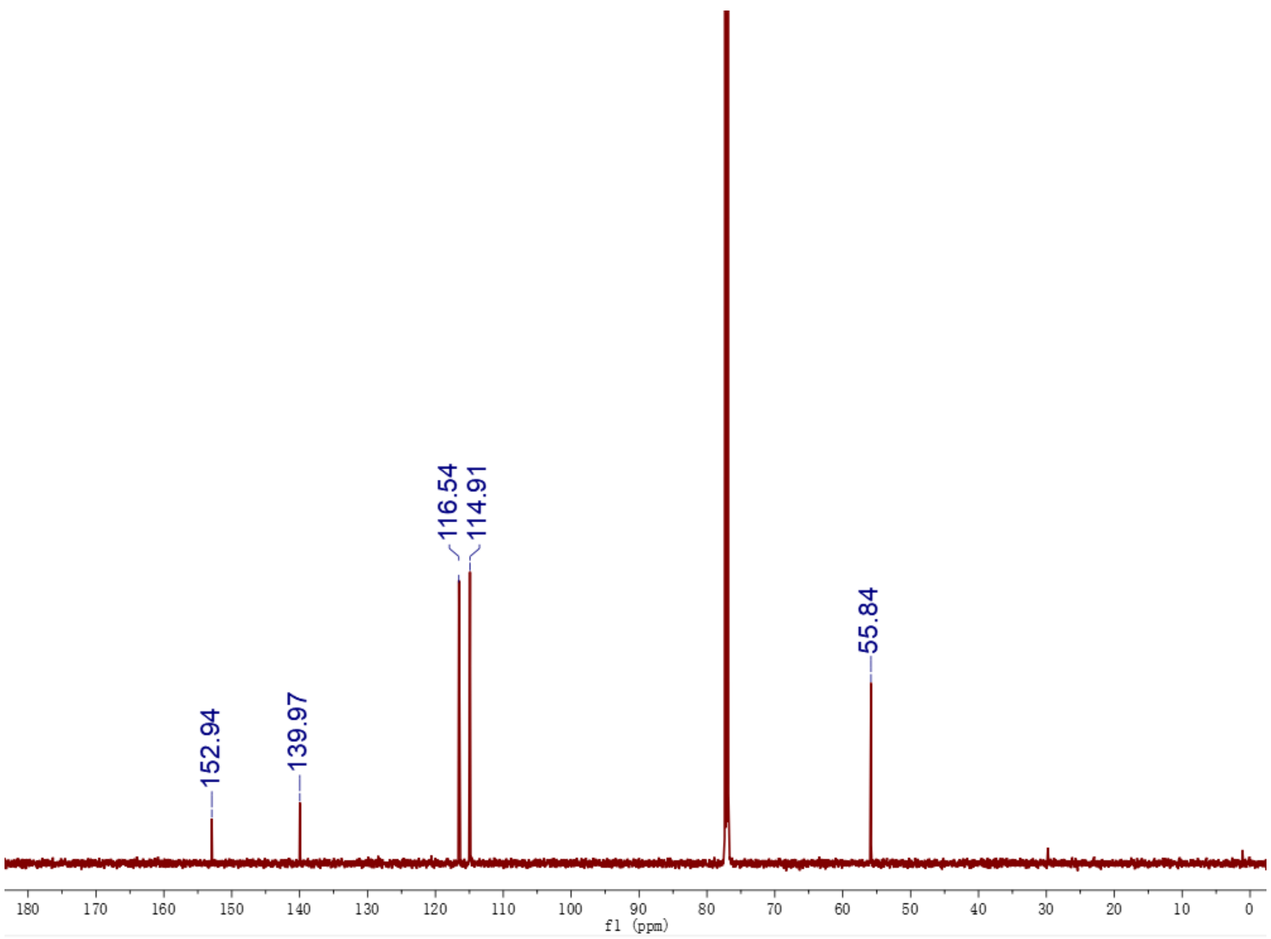

Figure S69. ^{13}C NMR spectrum of compound **10b** (101 MHz, $CDCl_3$)

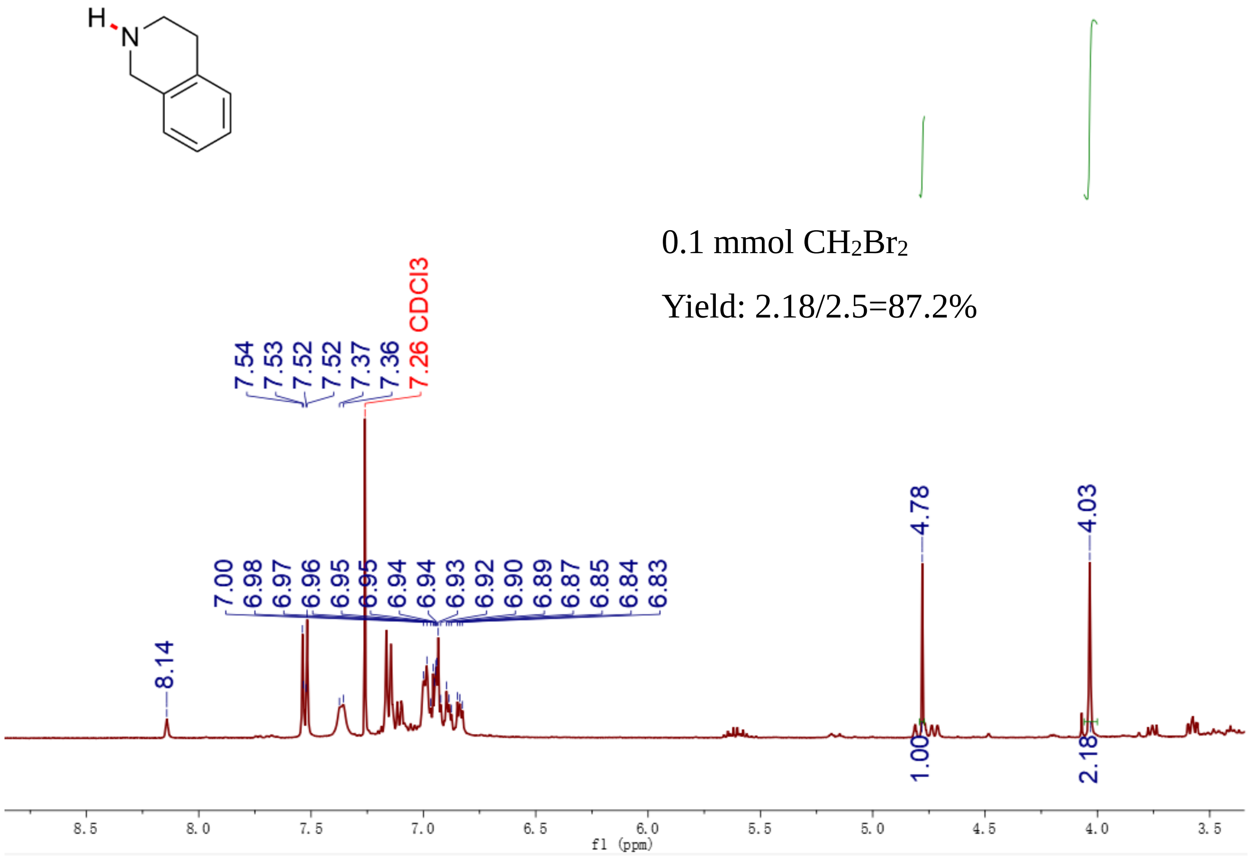

Figure S70. ^{1}H NMR spectrum of reaction mixture for **10d** (400 MHz, $CDCl_3$)

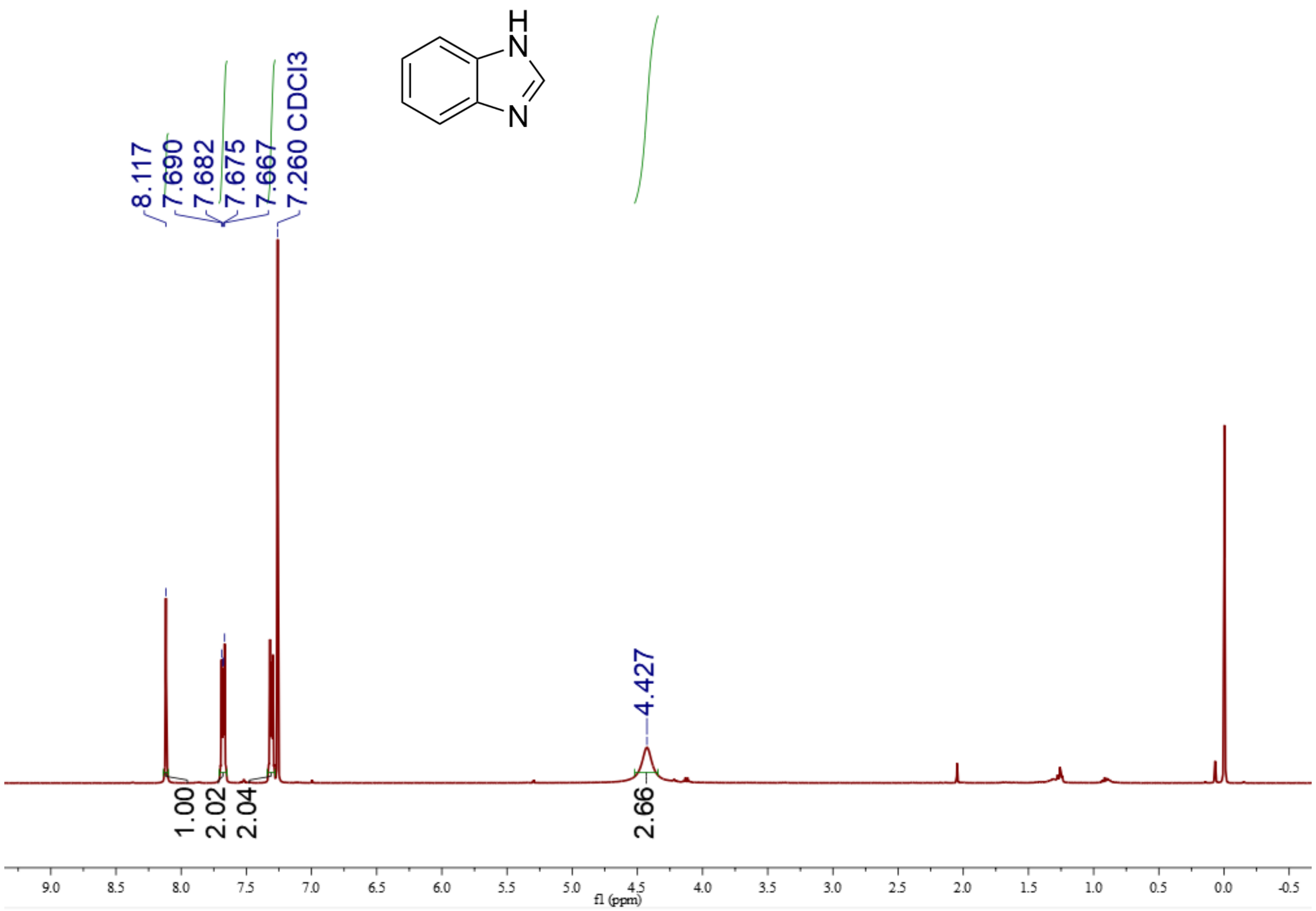

Figure S71. ^{1}H NMR spectrum of compound **10e** (400 MHz, $CDCl_3$)

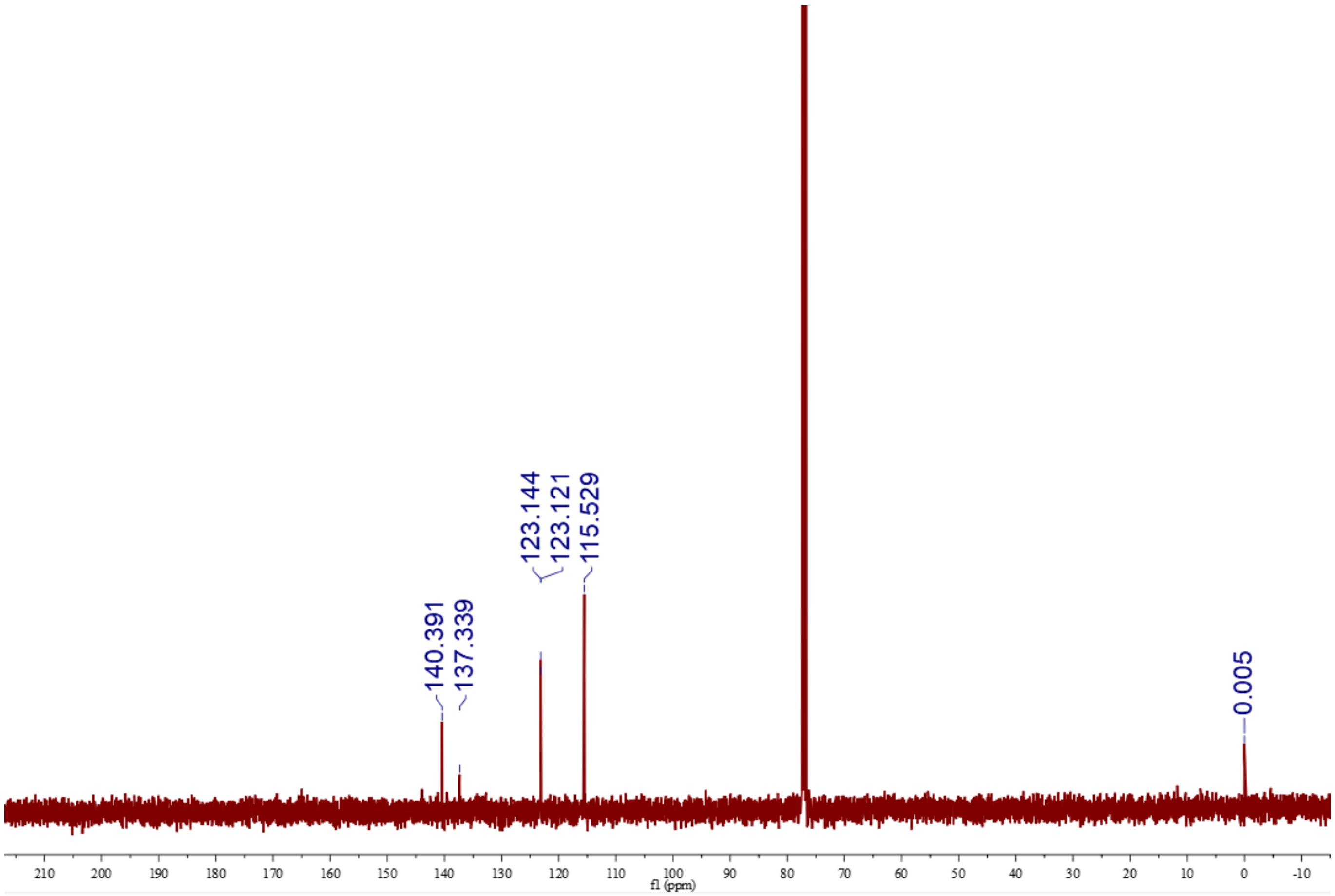

Figure S72. ^{13}C NMR spectrum of compound **10e** (101 MHz, $CDCl_3$)

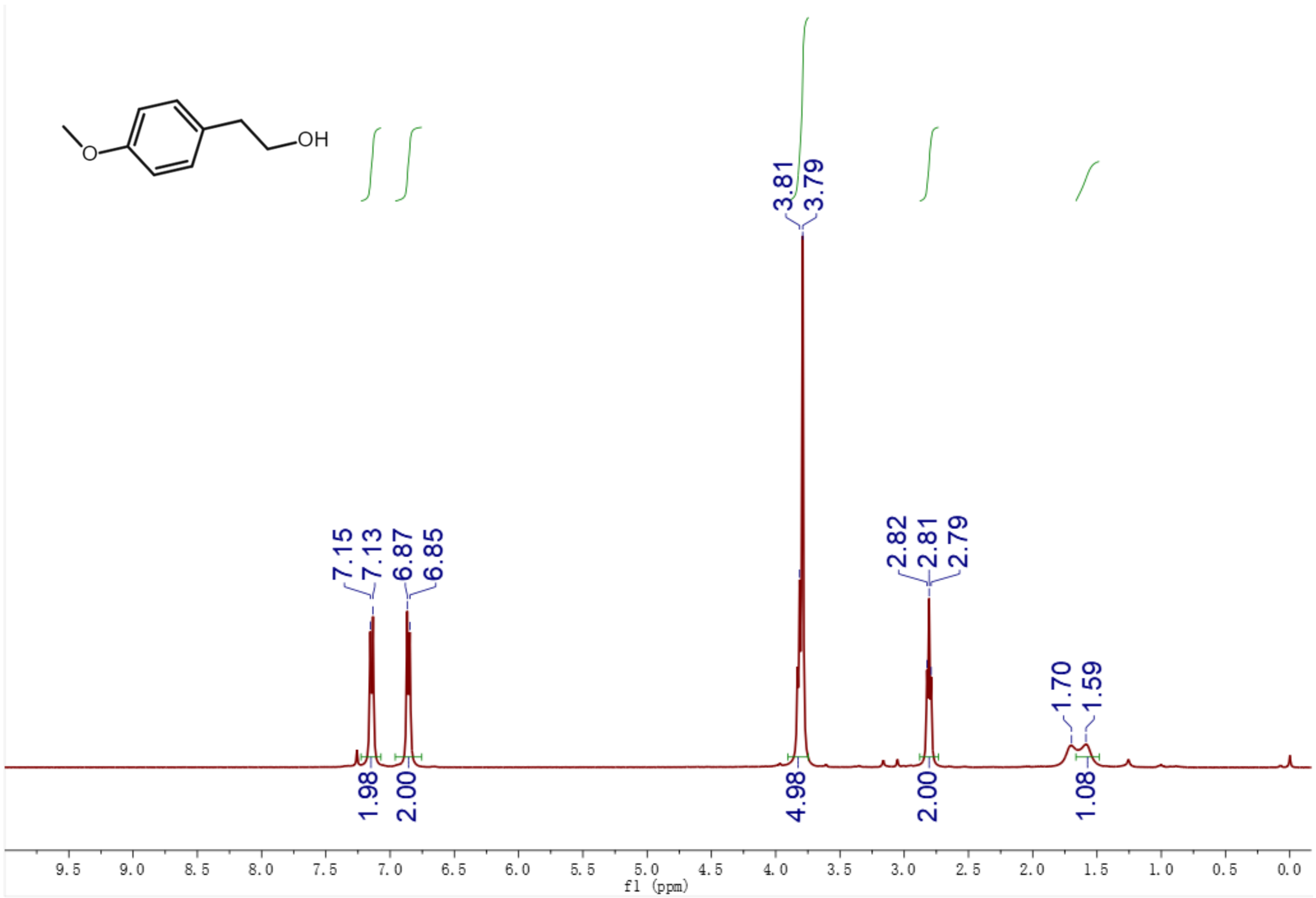

Figure S73. ^{1}H NMR spectrum of compound **14** (400 MHz, $CDCl_3$)

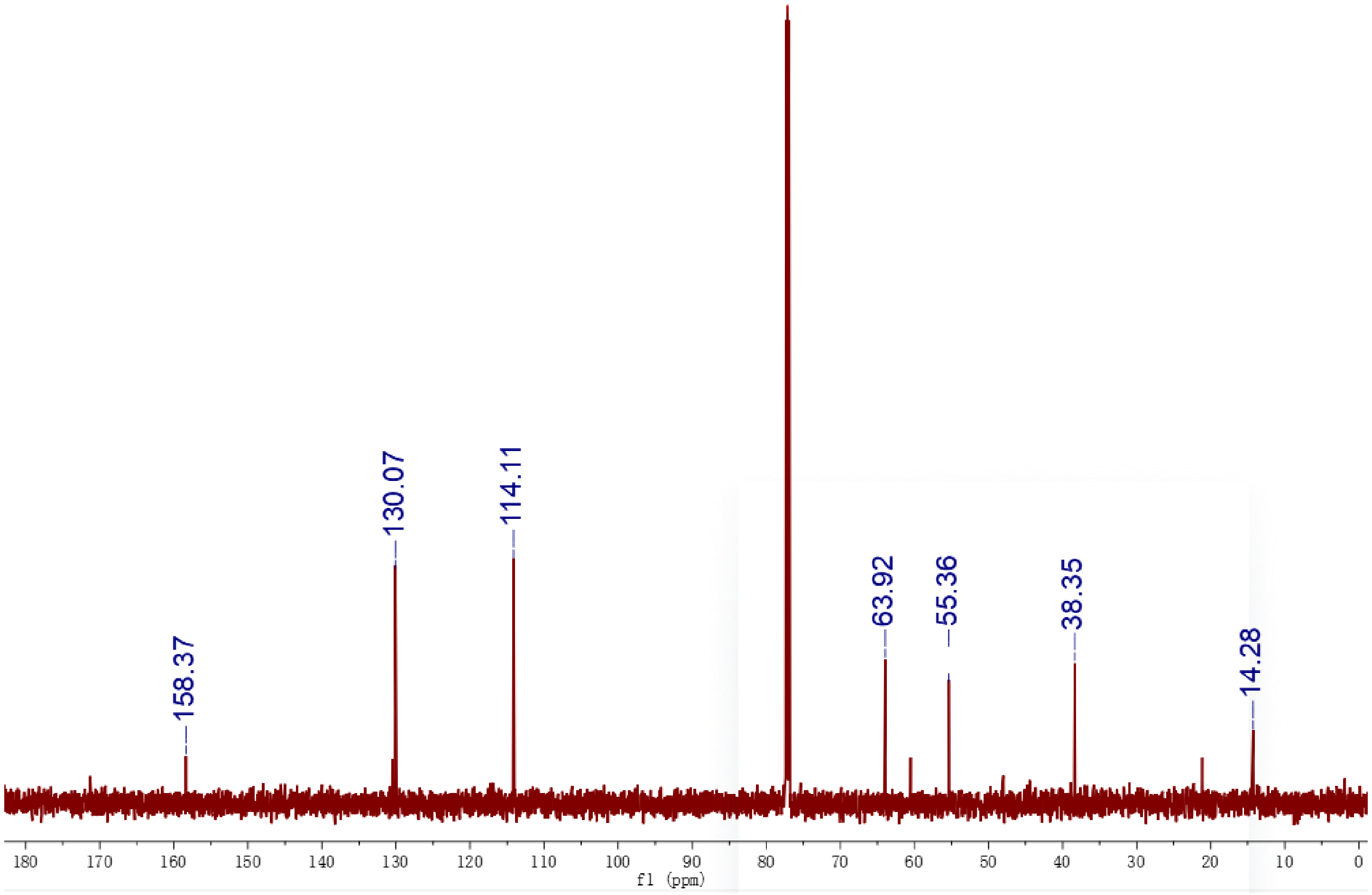

Figure S74. ^{13}C NMR spectrum of compound **12** (101 MHz, $CDCl_3$)

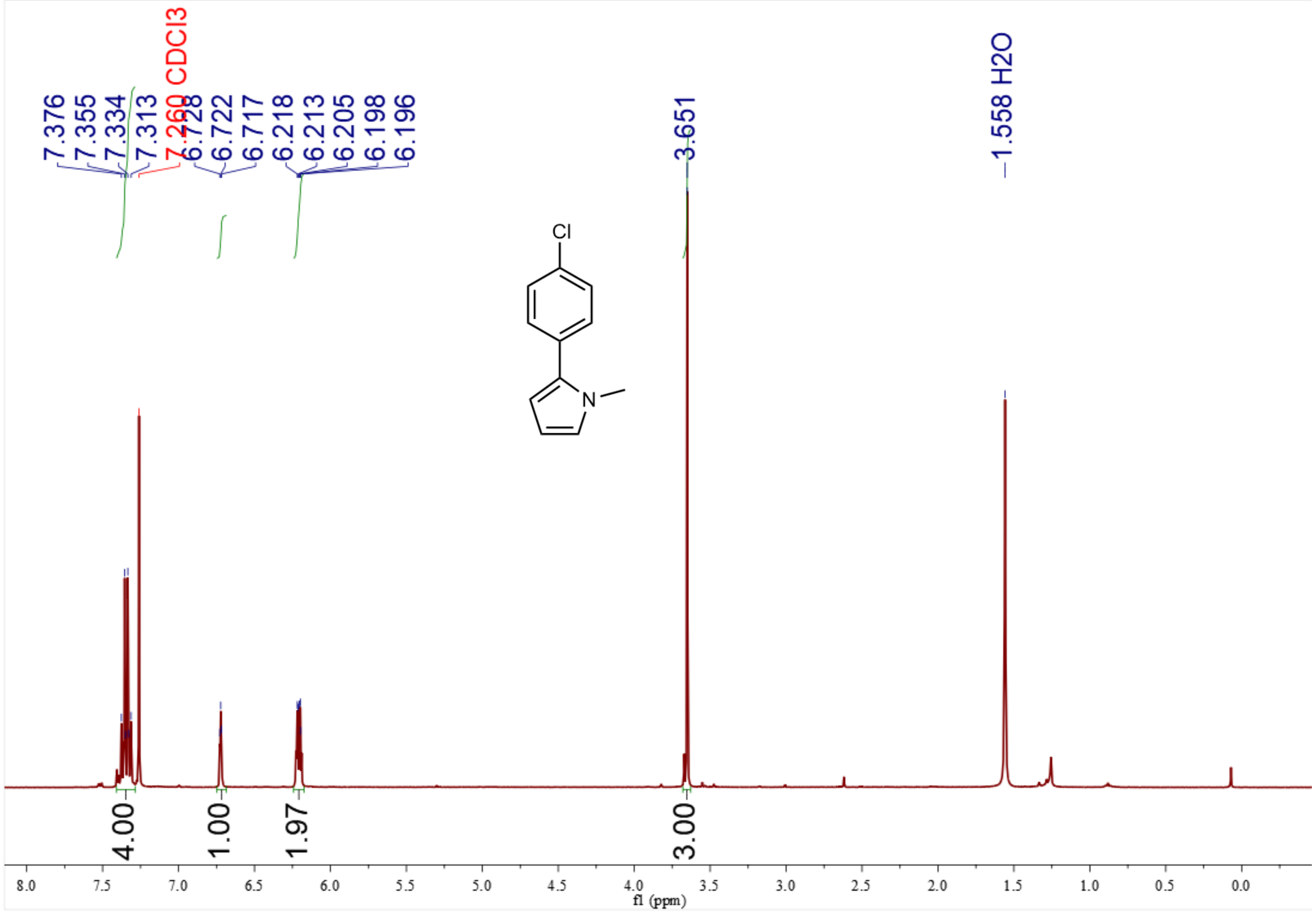

Figure S75. ^{1}H NMR spectrum of compound **16** (400 MHz, $CDCl_3$)

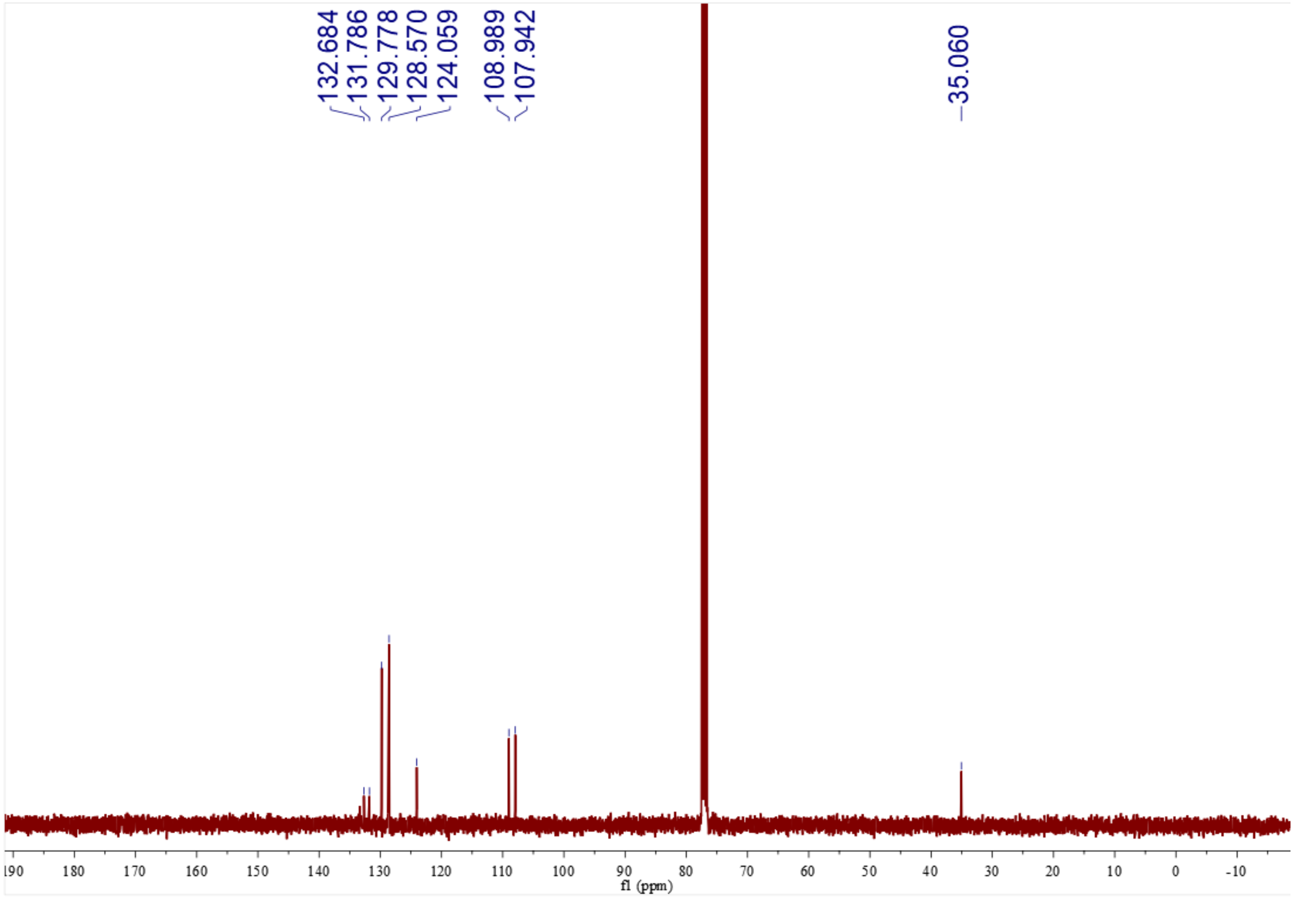

Figure S76. ^{13}C NMR spectrum of compound **16** (101 MHz, $CDCl_3$)

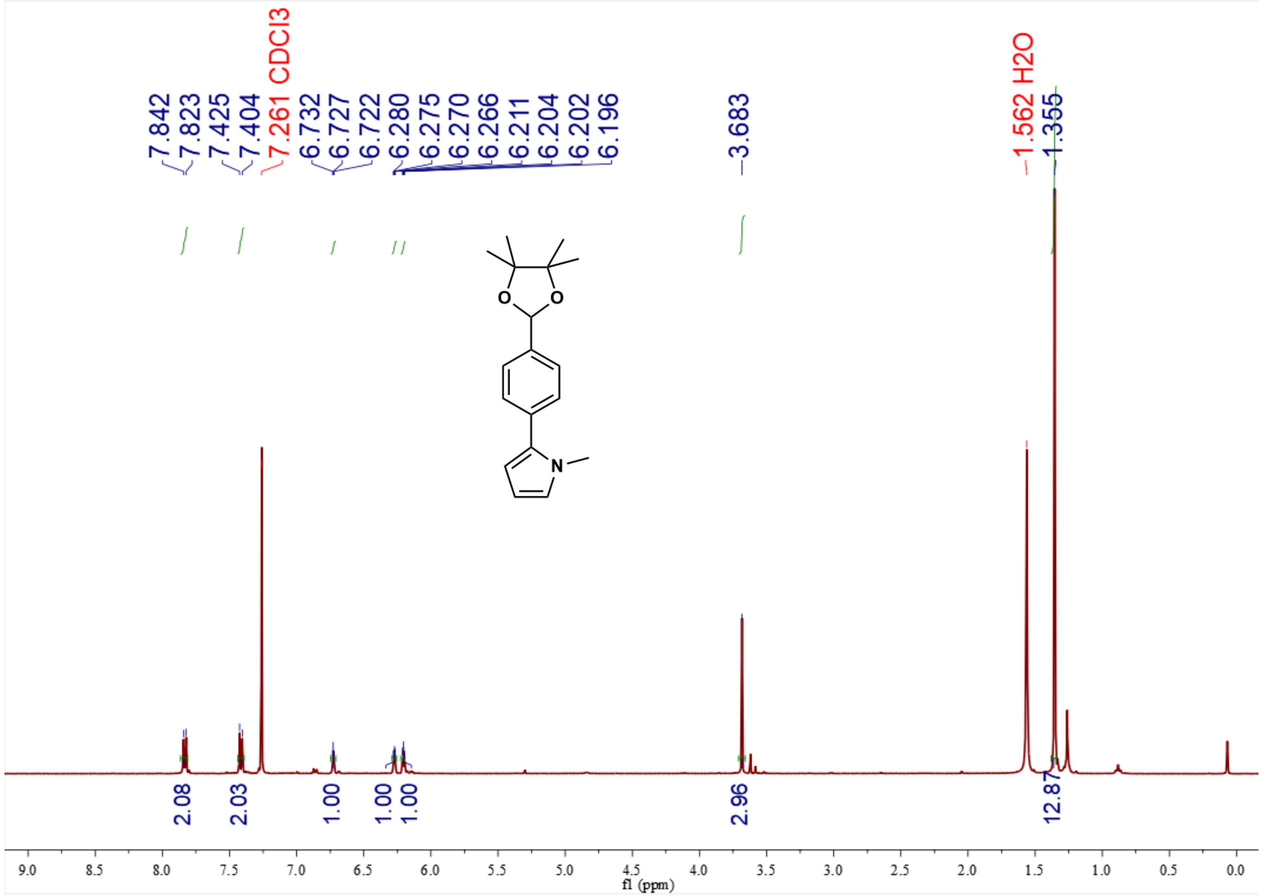

Figure S77. ^{1}H NMR spectrum of compound **17** (400 MHz, $CDCl_3$)

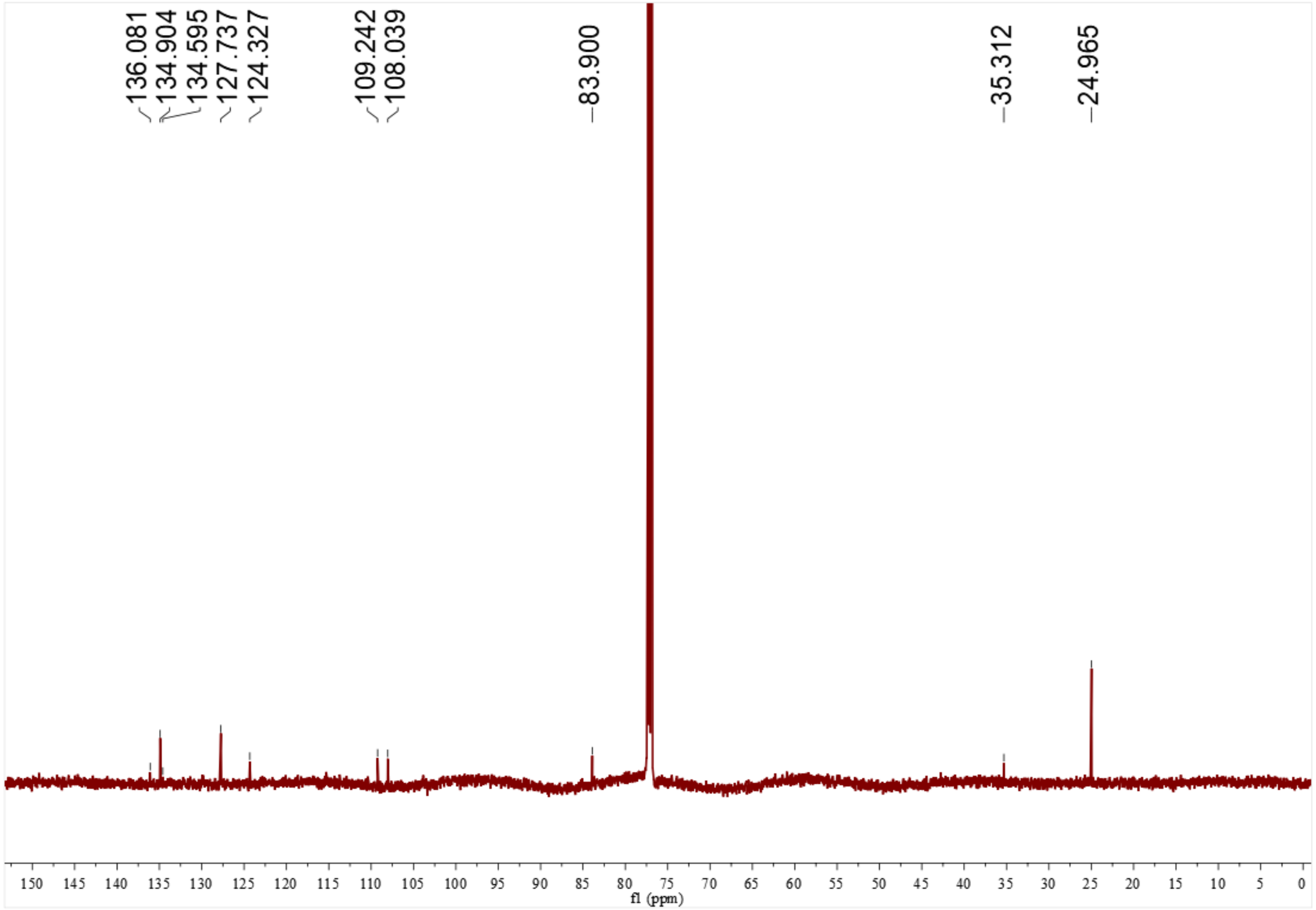

Figure S78. ^{13}C NMR spectrum of compound **17** (101 MHz, $CDCl_3$)

XI. Reference

1 Yu, W. W., Qu, L., Guo, W. & Peng, X. Experimental determination of the extinction coefficient of CdTe, CdSe, and CdS nanocrystals. *chemistry of Materials* **15**, 2854-2860 (2003).

2 Klimov, V. I. Optical Nonlinearities and Ultrafast Carrier Dynamics in Semiconductor Nanocrystals. *J. Phys. Chem. B* **104**, 6112-6123 (2000).

3 Li, M. *et al.* Slow cooling and highly efficient extraction of hot carriers in colloidal perovskite nanocrystals. *Nat Commun* **8**, 14350 (2017). https://doi.org:10.1038/ncomms14350

4 Pavlovska, T. *et al.* Tuning Deazaflavins Towards Highly Potent Reducing Photocatalysts Guided by Mechanistic Understanding - Enhancement of the Key Step by the Internal Heavy Atom Effect. *Chemistry* **28**, e202200768 (2022). https://doi.org:10.1002/chem.202200768

5 Faldt, A., C. Krebs, F. & Thorup, N. Synthesis, structure and properties of various molecules based on the 4,8,12-trioxa-4,8,12,12c-tetrahydrodibenzo[cd,mn]pyrene system with an evaluation of the effect differing molecular substitution patterns has on the space group symmetry. *Journal of the Chemical Society, Perkin Transactions 2*, 2219-2228 (1997). https://doi.org:10.1039/A703641H

6 Widness, J. K. *et al.* CdS Quantum Dots as Potent Photoreductants for Organic Chemistry Enabled by Auger Processes. *Journal of the American Chemical Society* **144**, 12229-12246 (2022). https://doi.org:10.1021/jacs.2c03235

7 MacKenzie, I. A. *et al.* Discovery and characterization of an acridine radical photoreductant. *Nature* **580**, 76-80 (2020). https://doi.org:10.1038/s41586-020-2131-1

8 Zhao, Y. *et al.* Selective synthesis of pyrrolo[1,2-a]azepines or 4,6-dicarbonyl indoles via tandem reactions of alkynones with pyrrole derivatives. *Org Biomol Chem* **15**, 6328-6332 (2017). https://doi.org:10.1039/c7ob01516j
